# High *Campylobacter* diversity in retail chicken: epidemiologically important strains may be missed with current sampling methods

**DOI:** 10.1017/S0950268824000906

**Published:** 2024-08-22

**Authors:** Agata H. Dziegiel, Samuel J. Bloomfield, George M. Savva, Raphaëlle Palau, Nicol Janecko, John Wain, Alison E. Mather

**Affiliations:** 1 Microbes and Food Safety, Quadram Institute Bioscience, Norwich, UK; 2 Core Science Resources, Quadram Institute Bioscience, Norwich, UK; 3 Norwich Medical School, University of East Anglia, Norwich, UK

**Keywords:** antimicrobial resistance, *Campylobacter*, chicken, outbreaks, source attribution, whole genome sequencing

## Abstract

*Campylobacter* spp. are leading bacterial gastroenteritis pathogens. Infections are largely underreported, and the burden of outbreaks may be underestimated. Current strategies of testing as few as one isolate per sample can affect attribution of cases to epidemiologically important sources with high *Campylobacter* diversity, such as chicken meat. Multiple culture method combinations were utilized to recover and sequence *Campylobacter* from 45 retail chicken samples purchased across Norwich, UK, selecting up to 48 isolates per sample. Simulations based on resampling were used to assess the impact of *Campylobacter* sequence type (ST) diversity on outbreak detection. *Campylobacter* was recovered from 39 samples (87%), although only one sample was positive through all broth, temperature, and plate combinations. Three species were identified (*Campylobacter jejuni*, *Campylobacter coli*, and *Campylobacter lari*), and 33% of samples contained two species. Positive samples contained 1–8 STs. Simulation revealed that up to 87 isolates per sample would be required to detect 95% of the observed ST diversity, and 26 isolates would be required for the average probability of detecting a random theoretical outbreak ST to reach 95%. An optimized culture approach and selecting multiple isolates per sample are essential for more complete *Campylobacter* recovery to support outbreak investigation and source attribution.

## Introduction

*Campylobacter* spp. are the most common cause of bacterial gastroenteritis, with over 500 000 estimated annual cases in the United Kingdom [1], and high economic costs related to healthcare and loss of productivity [2]. The species most often implicated in infection are *Campylobacter jejuni* and *Campylobacter coli*, with a number of other emerging *Campylobacter* species such as *Campylobacter lari* and *Campylobacter fetus* increasingly identified, particularly in immunocompromized human hosts [3, 4]. Chronic infections and post-infectious sequelae such as Guillain-Barré syndrome are a risk for such individuals [5, 6].

Reported *Campylobacter* outbreaks are relatively rare, forming only 1% of the overall infection cases in Europe in 2020 [7]; the majority of *Campylobacter* cases are thus considered to be sporadic. The low proportion of reported outbreak cases likely relates to underreporting of human infection [8], and the complex and incompletely understood epidemiology of *Campylobacter.* Source attribution of cases can also be difficult; although many human *Campylobacter* infections derive from poultry meat [9], high proportions of cases have an unidentified source [10, 11]. The standard method of *Campylobacter* detection is through culture, though feasibility constraints usually result in the selection or characterization of as few as one isolate per sample [12, 13]. Previous studies have shown an increase in *Campylobacter* diversity between the farm and retail levels [14], likely resulting from cross-contamination, which increases the risk of foodborne illness for the consumer. Sampling a single isolate per sample can therefore lead to the underestimation of diversity of *Campylobacter* on epidemiologically relevant sources like chicken meat and infection-associated *Campylobacter* populations can be missed.

The culture method chosen for isolation is also important. Individual culture method approaches can be subject to isolation bias [15–17], as individual aspects of the method can result in preferential isolation of particular species or subtypes. For instance, *C. jejuni* and *C. coli* are thermophilic species, growing at both 37°C and 42°C, though other species may be excluded when culturing at 42°C only [17]. Although enrichment in selective broth can aid recovery of low abundance, sub-lethally injured cells, it can bias recovery of particular subtypes due to differing growth rates, competition for nutrients or preference for particular substrates [15, 16]. On the other hand, use of direct plating has been shown to be less effective for the detection of species like *C. coli* [18]. Different selective properties of the media used can also affect growth of competing bacteria, and therefore *Campylobacter* recovery. *Campylobacter* species are highly diverse, and techniques such as multi-locus sequence typing (MLST) have been useful for clustering of potential outbreak and source isolates based on sequence differences in seven housekeeping genes [19]. However, high variation of individual *Campylobacter* sequence types (STs) in other genomic regions indicates that whole genome sequencing (WGS) of multiple isolates per sample may be required for accurate source attribution [20].

Here, a combination of culture methods, including different broth, plate, and temperature conditions, were applied in order to maximize the isolation of *Campylobacter* from retail chicken meat, evaluate the diversity of the *Campylobacter* isolates obtained using WGS, and illustrate the potential implications of high intra-sample *Campylobacter* diversity on source attribution and outbreak investigation.

## Methods

### Sample collection

A total of 45 fresh pre-packaged retail chicken meat samples from chain stores in Norwich, Norfolk, UK were collected between March and November 2021 (Supplementary Table S1). The cuts sampled included chicken drumsticks, breasts, and thighs. Nine different stores (local, medium-sized, and large chain shops) were visited; care was taken to select different sample cuts and samples from different producers where possible, to reflect the variety of products available across the supply chain in the United Kingdom. Samples were stored at 2–8°C and processed within 72 h of receipt at Quadram Institute Bioscience, Norwich, UK.

### *Campylobacter* detection and isolation

Approximately 100 g of each sample was homogenized (Seward stomacher 400C laboratory blender, Worthing, UK) in 225 mL of buffered peptone water (BPW) (Southern Group Laboratory, Corby, UK) in FBAG-03 filter blender bags (Corning, New York, NY, USA). Two 35 mL aliquots of each homogenized sample were collected in 50 mL centrifuge tubes and subjected to centrifugation for 30 min at 4 000 rpm at 6°C (Eppendorf Centrifuge 5810 R, Stevenage, UK). After removing the supernatant, the pellets were resuspended in 5 mL of BPW, and the two replicate aliquots of each sample were pooled.

Multiple culture condition combinations were applied to each sample. This included three enrichment broth conditions (none – direct plating, Bolton broth, and cefoperazone, amphotericin B, teicoplanin (CAT) broth), two agar plate types (modified charcoal-cefoperazone-deoxycholate agar (mCCDA) and unsupplemented mCCDA (u-mCCDA)), and two temperatures (37°C and 42°C), resulting in 12 different culture method combinations. Details of the sample processing workflow are available in Supplementary Figure S1 and Supplementary Method 1.

At the end of the workflow, mCCDA and u-mCCDA plates were evaluated for typical growth, represented by small, grey to translucent colonies with an oily or metallic sheen. A maximum of four colonies were typically selected from each positive plate for the first 30 samples processed (CH-0312 to CH-0341), and a maximum of three colonies were selected from each positive plate for the last 15 samples (CH-0347 to CH-0361), resulting in a theoretical maximum of 48 or 36 isolates per sample, respectively, if all culture combinations yielded growth. The number of colonies selected was reduced from four to three after the first 30 samples due to feasibility constraints related to the quantity of isolates being recovered from the samples. For the first 30 samples, in cases where individual culture combinations did not display growth or fewer than four typical colonies were available, additional colonies were selected from other culture combinations, but the theoretical maximum was unchanged. For example, if for a particular sample Bolton broth enrichment at 37°C did not yield typical growth on mCCDA and u-mCCDA, additional colonies were selected from mCCDA and u-mCCDA following CAT broth enrichment at 37°C; or if u-mCCDA plates after CAT broth enrichment at 37°C did not yield growth, additional colonies were selected from the mCCDA plates after CAT broth enrichment at 37°C. Presumptive *Campylobacter* isolates were purified on Columbia blood agar (CBA) containing 5% horse blood (Trafalgar Scientific, Leicester, UK) and incubated in microaerophilic conditions (approximately 85% nitrogen, 10% carbon dioxide, and 5% oxygen) for 48 h at 37°C. Presumptive isolates were tested for oxidase production (Oxoid, Basingstoke, UK), and a subset of isolates was additionally examined under the microscope (Olympus CX41, Southend-on-Sea, UK) for typical *Campylobacter* morphology and motility (approximately 25% of the isolates, at least one per culture condition combination). Isolates were ultimately confirmed with sequencing. Those that could not be confirmed with sequencing were excluded from the dataset.

### DNA extraction and genome sequencing

DNA was extracted from each presumptive *Campylobacter* isolate using the Maxwell RSC Cultured Cells DNA kit (Promega, Southampton, UK). The Nextera Flex DNA library preparation kit was used for paired-end library preparation with either the Kapa 2G PCR kit (Merck) or NEB Q5 (New England Biolabs) polymerase kit used for library barcoding [21, 22]. The libraries were sequenced on an Illumina NextSeq (Illumina, Inc., San Diego, CA, USA) as 150 bp paired-end reads. Sequence data are deposited in the National Centre for Biotechnology Information (NCBI) Sequence Read Archive (SRA) (BioProject Accession No. PRJNA1022324); SRA accession numbers for individual genomes are available in Supplementary Table S2.

### Genomic analysis

Analyses were performed using Galaxy [23] and a QIB cloud server (adapted from the Cloud Infrastructure for Microbial Bioinformatics) [24]. The methods for read prefiltering, assembly, and quality control are detailed in Supplementary Method 2.

The genome species was classified using Centrifuge v1.0.4_beta [25], and AMR genes were identified using ABRicate v0.9.7 (https://github.com/tseemann/abricate) with the ResFinder database [26], using 90% identity and coverage thresholds. To identify quinolone and macrolide resistance determinants, custom databases were built in ARIBA v2.14.6 [27] comprised of *gyrA* genes extracted from the *C. jejuni* SAMEA1705929 (https://www.ncbi.nlm.nih.gov/biosample/SAMEA1705929/), *C. coli* SAMN11056450 (https://www.ncbi.nlm.nih.gov/biosample/SAMN11056450/), and *C. lari* SAMN02604025 (https://www.ncbi.nlm.nih.gov/biosample/SAMN02604025) reference genomes (for quinolone resistance mutations) and *C. jejuni* (NR_076226.1), *C. coli* (NR_121940.1), and *C. lari* (NR_076560.1) 23S rRNA genes (for macrolide resistance mutations). Details are outlined in Supplementary Method 3.

### Phylogenetic analysis

STs of genomes were determined with MLST v2.16.1 (https://github.com/tseemann/mlst) [19].

Sample diversity was further examined by grouping the isolates by species and analysing the trimmed reads with snippy and snippy-core v4.4.3 (https://github.com/tseemann/snippy), using the SAMEA1705929 (https://www.ncbi.nlm.nih.gov/biosample/SAMEA1705929/), SAMN02743854 (https://www.ncbi.nlm.nih.gov/biosample/SAMN02743854/), and SAMN02604025 (https://www.ncbi.nlm.nih.gov/biosample/SAMN02604025) reference genomes for *C. jejuni*, *C. coli*, and *C. lari*, respectively. Putative recombination regions were removed using Gubbins v2.4.1 [28]. The filtered polymorphic sites files obtained from Gubbins were used to construct maximum-likelihood phylogenetic trees using IQ-tree v1.6.12 in Galaxy (with 1 000 ultrafast bootstrap replicates) [29, 30]. To quantify non-recombinogenic pairwise SNPs within ST groups, the snippy-core full alignments were split by ST and separately analysed with Gubbins and snp-dists v0.6.3+galaxy0 (https://github.com/tseemann/snp-dists). For STs comprised of three genomes or less, the alignments were combined with alignments of a closely related ST to allow removal of putative recombination regions.

### Further investigation of STs isolated with individual methods

Five isolates representing four STs only isolated with either Bolton broth enrichment or CAT broth enrichment were selected for further study, in order to determine whether these STs could grow in all conditions or if their initial isolation was culture method dependent. The methods used closely followed those used for initial isolation, with full details available in Supplementary Method 4. The growth was enumerated to allow comparisons between conditions.

### Statistical analysis

A number of analyses were performed to determine whether or not there were significant differences in the recovery of *Campylobacter* between the different culture methods used for isolation. Since each sample under each temperature and broth condition was plated on both mCCDA and u-mCCDA, the effect of plate type on detection of any *Campylobacter* growth was tested using a McNemar test (for a paired binary outcome).

Mixed effects logistic regression modelling was then applied to test the effect of the temperature and broth predictor variables in a multivariable model, using data from mCCDA plates only in R v4.2.3 [31] with the glmmTMB v1.1.7 package [32], with the unique sample modelled as a random effect. As previously, the outcome variable modelled was *Campylobacter* growth or no growth. The main effects model was compared to a model with an interaction term between the broth and temperature conditions, using a log likelihood ratio test (Analysis of variance; ANOVA). Estimated marginal means for proportions along with asymptotic confidence intervals were computed from this model using the emmeans v1.8.5 package [33].

Finally, a Wilcoxon signed-rank test was used to compare log CFU/mL values obtained from the two plate types (mCCDA and CBA) used in the experiments comparing growth of isolates belonging to STs initially isolated with only one method (Bolton broth only or CAT broth only). In all cases, the *p*-value significance threshold was set to 0.05.

### Simulation studies

Simulation studies were conducted to understand the potential implications of selecting a small number of isolates per sample in the presence of *Campylobacter* ST diversity. A rarefaction curve without replacement was made with the vegan v2.6-4 R package to reflect the diversity of STs across samples [34]. A rarefaction curve was also generated by resampling with replacement of the observed ST distribution (Supplementary Table S3).

In each simulation, random subsamples of size *N* (with replacement) of the observed isolates were selected, with the estimated diversity from the subsample compared to the total observed diversity from all isolates. For simplicity, each simulation considered the observed ST distribution across culture conditions as representing the true distribution of STs within each sample.

First, the number and proportion of the different STs detected in each sample were measured as the number of isolates selected was increased. The number of isolates needed for the expected number of different STs found in the subsample to be at least 95% of the observed number in the whole sample was determined.

Second, one of the STs from each sample was randomly selected as being of interest – for example, as the causative agent in the context of an outbreak – and the probability of its detection was calculated as the number of sampled isolates increased. This was then averaged over each ST detected in each sample to determine the average number of isolates needed for the probability of detecting a specific ST to be at least 95%.

## Results

### Comparison of culture approaches for the isolation of *Campylobacter*

**Figure 1.**
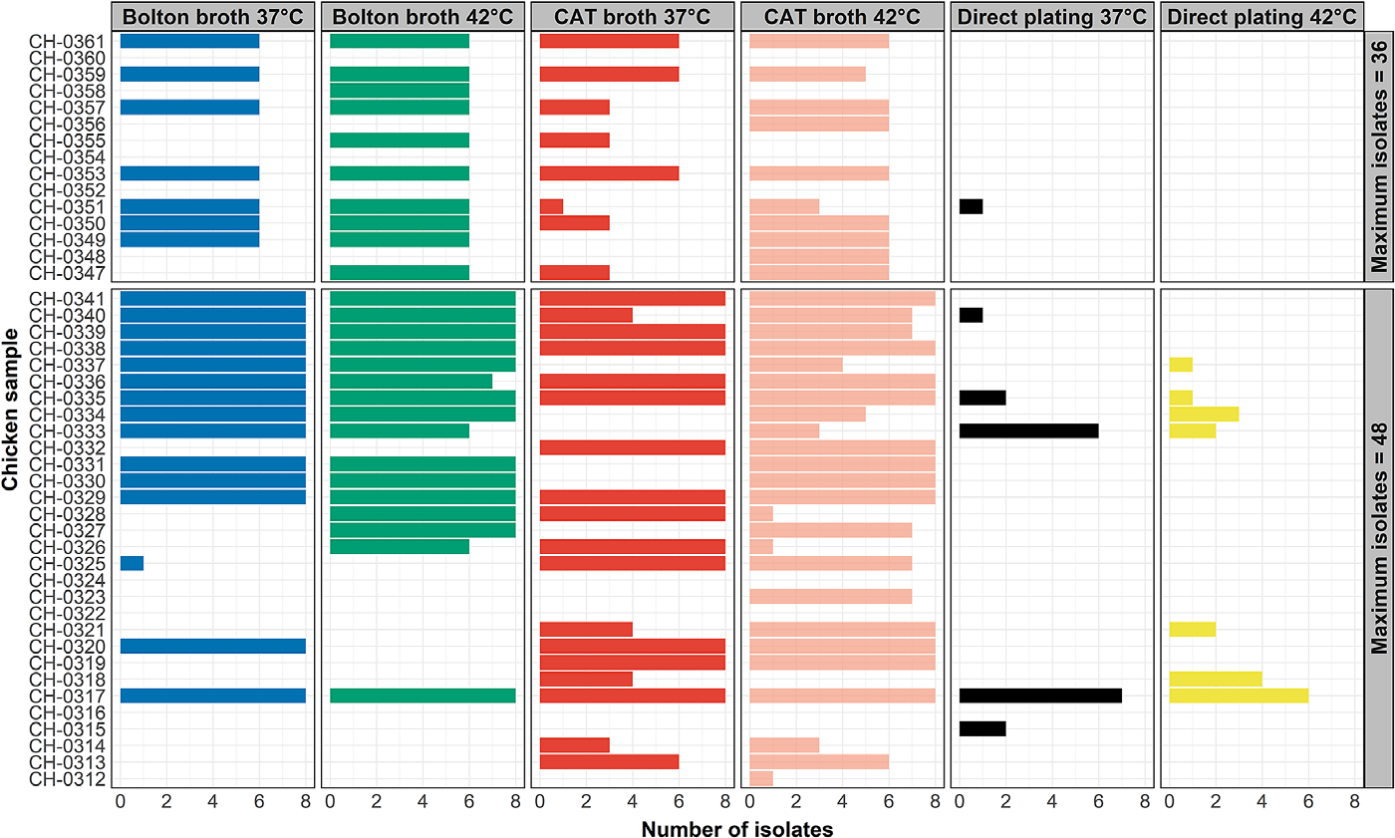
Number of *Campylobacter* isolates recovered from 45 retail chicken samples through direct plating (mCCDA and u-mCCDA) and enrichment in Bolton broth and CAT broth followed by plating on mCCDA and u-mCCDA at 37°C and 42°C, with the theoretical maximum number of isolates outlined.

Of the 45 chicken samples processed, 39 (86.7%) were culture-positive for *Campylobacter.* A total of 28 samples (62.2%) were positive for *Campylobacter* through Bolton broth enrichment, 37 (82.2%) through CAT broth enrichment, and 10 (22.2%) through direct plating. *Campylobacter* was recovered from 33 (73.3%) samples at 37°C and 38 (84.4%) at 42°C. Only two samples (CH-0317 and CH-0335) out of 39 were culture-positive through all broth and temperature combinations (Figure 1), and only one was positive through all broth, temperature, and plate combinations (CH-0317).

A summary table of *Campylobacter* growth (1) or no growth (0) by sample was produced (Supplementary Table S4) and used as input for statistical analysis.

**Figure 2.**
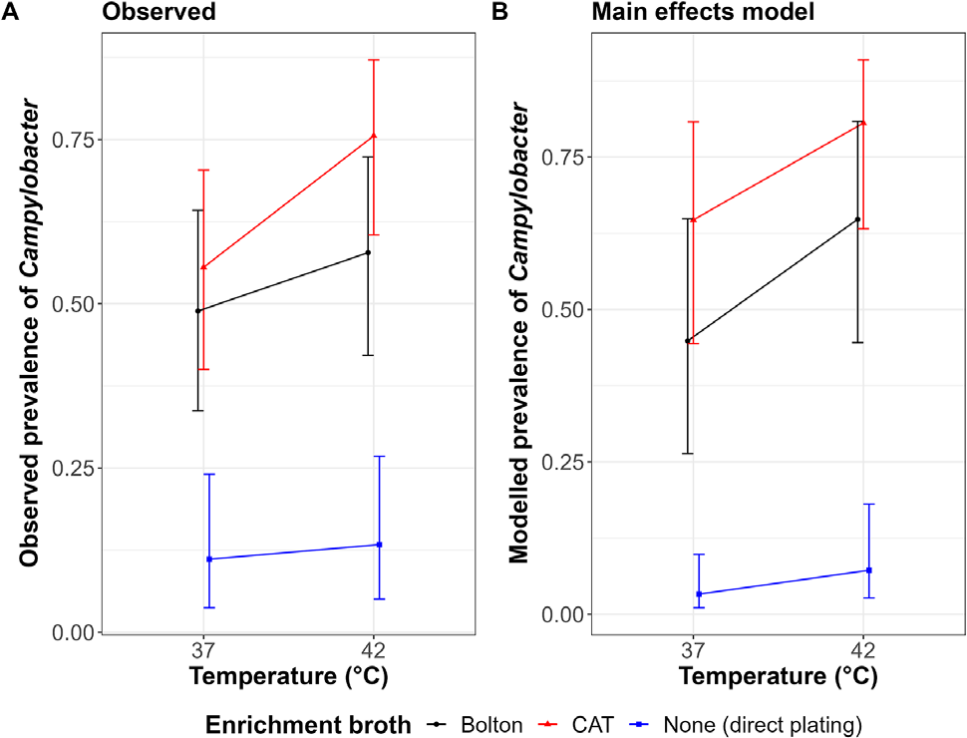
(A) The observed proportion of samples testing positive for *Campylobacter* under each condition on mCCDA plates. (B) The estimated marginal mean proportions of samples testing positive under each condition in the final model. Error bars represent 95% confidence intervals.

The concordance between plate types was high; in 94 cases, *Campylobacter* was detected using both plates, for 148 cases it was not detected on either. However, plating using mCCDA appeared more sensitive; in 24 cases, *Campylobacter* was detected using mCCDA but not with u-mCCDA, while the reverse was true in only four cases (McNemar test *p* = 0.0003; Supplementary Table S5A). As a result, multivariable mixed effects models to test the effect of broth and temperature were constructed using summarized *Campylobacter* presence/absence data from mCCDA plates only as the sensitive plating method. The final model showed significantly higher detection of *Campylobacter* at 42°C compared to 37°C (OR = 2.264; *p* = 0.02) and with CAT broth enrichment compared to Bolton broth (OR = 2.253; *p* = 0.039) or Bolton broth compared to direct plating (OR = 23.8; *p* < 0.001) (Figure 2). An interaction term between broth and temperature did not significantly improve the model (*p* = 0.39) (Supplementary Tables S5B and S5C), suggesting no evidence that the effect of temperature varied by broth.

### Intra-sample species and ST diversity and potential implications on source attribution

**Figure 3.**
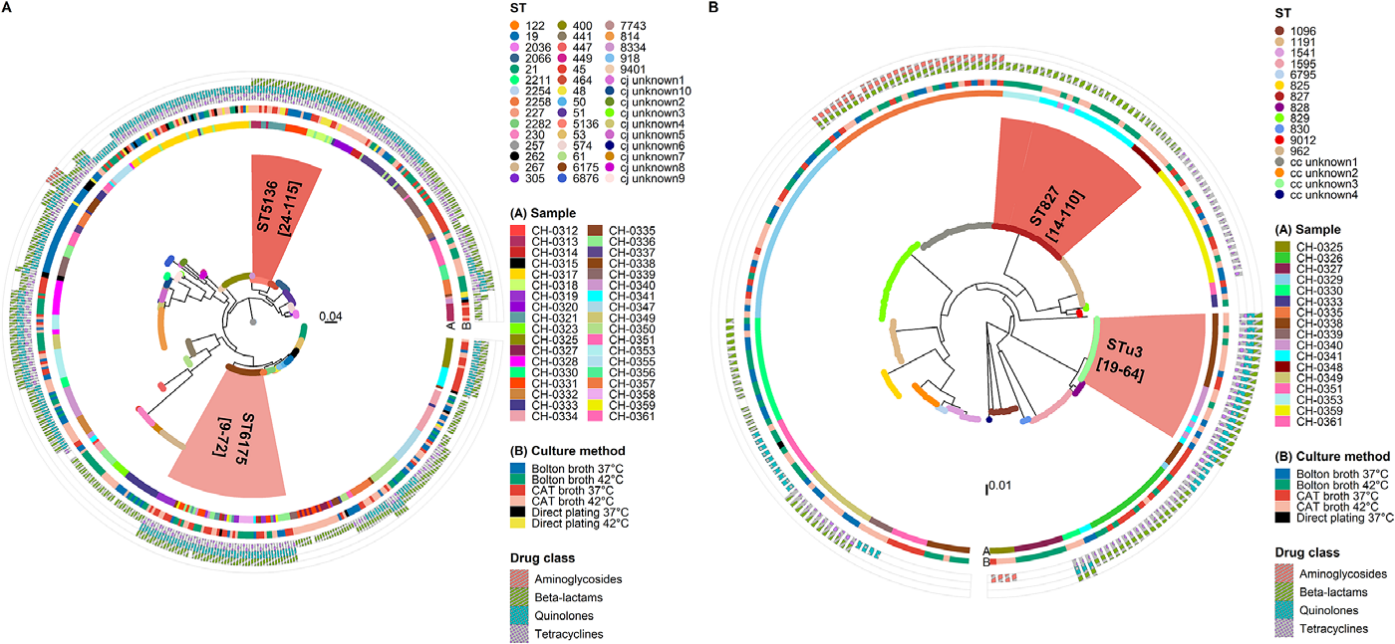
Maximum likelihood trees displaying the most common sequence types (STs) (ST-6175 and ST-5136 for *Campylobacter jejuni* and ST-827 and ST-unknown3 for *Campylobacter coli*) and their pairwise SNP difference ranges (in square brackets), the chicken sample of origin, the culture method for recovery, and the number of AMR determinants identified in the 499 *C. jejuni* genomes (a) and the 228 *C. coli* genomes (b).

Overall, 15 samples (33.3%) were positive for more than one *Campylobacter* species. *C. jejuni* was identified in 36 samples (80.0%) and *C. coli* in 17 samples (37.8%), with 14 samples (31.1%) containing both species. The phylogenies of the *C. jejuni* and *C. coli* isolates are displayed in Figure 3. One sample (CH-0320) contaminated with *C. jejuni* was also positive for *C. lari.* Of the 743 *Campylobacter* isolates recovered in this study, 499 (67.2%) were *C. jejuni*, 228 (30.7%) were *C. coli*, and 16 (2.2%) were *C. lari.* *C. jejuni* isolates were recovered through all culture method combinations tested, while *C. coli* isolates were recovered using Bolton broth and CAT broth at both temperatures, with only one isolate recovered with direct plating at 37°C on mCCDA. All 16 *C. lari* isolates were obtained through enrichment at 37°C, equally with CAT broth and Bolton broth.

**Figure 4.**
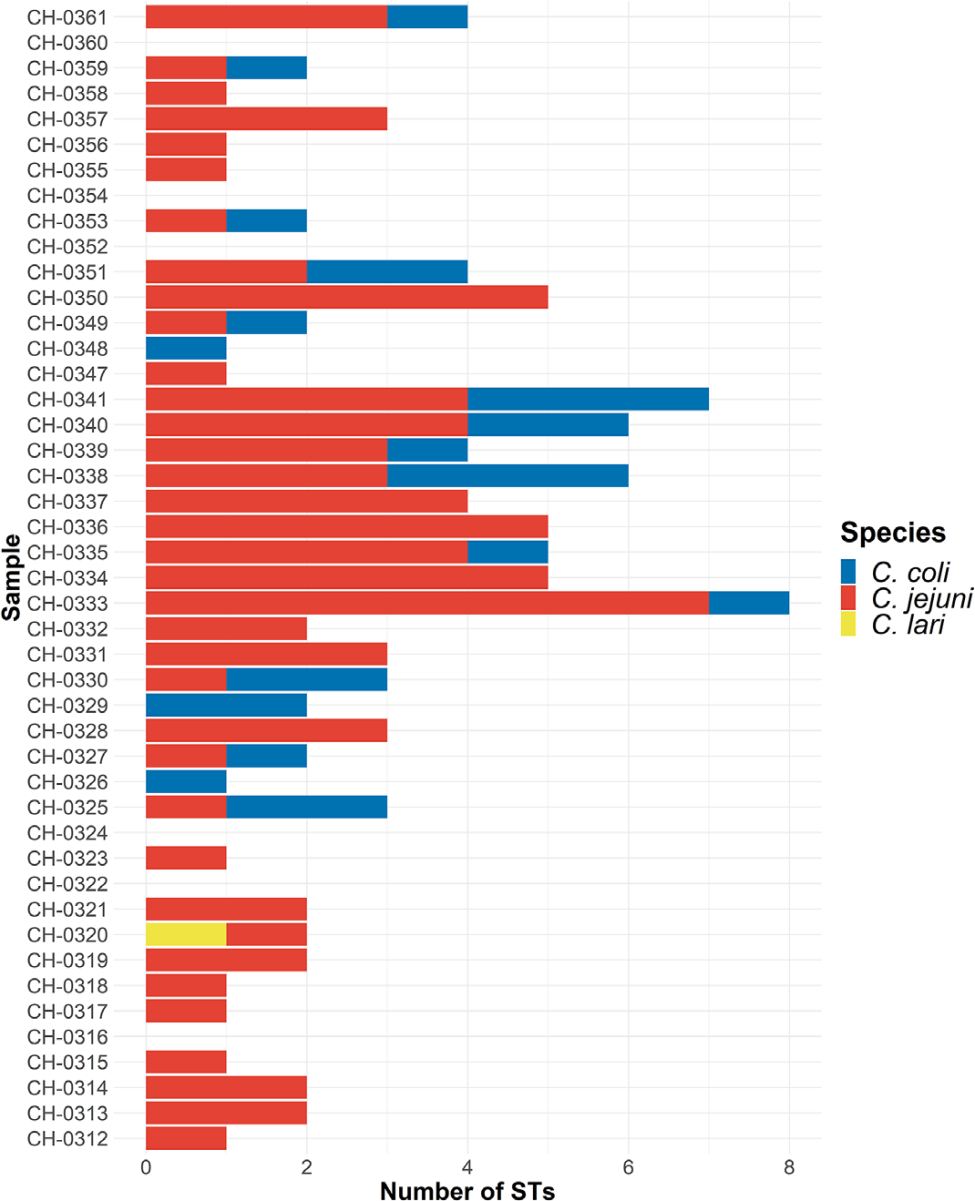
Number of *Campylobacter* sequence types (STs) identified in the retail chicken samples, coloured by species.

A total of 62 different STs were identified amongst the three *Campylobacter* species, 14 of which were novel STs (Supplementary Table S3). The most common *C. jejuni* STs identified were ST-6175 (11 samples) and ST-5136 (6 samples), whereas ST-827 (4 samples) and one of the novel STs (cc unknown3; 3 samples) were the most common *C. coli* STs. The number of STs found in a single culture positive sample ranged between 1 and 8 (Figure 4).

**Figure 5.**
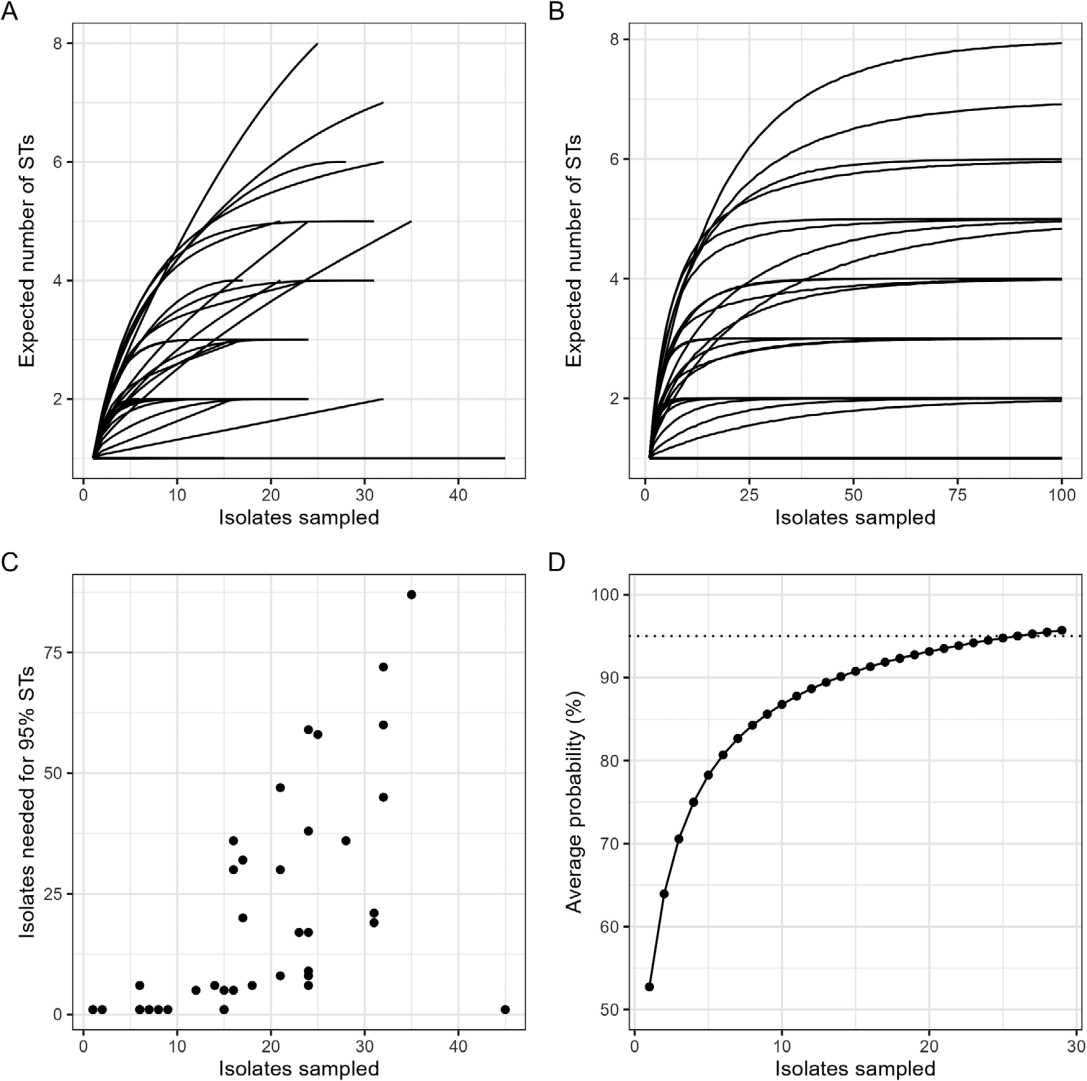
Sequence type (ST) rarefaction curve without replacement (A) and with replacement (B). Simulations performed followed the principle of rarefying with replacement to determine the number of isolates required to observe 95% of the ST diversity within each sample compared to the number of isolates that were collected (C) and to determine the average probability of detecting a randomly selected ST as the number of isolates is increased. The dashed line represents an average probability of 95% (D).

Rarefaction curves of STs for each sample (with and without replacement) demonstrated a wide range in the number of expected STs recovered with increased sampling intensity (Figure 5A,B). The number of isolates required to obtain 95% of the sample ST diversity for each sample ranged between one, if only a single ST in a sample was detected, to 87 (median = 8), when a high number of STs or very rare STs (represented by a low number of isolates within the sample) were identified (Figure 5C).

When randomly selecting each of the STs within individual samples as the ST of interest in a theoretical outbreak situation, the average probability of detecting the selected ST was 53% if a single isolate was sampled (Figure 5D). To achieve an average probability of 95% of detecting the selected ST amongst the sampled isolates, 26 isolates per sample were required (Figure 5D).

### Diversity of individual lineages inferred with pairwise SNP analysis

Fifty-five STs were represented by more than one isolate, allowing determination of pairwise SNP distances within STs. Within individual samples, the maximum SNP distances between isolates within STs ranged from 17 to 244 (median maximum = 54) SNPs, while overall, maximum SNP distances for STs varied between 22 and 2 413 (median maximum = 86) SNPs (Supplementary Figure S2). Only four STs within individual samples displayed minimum SNP distances of five SNPs or less, indicating closely related subsets of isolates. These were ST-21 in CH-0325 and CH-0341, ST-53 in CH-0334, ST-262 in CH-0347, and ST-19 in CH-0350.

The range of maximum SNP distances for *C. coli* STs within samples was 17–163 (median maximum = 53.5) and for *C. jejuni* 17–244 (median maximum = 54.5) (Supplementary Table S6). The highest within-sample SNP distance was observed for ST-51 in sample CH-0339 (maximum 244 SNPs). The *C. lari* ST-27 isolates from sample CH-0320 did not exceed 65 pairwise SNPs.

The range of the maximum SNP distances for *C. coli* STs overall was 46–2 413 (median maximum = 110), whereas the range of maximum SNP distances for *C. jejuni* STs overall was 22–244 (median maximum = 86) SNPs, similar to the within-sample range.

### Within-ST differences in antimicrobial resistance genotypes

The number of AMR determinants ranged from 0 to 4 per genome (Figure 3). *C. jejuni* and *C. coli* genomes displayed genotypes conferring resistance to beta-lactams (73.7%), aminoglycosides (5.4%), tetracyclines (53.9%), and quinolones (52.5%) (Supplementary Table S7). The *C. lari* genomes recovered from the same sample contained the beta-lactamase gene *bla*_OXA-493_ only. Multidrug resistant genotypes (indicating resistance to at least three different classes of antibiotics) were identified in both *C. jejuni* (33.5%) and *C. coli* (11.0%) genomes, from 23 and 4 samples, respectively.

Within-ST differences in AMR genotype were observed in eight STs within individual samples (Supplementary Tables S8 and S9).

### Further investigation of STs isolated using individual methods

There were 23 STs consisting of more than one isolate identified through direct plating or Bolton broth enrichment or CAT broth enrichment only. Five isolates representing four STs initially identified through either CAT or Bolton broth enrichment only were cultured from frozen stock and subjected to all of the 12 condition combinations used for initial isolation, with minor modifications (Supplementary Method 4). One isolate was removed from analysis due to swarming growth, as enumeration was not possible.

Despite initial recovery of the isolates with only one culture method, enumerable growth was observed in all conditions evaluated for all isolates (Supplementary Figure S3 and Supplementary Table S10). Growth varied between the conditions and the condition yielding the highest growth was not consistently the method that initially facilitated identification in the sample. There was also a significant difference in the log CFU/mL values obtained between mCCDA and CBA, determined using a Wilcoxon signed-rank test (*p* < 0.001).

## Discussion

This study aimed to maximize the recovery of *Campylobacter* from retail chicken meat using a combination of culture methods, assess the diversity of the isolates recovered, and determine the potential impacts this may have on source attribution and outbreak investigation.

A high proportion of samples were contaminated with *Campylobacter* (86.7%), with 33.3% positive for two *Campylobacter* species. A high intra-sample ST diversity was also identified (1–8 STs per sample), highlighting the importance of the recovery of multiple isolates per sample. High diversity of other pathogens on food samples has been previously reported in studies employing multiple isolate sampling or targeted screening. For example, retail prawn samples have been shown to harbour multiple serovars of *Salmonella enterica* [35] and multiple species and STs of *Vibrio* [36]. Another study found that breaded chicken samples can be contaminated with more than one *S. enterica* serovar, which can complicate outbreak investigations associated with the food source in absence of targeted screening for infection associated serovars [37]. Thus, sampling and isolation strategy may have a large effect on outbreak detection. Presence of multiple *Campylobacter* populations can be indicative of numerous contamination events throughout the meat processing chain, as other studies have identified a higher number of *Campylobacter* subtypes at retail compared to that observed on farm [14]. This can affect outbreak investigations, as reported by a study in Australia that compared *C. jejuni* from cases to isolates from chicken liver pâté, the epidemiologically linked food source. The isolates recovered from the liver pâté belonged to a different ST compared to the case isolates [38]; thus, in the absence of high-quality epidemiological data, the source of the outbreak may have been missed. Based on the data available in the current study and random resampling, it was estimated that the number of isolates required to identify at least 95% of the total STs in a sample ranged between 1 and 87 (median = 8). Samples with high ST diversity and presence of rare STs require more intensive sampling; this suggests that common protocols for *Campylobacter* sampling may underestimate the diversity of this pathogen in individual food samples due to a small number of isolates typically being taken. Furthermore, in a theoretical outbreak scenario characterized by a homogenous (single ST) *Campylobacter* causative agent, a policy of sampling up to 26 isolates per sample would be required for the average probability of detecting a potential outbreak causative ST in that sample to reach 95%. If only one isolate was sampled, the average probability of that isolate being the ST of interest is reduced to 53%. Importantly, it is likely that these are conservative estimates as the study was limited in the number of isolates per treatment condition, with the possibility that this caused an underestimation of the diversity in the samples.

STs within individual samples can also be diverse. In this study, the maximum SNP distances of STs within samples varied for *C. jejuni* (17–244) and *C. coli* (17–163). *C. jejuni* SNP acquisition over time varies between lineages, with some estimations showing accumulation of 2–8 SNPs per year [20]. The current study reports SNP distances exceeding the likely natural accumulation of SNPs over the course of a chicken’s lifetime, suggesting multiple potential contamination sources or contamination with a genetically diverse *Campylobacter* population. The genomes were also screened for AMR determinants, revealing beta-lactam (73.7%), aminoglycoside (5.4%), tetracycline (53.9%), and quinolone (52.5%) resistant genotypes amongst the *C. jejuni* and *C. coli* genomes, whereas all *C. lari* genomes displayed beta-lactam resistant genotypes. However, it is worth noting that the wildtype *gyrA* gene of *C. lari* ssp. *lari* may also confer intrinsic resistance to quinolones [39]. The proportion of *C. jejuni* and *C. coli* genomes with resistant genotypes was similar to the proportions of resistant *Campylobacter* from UK chicken samples reported by the Food Standards Agency [40], particularly in the case of quinolones and tetracyclines. The proportion of genomes with aminoglycoside resistant genotypes was slightly higher in this study, though this may be due to the collection of multiple isolates per sample, which can increase sensitivity of detection of resistant clones. Beta-lactam resistance is not often reported as *Campylobacter* are intrinsically resistant to many beta-lactams [41]. Eight STs displayed differences in AMR genotype within samples (Supplementary Table S9); for chromosomally located genes, AMR genotype differences can potentially represent the presence of multiple populations. This suggests that the number of isolates required to identify a particular lineage on a chicken sample may further exceed current estimates of up to 26 isolates per sample based on ST resampling. This highlights the importance of extensive sampling of isolates from food samples implicated in outbreaks; this is not often done, with many protocols isolating or characterizing one isolate per sample or only a subset of isolates obtained from the overall dataset [12, 13, 42]. This could be a potential reason why the majority of *Campylobacter* cases are considered to be sporadic [7]; if the source contains multiple strains, then cases exposed to the source may be infected with different strains that do not appear epidemiologically related.

The *Campylobacter* isolation method used can affect *Campylobacter* recovery from chicken samples, thus also potentially hindering source attribution and outbreak investigations. The widely used International Organization for Standardisation method for the isolation of *Campylobacter* from chicken meat recommends the use of Bolton broth as one of the enrichment media [43]; thus, this enrichment broth has been applied by many previous studies [44, 44]. However, this has been suggested to be inefficient in the recovery of *Campylobacter* from samples with low *Campylobacter* abundance and a high abundance of competing microbes [46]. Supplementation of Bolton broth with additional or alternative antimicrobial agents can result in enhanced *Campylobacter* recovery from retail meats [47]; this was also evidenced in this study through the use of CAT broth (Figure 1), which was associated with a higher *Campylobacter* detection rate compared to Bolton broth*. Campylobacter* detection was significantly lower with direct plating, which may be related to low abundance of the pathogen on the samples compared to other organisms. The culture method can therefore have implications for reported contamination rates, as false negative results may be obtained.

In the current study, *C. jejuni* was recovered with all tested method combinations, whereas *C. coli* was isolated mainly with enrichment; only one *C. coli* isolate was obtained with direct plating. Direct plating has been previously associated with reduced *C. coli* recovery compared to enrichment [18], suggesting that enrichment may be more efficient for *C. coli* identification. For samples containing both *C. jejuni* and *C. coli*, this may be related to the relative abundance of the species, whereby recovery of *C. coli* could be reduced if *C. coli* was less abundant than *C. jejuni.* It has also been suggested that the dominance of certain *Campylobacter* STs, such as ST-45, ST-50, and ST-21, in the PubMLST database and in certain studies may be in part a result of isolation bias introduced by culture methods [15], indicating that a combination of methods is optimal for understanding the true intra-sample diversity of *Campylobacter.* In this study, 23 STs consisting of more than one isolate were only recovered using one method: either direct plating, Bolton broth or CAT broth enrichment. However, it is important to note that a random selection of isolates was picked from the agar plates, and therefore the identification of certain STs through individual methods is likely to have occurred by chance. Evaluation of the growth of four isolates originally only isolated with Bolton or CAT broth revealed growth capability in all conditions, suggesting that isolation of these STs is possible in all the tested culture conditions. However, the preferential initial isolation with Bolton or CAT broth may be related to the presence of competing bacteria; different selective properties of the media used may have had different effects in repression of growth of non-target contaminants [46], leading to reduced initial isolation of certain STs.

To the best of our knowledge, this is the largest number of isolates recovered per sample of chicken meat to date, and the collection of multiple isolates per sample facilitated the identification of significant ST diversity as well as differences in SNP distances and AMR genotypes within STs. This has important implications on our understanding of intra-sample *Campylobacter* diversity, the inference of which is important for understanding the survival of this pathogen on chicken meat, potential risk for the consumer, outbreak investigation, and source attribution.

## Supporting information

Dziegiel et al. supplementary material Dziegiel et al. supplementary material

## Data Availability

Raw sequence data of the genomes analysed in this study are available in the National Centre for Biotechnology Information (NCBI) Sequence Read Archive (SRA) (BioProject Accession No. PRJNA1022324).

## Supplementary material

For supplementary material accompanying this paper visit http://doi.org/10.1017/S0950268824000906. click here to view supplementary material
