# Supplementary material for: High Campylobacter diversity in retail chicken: epidemiologically important strains may be missed with current sampling methods
Source: Epidemiol Infect. 2024 Aug 22;152:e101. doi: 10.1017/S0950268824000906 (PMC11736455; doi:10.1017/S0950268824000906)
Supplement: Dziegiel et al. supplementary material [file S0950268824000906sup001.pdf]

Epidemiology and Infection

High *Campylobacter* diversity in retail chicken – epidemiologically important strains may be missed with current sampling methods

Agata H Dziegiel, Samuel J Bloomfield, George M Savva, Raphaëlle Palau, Nicol Janecko, John Wain, Alison E Mather

Supplementary Materials

Contents

Table S1: Metadata associated with the chicken samples collected for the study 2

Figure S1: Sample processing workflow for *Campylobacter* isolation, showing the direct plating (mCCDA and u-mCCDA) and enrichment (Bolton and CAT broth) and subsequent plating (mCCDA and u-mCCDA) steps followed by isolation and purification on CBA; the lighter colour of the bottom CBA plates indicates a change in the number of colonies typically taken from each plate (four from the first 30 samples, three from the last 15 samples) 3

Supplementary Method 1: Sample processing and culturing procedure 4

Table S2: Accession information for the *Campylobacter* genomes generated in the study 4

Supplementary Method 2: Read prefiltering, assembly and quality control 24

Supplementary Method 3: Additional antimicrobial resistance determinant screening 24

Supplementary Method 4: Culturing procedure for isolates representing STs identified with Bolton broth enrichment or CAT broth enrichment only 24

Table S3: Summary of the *Campylobacter* genomes obtained in this study, the samples they were obtained from, the culture method they were isolated with and their ST 25

Table S4: Summary table of *Campylobacter* growth (1) or no growth (0) from each culture condition combination for each sample, used as the input for mixed effects logistic regression models 40

Table S5A: Comparison tables for the agar plate types used for *Campylobacter* recovery 51

Table S5B: Comparison of the main effects logistic regression model using mCCDA data only (mCCDA_main) and a logistic regression model with an interaction term between Broth and Temperature (mCCDA_interaction), with the chicken sample modelled as a random effect; the values in the table represent odds ratios, confidence intervals and p-values 51

Table S5C: Model comparisons using ANOVA 51

Figure S2: Highest non-recombinogenic pairwise SNP difference between isolates of individual STs* within individual chicken samples (A) and overall (B), with STs displaying differences of >200 SNPs annotated 52

Table S6: Pairwise non-recombinogenic SNP distances for *Campylobacter* STs identified in the study within samples and overall 53

Table S7: Antimicrobial resistance determinants identified in the genomes of *Campylobacter jejuni*, *C. coli* and *C. lari* isolates recovered 56

Table S8: *Campylobacter* ST groups of isolates recovered from individual chicken samples and their AMR genotypes 57

Table S9: Intra-ST differences in AMR genotype within samples 61

Figure S3: Comparison of growth (log CFU/mL) in different conditions tested in the study based on plate counts on CBA and mCCDA of four isolates representing four different STs previously identified through one condition (Bolton broth/CAT broth) only 62

Table S10: Average plate counts and CFU/mL values for comparisons of growth of four isolates representing four STs previously identified through one condition only (Bolton broth/CAT broth) 63

Table S1: Metadata associated with the chicken samples collected for the study

Sample	Date Collected	Collection City	Commodity	Sample cut	Country of Origin
CH-0312	14/03/2021	Norwich	Chicken	Chicken drumstick (skin on)	United Kingdom
CH-0313	14/03/2021	Norwich	Chicken	Chicken breast (boneless, skinless)	United Kingdom
CH-0314	14/03/2021	Norwich	Chicken	Chicken breast (boneless, skinless)	United Kingdom
CH-0315	14/03/2021	Norwich	Chicken	Chicken thigh (skinless)	United Kingdom
CH-0316	14/03/2021	Norwich	Chicken	Chicken breast (boneless, skinless)	United Kingdom
CH-0317	10/04/2021	Norwich	Chicken	Chicken thigh (skinless, boneless)	United Kingdom
CH-0318	10/04/2021	Norwich	Chicken	Chicken drumstick (skin on)	United Kingdom
CH-0319	11/04/2021	Norwich	Chicken	Chicken thigh (skinless, boneless)	United Kingdom
CH-0320	11/04/2021	Norwich	Chicken	Chicken breast (boneless, skinless)	United Kingdom
CH-0321	11/04/2021	Norwich	Chicken	Chicken breast (boneless, skinless)	United Kingdom
CH-0322	08/05/2021	Norwich	Chicken	Chicken breast (boneless, skinless)	United Kingdom
CH-0323	08/05/2021	Norwich	Chicken	Chicken thigh (skinless, boneless)	United Kingdom
CH-0324	08/05/2021	Norwich	Chicken	Chicken drumstick (skin on)	United Kingdom
CH-0325	08/05/2021	Norwich	Chicken	Chicken breast (boneless, skinless)	United Kingdom
CH-0326	08/05/2021	Norwich	Chicken	Chicken thigh (skin on)	United Kingdom
CH-0327	05/06/2021	Norwich	Chicken	Chicken breast (boneless, skinless)	United Kingdom
CH-0328	05/06/2021	Norwich	Chicken	Chicken thigh (skinless, boneless)	United Kingdom
CH-0329	05/06/2021	Norwich	Chicken	Chicken thigh (skinless, boneless)	United Kingdom
CH-0330	05/06/2021	Norwich	Chicken	Chicken breast (boneless, skinless)	United Kingdom
CH-0331	05/06/2021	Norwich	Chicken	Chicken drumstick (skin on)	United Kingdom
CH-0332	19/06/2021	Norwich	Chicken	Chicken breast (boneless, skinless)	United Kingdom
CH-0333	19/06/2021	Norwich	Chicken	Chicken drumstick (skin on)	United Kingdom
CH-0334	19/06/2021	Norwich	Chicken	Chicken thigh (skin on)	United Kingdom
CH-0335	19/06/2021	Norwich	Chicken	Chicken breast (boneless, skinless)	United Kingdom
CH-0336	19/06/2021	Norwich	Chicken	Chicken drumstick (skin on)	United Kingdom
CH-0337	13/08/2021	Norwich	Chicken	Chicken thigh (skin on)	United Kingdom
CH-0338	13/08/2021	Norwich	Chicken	Chicken breast (boneless, skinless)	United Kingdom
CH-0339	14/08/2021	Norwich	Chicken	Chicken thigh (skinless, boneless)	United Kingdom
CH-0340	14/08/2021	Norwich	Chicken	Chicken breast (boneless, skinless)	United Kingdom
CH-0341	14/08/2021	Norwich	Chicken	Chicken drumstick (skin on)	United Kingdom
CH-0347	14/10/2021	Norwich	Chicken	Chicken breast (boneless, skinless)	United Kingdom
CH-0348	14/10/2021	Norwich	Chicken	Chicken drumstick (skin on)	United Kingdom
CH-0349	14/10/2021	Norwich	Chicken	Chicken breast (boneless, skinless)	United Kingdom
CH-0350	15/10/2021	Norwich	Chicken	Chicken breast (boneless, skin on)	United Kingdom
CH-0351	15/10/2021	Norwich	Chicken	Chicken thigh (skinless, boneless)	United Kingdom
CH-0352	04/11/2021	Norwich	Chicken	Chicken thigh (skinless, boneless)	United Kingdom
CH-0353	04/11/2021	Norwich	Chicken	Chicken breast (boneless, skinless)	United Kingdom
CH-0354	04/11/2021	Norwich	Chicken	Chicken breast (boneless, skinless)	United Kingdom
CH-0355	04/11/2021	Norwich	Chicken	Chicken breast (boneless, skinless)	United Kingdom
CH-0356	05/11/2021	Norwich	Chicken	Chicken thigh (skin on)	Poland
CH-0357	26/11/2021	Norwich	Chicken	Chicken thigh (skin on)	Poland
CH-0358	26/11/2021	Norwich	Chicken	Chicken breast (boneless, skinless)	Poland
CH-0359	26/11/2021	Norwich	Chicken	Chicken breast (boneless, skinless)	United Kingdom
CH-0360	26/11/2021	Norwich	Chicken	Chicken drumstick (skin on)	United Kingdom
CH-0361	26/11/2021	Norwich	Chicken	Chicken breast (boneless, skinless)	United Kingdom

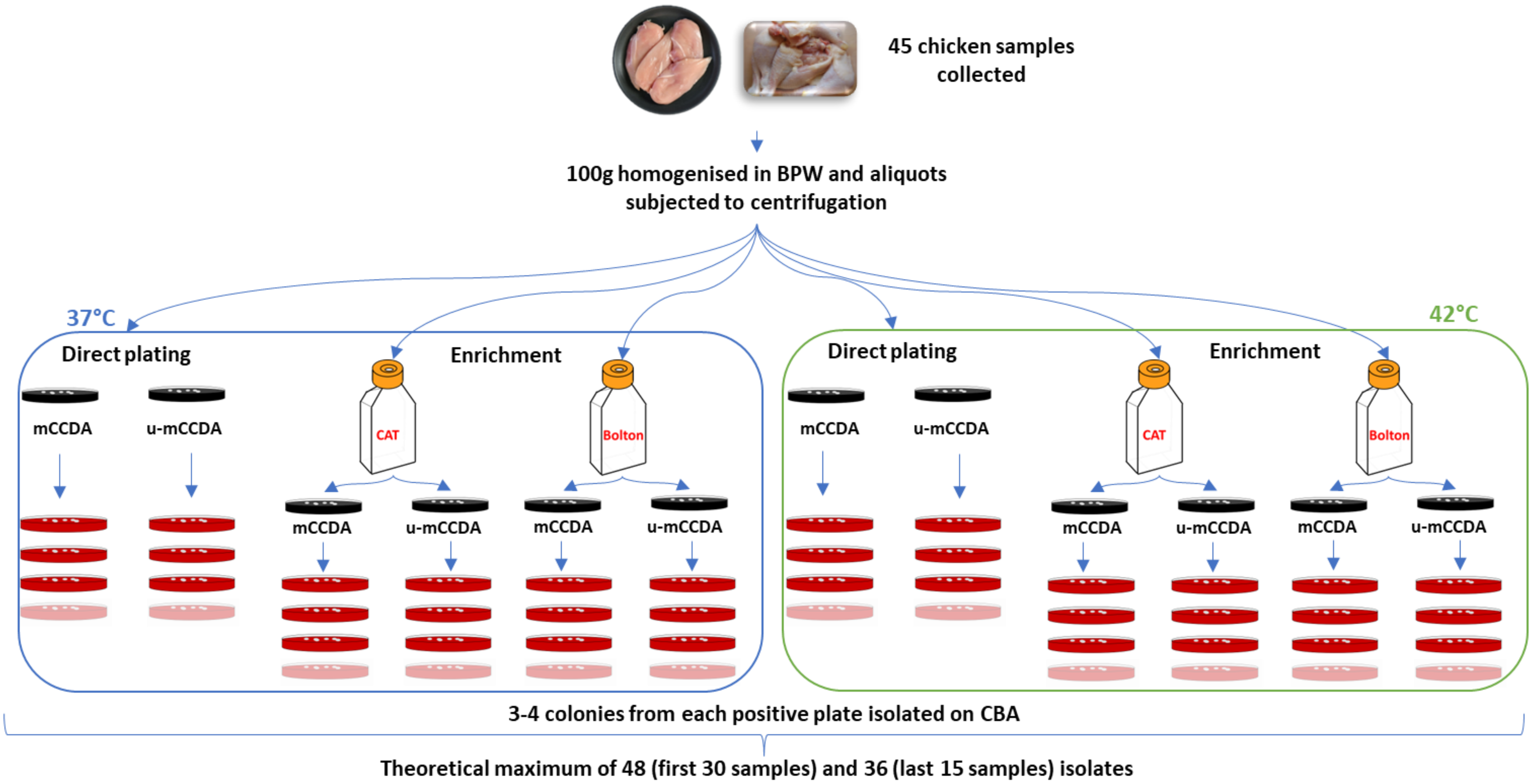

Figure S1: Sample processing workflow for *Campylobacter* isolation, showing the direct plating (mCCDA and u-mCCDA) and enrichment (Bolton and CAT broth) and subsequent plating (mCCDA and u-mCCDA) steps followed by isolation and purification on CBA; the lighter colour of the bottom CBA plates indicates a change in the number of colonies typically taken from each plate (four from the first 30 samples, three from the last 15 samples)

CAT = cefoperazone, amphotericin B, teicoplanin broth; CBA = Columbia blood agar; mCCDA = modified charcoal-cefoperazone-deoxycholate agar; u-mCCDA = unsupplemented modified charcoal-cefoperazone-deoxycholate agar

Supplementary Method 1: Sample processing and culturing procedure

Method A: Direct plating

Using a sterile inoculation loop, 10 µL of the centrifuged pooled sample was streaked on modified charcoal-cefoperazone-deoxycholate agar (mCCDA) plates (Oxoid, Basingstoke, UK) containing cefoperazone and amphotericin B antimicrobials, and mCCDA plates without antimicrobial supplements (u-mCCDA) in duplicate. One plate of each type was incubated for 48 hours at 37°C in microaerophilic conditions (85% nitrogen, 10% carbon dioxide and 5% oxygen) in an anaerobic cabinet (Don Whitley Scientific MAC 1000 Microaerophilic Workstation, Bingley, UK) and the remaining plates at 42°C in microaerophilic conditions using Anoxomat jars (Mart Microbiology B.V Anoxomat System AN2CTS, Drachtstercompagnie, Netherlands) filled with a gas mixture comprised of approximately 85% nitrogen, 10% carbon dioxide and 5% oxygen.

Method B: Enrichment in Bolton broth

For the first stage of the study (featuring the first 15 samples), two sterile universal bottles filled with 20 mL of Bolton broth (Oxoid) were each inoculated with 1 mL of the centrifuged pooled sample. One universal bottle was incubated at 37°C and the other at 42°C for 48 hours in microaerophilic conditions, as previously, with caps loosened. For the remaining 30 samples, sterile tissue flasks (Geiner Bio-One, Stonehouse, UK) were used instead of sterile universal bottles, reflecting improvement to the method after the pilot stage of the study. Use of tissue flasks instead of universal bottles reduces the risk of spillages due to loosened caps, as tissue flasks have filtered lids that allow airflow. Following enrichment, 10 µL of each enrichment was streaked on mCCDA and u-mCCDA and subjected to the same temperature as the enrichment broth for 44-48 hours in microaerophilic conditions.

Method C: Enrichment in CAT broth

For the first 15 samples, two sterile universal bottles filled with 20 mL of cefoperazone, amphotericin B, teicoplanin (CAT) broth (Oxoid) were each inoculated with 1 mL of the centrifuged pooled sample. For the remaining 30 samples, sterile tissue flasks were used instead of sterile universal bottles. CAT broth was prepared the same way as Bolton broth, except the selective supplement was substituted for CAT supplement (Oxoid). The broths were incubated and processed as described in Method B.

Table S2: Accession information for the *Campylobacter* genomes generated in the study

Sample	Genome	SRA accession	Biosample	Bioproject	Organism	Isolation source
CA21CH-0312-6-1	CA21CH-0312-6-1	SRR26215497	SAMN37598467	PRJNA1022324	*Campylobacter jejuni*	Retail chicken
CA19CH-0313-3-1	CA19CH-0313-3-1	SRR26215496	SAMN37598468	PRJNA1022324	*Campylobacter jejuni*	Retail chicken
CA21CH-0313-3-2	CA21CH-0313-3-2	SRR26215193	SAMN37598469	PRJNA1022324	*Campylobacter jejuni*	Retail chicken
CA21CH-0313-3-3	CA21CH-0313-3-3	SRR26215043	SAMN37598470	PRJNA1022324	*Campylobacter jejuni*	Retail chicken
CA21CH-0313-3-4	CA21CH-0313-3-4	SRR26214932	SAMN37598471	PRJNA1022324	*Campylobacter jejuni*	Retail chicken
CA21CH-0313-3-5	CA21CH-0313-3-5	SRR26214757	SAMN37598472	PRJNA1022324	*Campylobacter jejuni*	Retail chicken
CA21CH-0313-3-6	CA21CH-0313-3-6	SRR26215293	SAMN37598473	PRJNA1022324	*Campylobacter jejuni*	Retail chicken
CA21CH-0313-6-1	CA21CH-0313-6-1	SRR26215246	SAMN37598474	PRJNA1022324	*Campylobacter jejuni*	Retail chicken
CA21CH-0313-6-2	CA21CH-0313-6-2	SRR26215224	SAMN37598475	PRJNA1022324	*Campylobacter jejuni*	Retail chicken
CA21CH-0313-6-3	CA21CH-0313-6-3	SRR26215213	SAMN37598476	PRJNA1022324	*Campylobacter jejuni*	Retail chicken
CA21CH-0313-6-4	CA21CH-0313-6-4	SRR26215495	SAMN37598477	PRJNA1022324	*Campylobacter jejuni*	Retail chicken
CA21CH-0313-6-5	CA21CH-0313-6-5	SRR26215484	SAMN37598478	PRJNA1022324	*Campylobacter jejuni*	Retail chicken
CA21CH-0313-6-6	CA21CH-0313-6-6	SRR26215473	SAMN37598479	PRJNA1022324	*Campylobacter jejuni*	Retail chicken
CA21CH-0314-3-2	CA21CH-0314-3-2	SRR26215462	SAMN37598480	PRJNA1022324	*Campylobacter jejuni*	Retail chicken
CA21CH-0314-3-3	CA21CH-0314-3-3	SRR26215451	SAMN37598481	PRJNA1022324	*Campylobacter jejuni*	Retail chicken
CA21CH-0314-3-4	CA21CH-0314-3-4	SRR26215440	SAMN37598482	PRJNA1022324	*Campylobacter jejuni*	Retail chicken
CA21CH-0314-6-1	CA21CH-0314-6-1R2	SRR26215429	SAMN37598483	PRJNA1022324	*Campylobacter jejuni*	Retail chicken

CA21CH-0314-6-2	CA21CH-0314-6-2R2	SRR26215418	SAMN37598484	PRJNA1022324	*Campylobacter jejuni*	Retail chicken
CA21CH-0314-6-3	CA21CH-0314-6-3R2	SRR26215407	SAMN37598485	PRJNA1022324	*Campylobacter jejuni*	Retail chicken
CA21CH-0315-1-1	CA21CH-0315-1-1	SRR26215204	SAMN37598486	PRJNA1022324	*Campylobacter jejuni*	Retail chicken
CA21CH-0315-1-2	CA21CH-0315-1-2-R2	SRR26215192	SAMN37598487	PRJNA1022324	*Campylobacter jejuni*	Retail chicken
CA21CH-0317-1-1	CA21CH-0317-1-1	SRR26215181	SAMN37598488	PRJNA1022324	*Campylobacter jejuni*	Retail chicken
CA21CH-0317-1-2	CA21CH-0317-1-2	SRR26215394	SAMN37598489	PRJNA1022324	*Campylobacter jejuni*	Retail chicken
CA21CH-0317-1-3	CA21CH-0317-1-3	SRR26215383	SAMN37598490	PRJNA1022324	*Campylobacter jejuni*	Retail chicken
CA21CH-0317-1-4	CA21CH-0317-1-4	SRR26215372	SAMN37598491	PRJNA1022324	*Campylobacter jejuni*	Retail chicken
CA21CH-0317-1-5	CA21CH-0317-1-5	SRR26215169	SAMN37598492	PRJNA1022324	*Campylobacter jejuni*	Retail chicken
CA21CH-0317-1-6	CA21CH-0317-1-6	SRR26215087	SAMN37598493	PRJNA1022324	*Campylobacter jejuni*	Retail chicken
CA21CH-0317-1-7	CA21CH-0317-1-7	SRR26215076	SAMN37598494	PRJNA1022324	*Campylobacter jejuni*	Retail chicken
CA21CH-0317-2-1	CA21CH-0317-2-1-R	SRR26215065	SAMN37598495	PRJNA1022324	*Campylobacter jejuni*	Retail chicken
CA21CH-0317-2-2	CA21CH-0317-2-2-R	SRR26215054	SAMN37598496	PRJNA1022324	*Campylobacter jejuni*	Retail chicken
CA21CH-0317-2-3	CA21CH-0317-2-3	SRR26215369	SAMN37598497	PRJNA1022324	*Campylobacter jejuni*	Retail chicken
CA21CH-0317-2-4	CA21CH-0317-2-4	SRR26215358	SAMN37598498	PRJNA1022324	*Campylobacter jejuni*	Retail chicken
CA21CH-0317-2-5	CA21CH-0317-2-5	SRR26215347	SAMN37598499	PRJNA1022324	*Campylobacter jejuni*	Retail chicken
CA21CH-0317-2-6	CA21CH-0317-2-6-R	SRR26214977	SAMN37598500	PRJNA1022324	*Campylobacter jejuni*	Retail chicken
CA21CH-0317-2-7	CA21CH-0317-2-7-R	SRR26214966	SAMN37598501	PRJNA1022324	*Campylobacter jejuni*	Retail chicken
CA21CH-0317-2-8	CA21CH-0317-2-8	SRR26214955	SAMN37598502	PRJNA1022324	*Campylobacter jejuni*	Retail chicken
CA21CH-0317-3-1	CA21CH-0317-3-1	SRR26215335	SAMN37598503	PRJNA1022324	*Campylobacter jejuni*	Retail chicken
CA21CH-0317-3-2	CA21CH-0317-3-2	SRR26215324	SAMN37598504	PRJNA1022324	*Campylobacter jejuni*	Retail chicken
CA21CH-0317-3-3	CA21CH-0317-3-3	SRR26215313	SAMN37598505	PRJNA1022324	*Campylobacter jejuni*	Retail chicken
CA21CH-0317-3-4	CA21CH-0317-3-4	SRR26214943	SAMN37598506	PRJNA1022324	*Campylobacter jejuni*	Retail chicken
CA21CH-0317-3-5	CA21CH-0317-3-5	SRR26214931	SAMN37598507	PRJNA1022324	*Campylobacter jejuni*	Retail chicken
CA21CH-0317-3-6	CA21CH-0317-3-6	SRR26214920	SAMN37598508	PRJNA1022324	*Campylobacter jejuni*	Retail chicken
CA21CH-0317-3-7	CA21CH-0317-3-7	SRR26214813	SAMN37598509	PRJNA1022324	*Campylobacter jejuni*	Retail chicken
CA21CH-0317-3-8	CA21CH-0317-3-8	SRR26214802	SAMN37598510	PRJNA1022324	*Campylobacter jejuni*	Retail chicken
CA21CH-0317-4-1	CA21CH-0317-4-1	SRR26214791	SAMN37598511	PRJNA1022324	*Campylobacter jejuni*	Retail chicken
CA21CH-0317-4-2	CA21CH-0317-4-2	SRR26214908	SAMN37598512	PRJNA1022324	*Campylobacter jejuni*	Retail chicken
CA21CH-0317-4-3	CA21CH-0317-4-3	SRR26214897	SAMN37598513	PRJNA1022324	*Campylobacter jejuni*	Retail chicken
CA21CH-0317-4-4	CA21CH-0317-4-4	SRR26214886	SAMN37598514	PRJNA1022324	*Campylobacter jejuni*	Retail chicken
CA21CH-0317-4-5	CA21CH-0317-4-5	SRR26214779	SAMN37598515	PRJNA1022324	*Campylobacter jejuni*	Retail chicken
CA21CH-0317-4-6	CA21CH-0317-4-6	SRR26214768	SAMN37598516	PRJNA1022324	*Campylobacter jejuni*	Retail chicken
CA21CH-0317-5-1	CA21CH-0317-5-1	SRR26214756	SAMN37598517	PRJNA1022324	*Campylobacter jejuni*	Retail chicken
CA21CH-0317-5-2	CA21CH-0317-5-2	SRR26214873	SAMN37598518	PRJNA1022324	*Campylobacter jejuni*	Retail chicken
CA21CH-0317-5-3	CA21CH-0317-5-3	SRR26214862	SAMN37598519	PRJNA1022324	*Campylobacter jejuni*	Retail chicken
CA21CH-0317-5-4	CA21CH-0317-5-4	SRR26214851	SAMN37598520	PRJNA1022324	*Campylobacter jejuni*	Retail chicken
CA21CH-0317-5-5	CA21CH-0317-5-5	SRR26215151	SAMN37598521	PRJNA1022324	*Campylobacter jejuni*	Retail chicken
CA21CH-0317-5-6	CA21CH-0317-5-6	SRR26215140	SAMN37598522	PRJNA1022324	*Campylobacter jejuni*	Retail chicken
CA21CH-0317-5-7	CA21CH-0317-5-7	SRR26215042	SAMN37598523	PRJNA1022324	*Campylobacter jejuni*	Retail chicken

CA21CH-0317-5-8	CA21CH-0317-5-8	SRR26215031	SAMN37598524	PRJNA1022324	*Campylobacter jejuni*	Retail chicken
CA21CH-0317-6-1	CA21CH-0317-6-1	SRR26215020	SAMN37598525	PRJNA1022324	*Campylobacter jejuni*	Retail chicken
CA21CH-0317-6-2	CA21CH-0317-6-2	SRR26215304	SAMN37598526	PRJNA1022324	*Campylobacter jejuni*	Retail chicken
CA21CH-0317-6-3	CA21CH-0317-6-3	SRR26215292	SAMN37598527	PRJNA1022324	*Campylobacter jejuni*	Retail chicken
CA21CH-0317-6-4	CA21CH-0317-6-4	SRR26215281	SAMN37598528	PRJNA1022324	*Campylobacter jejuni*	Retail chicken
CA21CH-0317-6-5	CA21CH-0317-6-5	SRR26214847	SAMN37598529	PRJNA1022324	*Campylobacter jejuni*	Retail chicken
CA21CH-0317-6-6	CA21CH-0317-6-6	SRR26214836	SAMN37598530	PRJNA1022324	*Campylobacter jejuni*	Retail chicken
CA21CH-0317-6-7	CA21CH-0317-6-7	SRR26214825	SAMN37598531	PRJNA1022324	*Campylobacter jejuni*	Retail chicken
CA21CH-0317-6-8	CA21CH-0317-6-8	SRR26215006	SAMN37598532	PRJNA1022324	*Campylobacter jejuni*	Retail chicken
CA21CH-0318-3-1	CA21CH-0318-3-1	SRR26214995	SAMN37598533	PRJNA1022324	*Campylobacter jejuni*	Retail chicken
CA21CH-0318-3-2	CA21CH-0318-3-2	SRR26214984	SAMN37598534	PRJNA1022324	*Campylobacter jejuni*	Retail chicken
CA21CH-0318-3-3	CA21CH-0318-3-3	SRR26215268	SAMN37598535	PRJNA1022324	*Campylobacter jejuni*	Retail chicken
CA21CH-0318-3-4	CA21CH-0318-3-4	SRR26215257	SAMN37598536	PRJNA1022324	*Campylobacter jejuni*	Retail chicken
CA21CH-0318-4-1	CA21CH-0318-4-1	SRR26215245	SAMN37598537	PRJNA1022324	*Campylobacter jejuni*	Retail chicken
CA21CH-0318-4-2	CA21CH-0318-4-2	SRR26215122	SAMN37598538	PRJNA1022324	*Campylobacter jejuni*	Retail chicken
CA21CH-0318-4-3	CA21CH-0318-4-3	SRR26215111	SAMN37598539	PRJNA1022324	*Campylobacter jejuni*	Retail chicken
CA21CH-0318-4-4	CA21CH-0318-4-4	SRR26215100	SAMN37598540	PRJNA1022324	*Campylobacter jejuni*	Retail chicken
CA21CH-0319-3-1	CA21CH-0319-3-1	SRR26215233	SAMN37598541	PRJNA1022324	*Campylobacter jejuni*	Retail chicken
CA21CH-0319-3-2	CA21CH-0319-3-2	SRR26215229	SAMN37598542	PRJNA1022324	*Campylobacter jejuni*	Retail chicken
CA21CH-0319-3-3	CA21CH-0319-3-3	SRR26215228	SAMN37598543	PRJNA1022324	*Campylobacter jejuni*	Retail chicken
CA21CH-0319-3-4	CA21CH-0319-3-4	SRR26215227	SAMN37598544	PRJNA1022324	*Campylobacter jejuni*	Retail chicken
CA21CH-0319-3-5	CA21CH-0319-3-5	SRR26215226	SAMN37598545	PRJNA1022324	*Campylobacter jejuni*	Retail chicken
CA21CH-0319-3-6	CA21CH-0319-3-6-R	SRR26215225	SAMN37598546	PRJNA1022324	*Campylobacter jejuni*	Retail chicken
CA21CH-0319-3-7	CA21CH-0319-3-7-R	SRR26215223	SAMN37598547	PRJNA1022324	*Campylobacter jejuni*	Retail chicken
CA21CH-0319-3-8	CA21CH-0319-3-8	SRR26215222	SAMN37598548	PRJNA1022324	*Campylobacter jejuni*	Retail chicken
CA21CH-0319-6-1	CA21CH-0319-6-1	SRR26215221	SAMN37598549	PRJNA1022324	*Campylobacter jejuni*	Retail chicken
CA21CH-0319-6-2	CA21CH-0319-6-2	SRR26215220	SAMN37598550	PRJNA1022324	*Campylobacter jejuni*	Retail chicken
CA21CH-0319-6-3	CA21CH-0319-6-3	SRR26215219	SAMN37598551	PRJNA1022324	*Campylobacter jejuni*	Retail chicken
CA21CH-0319-6-4	CA21CH-0319-6-4	SRR26215218	SAMN37598552	PRJNA1022324	*Campylobacter jejuni*	Retail chicken
CA21CH-0319-6-5	CA21CH-0319-6-5	SRR26215217	SAMN37598553	PRJNA1022324	*Campylobacter jejuni*	Retail chicken
CA21CH-0319-6-6	CA21CH-0319-6-6	SRR26215216	SAMN37598554	PRJNA1022324	*Campylobacter jejuni*	Retail chicken
CA21CH-0319-6-7	CA21CH-0319-6-7	SRR26215215	SAMN37598555	PRJNA1022324	*Campylobacter jejuni*	Retail chicken
CA21CH-0319-6-8	CA21CH-0319-6-8	SRR26215214	SAMN37598556	PRJNA1022324	*Campylobacter jejuni*	Retail chicken
CA21CH-0320-2-1	CA21CH-0320-2-1-R	SRR26215212	SAMN37598557	PRJNA1022324	*Campylobacter lari*	Retail chicken
CA21CH-0320-2-2	CA21CH-0320-2-2-R	SRR26215211	SAMN37598558	PRJNA1022324	*Campylobacter lari*	Retail chicken
CA21CH-0320-2-3	CA21CH-0320-2-3-R	SRR26215210	SAMN37598559	PRJNA1022324	*Campylobacter lari*	Retail chicken
CA21CH-0320-2-4	CA21CH-0320-2-4-R	SRR26215097	SAMN37598560	PRJNA1022324	*Campylobacter lari*	Retail chicken
CA21CH-0320-2-5	CA21CH-0320-2-5-R	SRR26215096	SAMN37598561	PRJNA1022324	*Campylobacter lari*	Retail chicken
CA21CH-0320-2-6	CA21CH-0320-2-6-R	SRR26215095	SAMN37598562	PRJNA1022324	*Campylobacter lari*	Retail chicken
CA21CH-0320-2-7	CA21CH-0320-2-7-R	SRR26215094	SAMN37598563	PRJNA1022324	*Campylobacter lari*	Retail chicken

CA21CH-0320-2-8	CA21CH-0320-2-8-R	SRR26215093	SAMN37598564	PRJNA1022324	*Campylobacter lari*	Retail chicken
CA21CH-0320-3-1	CA21CH-0320-3-1	SRR26215092	SAMN37598565	PRJNA1022324	*Campylobacter lari*	Retail chicken
CA21CH-0320-3-2	CA21CH-0320-3-2	SRR26215091	SAMN37598566	PRJNA1022324	*Campylobacter lari*	Retail chicken
CA21CH-0320-3-3	CA21CH-0320-3-3	SRR26215494	SAMN37598567	PRJNA1022324	*Campylobacter lari*	Retail chicken
CA21CH-0320-3-4	CA21CH-0320-3-4	SRR26215493	SAMN37598568	PRJNA1022324	*Campylobacter lari*	Retail chicken
CA21CH-0320-3-5	CA21CH-0320-3-5	SRR26215492	SAMN37598569	PRJNA1022324	*Campylobacter lari*	Retail chicken
CA21CH-0320-3-6	CA21CH-0320-3-6	SRR26215491	SAMN37598570	PRJNA1022324	*Campylobacter lari*	Retail chicken
CA21CH-0320-3-7	CA21CH-0320-3-7	SRR26215490	SAMN37598571	PRJNA1022324	*Campylobacter lari*	Retail chicken
CA21CH-0320-3-8	CA21CH-0320-3-8	SRR26215489	SAMN37598572	PRJNA1022324	*Campylobacter lari*	Retail chicken
CA21CH-0320-6-1	CA21CH-0320-6-1	SRR26215488	SAMN37598573	PRJNA1022324	*Campylobacter jejuni*	Retail chicken
CA21CH-0320-6-2	CA21CH-0320-6-2	SRR26215487	SAMN37598574	PRJNA1022324	*Campylobacter jejuni*	Retail chicken
CA21CH-0320-6-3	CA21CH-0320-6-3	SRR26215486	SAMN37598575	PRJNA1022324	*Campylobacter jejuni*	Retail chicken
CA21CH-0320-6-4	CA21CH-0320-6-4	SRR26215485	SAMN37598576	PRJNA1022324	*Campylobacter jejuni*	Retail chicken
CA21CH-0320-6-5	CA21CH-0320-6-5	SRR26215483	SAMN37598577	PRJNA1022324	*Campylobacter jejuni*	Retail chicken
CA21CH-0320-6-6	CA21CH-0320-6-6	SRR26215482	SAMN37598578	PRJNA1022324	*Campylobacter jejuni*	Retail chicken
CA21CH-0320-6-7	CA21CH-0320-6-7	SRR26215481	SAMN37598579	PRJNA1022324	*Campylobacter jejuni*	Retail chicken
CA21CH-0320-6-8	CA21CH-0320-6-8-R	SRR26215480	SAMN37598580	PRJNA1022324	*Campylobacter jejuni*	Retail chicken
CA21CH-0321-3-1	CA21CH-0321-3-1	SRR26215479	SAMN37598581	PRJNA1022324	*Campylobacter jejuni*	Retail chicken
CA21CH-0321-3-2	CA21CH-0321-3-2	SRR26215478	SAMN37598582	PRJNA1022324	*Campylobacter jejuni*	Retail chicken
CA21CH-0321-3-3	CA21CH-0321-3-3	SRR26215477	SAMN37598583	PRJNA1022324	*Campylobacter jejuni*	Retail chicken
CA21CH-0321-3-4	CA21CH-0321-3-4	SRR26215476	SAMN37598584	PRJNA1022324	*Campylobacter jejuni*	Retail chicken
CA21CH-0321-4-1	CA21CH-0321-4-1	SRR26215475	SAMN37598585	PRJNA1022324	*Campylobacter jejuni*	Retail chicken
CA21CH-0321-4-2	CA21CH-0321-4-2	SRR26215474	SAMN37598586	PRJNA1022324	*Campylobacter jejuni*	Retail chicken
CA21CH-0321-6-1	CA21CH-0321-6-1	SRR26215472	SAMN37598587	PRJNA1022324	*Campylobacter jejuni*	Retail chicken
CA21CH-0321-6-2	CA21CH-0321-6-2	SRR26215471	SAMN37598588	PRJNA1022324	*Campylobacter jejuni*	Retail chicken
CA21CH-0321-6-3	CA21CH-0321-6-3	SRR26215470	SAMN37598589	PRJNA1022324	*Campylobacter jejuni*	Retail chicken
CA21CH-0321-6-4	CA21CH-0321-6-4	SRR26215469	SAMN37598590	PRJNA1022324	*Campylobacter jejuni*	Retail chicken
CA21CH-0321-6-5	CA21CH-0321-6-5	SRR26215468	SAMN37598591	PRJNA1022324	*Campylobacter jejuni*	Retail chicken
CA21CH-0321-6-6	CA21CH-0321-6-6	SRR26215467	SAMN37598592	PRJNA1022324	*Campylobacter jejuni*	Retail chicken
CA21CH-0321-6-7	CA21CH-0321-6-7	SRR26215466	SAMN37598593	PRJNA1022324	*Campylobacter jejuni*	Retail chicken
CA21CH-0321-6-8	CA21CH-0321-6-8	SRR26215465	SAMN37598594	PRJNA1022324	*Campylobacter jejuni*	Retail chicken
CA21CH-0323-6-1	CA21CH-0323-6-1	SRR26215464	SAMN37598595	PRJNA1022324	*Campylobacter jejuni*	Retail chicken
CA21CH-0323-6-2	CA21CH-0323-6-2	SRR26215463	SAMN37598596	PRJNA1022324	*Campylobacter jejuni*	Retail chicken
CA21CH-0323-6-3	CA21CH-0323-6-3	SRR26215461	SAMN37598597	PRJNA1022324	*Campylobacter jejuni*	Retail chicken
CA21CH-0323-6-4	CA21CH-0323-6-4	SRR26215460	SAMN37598598	PRJNA1022324	*Campylobacter jejuni*	Retail chicken
CA21CH-0323-6-5	CA21CH-0323-6-5-R	SRR26215459	SAMN37598599	PRJNA1022324	*Campylobacter jejuni*	Retail chicken
CA21CH-0323-6-6	CA21CH-0323-6-6	SRR26215458	SAMN37598600	PRJNA1022324	*Campylobacter jejuni*	Retail chicken
CA21CH-0323-6-7	CA21CH-0323-6-7	SRR26215457	SAMN37598601	PRJNA1022324	*Campylobacter jejuni*	Retail chicken
CA21CH-0325-2-1	CA21CH-0325-2-1	SRR26215456	SAMN37598602	PRJNA1022324	*Campylobacter jejuni*	Retail chicken
CA21CH-0325-3-1	CA21CH-0325-3-1	SRR26215455	SAMN37598603	PRJNA1022324	*Campylobacter coli*	Retail chicken

CA21CH-0325-3-2	CA21CH-0325-3-2	SRR26215454	SAMN37598604	PRJNA1022324	*Campylobacter jejuni*	Retail chicken
CA21CH-0325-3-3	CA21CH-0325-3-3	SRR26215453	SAMN37598605	PRJNA1022324	*Campylobacter jejuni*	Retail chicken
CA21CH-0325-3-4	CA21CH-0325-3-4	SRR26215452	SAMN37598606	PRJNA1022324	*Campylobacter jejuni*	Retail chicken
CA21CH-0325-3-5	CA21CH-0325-3-5-R	SRR26215450	SAMN37598607	PRJNA1022324	*Campylobacter jejuni*	Retail chicken
CA21CH-0325-3-6	CA21CH-0325-3-6	SRR26215449	SAMN37598608	PRJNA1022324	*Campylobacter jejuni*	Retail chicken
CA21CH-0325-3-7	CA21CH-0325-3-7	SRR26215448	SAMN37598609	PRJNA1022324	*Campylobacter jejuni*	Retail chicken
CA21CH-0325-3-8	CA21CH-0325-3-8	SRR26215447	SAMN37598610	PRJNA1022324	*Campylobacter jejuni*	Retail chicken
CA21CH-0325-6-1	CA21CH-0325-6-1	SRR26215446	SAMN37598611	PRJNA1022324	*Campylobacter coli*	Retail chicken
CA21CH-0325-6-2	CA21CH-0325-6-2	SRR26215445	SAMN37598612	PRJNA1022324	*Campylobacter coli*	Retail chicken
CA21CH-0325-6-3	CA21CH-0325-6-3	SRR26215444	SAMN37598613	PRJNA1022324	*Campylobacter jejuni*	Retail chicken
CA21CH-0325-6-4	CA21CH-0325-6-4	SRR26215443	SAMN37598614	PRJNA1022324	*Campylobacter jejuni*	Retail chicken
CA21CH-0325-6-5	CA21CH-0325-6-5	SRR26215442	SAMN37598615	PRJNA1022324	*Campylobacter jejuni*	Retail chicken
CA21CH-0325-6-6	CA21CH-0325-6-6	SRR26215441	SAMN37598616	PRJNA1022324	*Campylobacter jejuni*	Retail chicken
CA21CH-0325-6-7	CA21CH-0325-6-7	SRR26215439	SAMN37598617	PRJNA1022324	*Campylobacter coli*	Retail chicken
CA21CH-0326-3-1	CA21CH-0326-3-1-R	SRR26215438	SAMN37598618	PRJNA1022324	*Campylobacter coli*	Retail chicken
CA21CH-0326-3-2	CA21CH-0326-3-2	SRR26215437	SAMN37598619	PRJNA1022324	*Campylobacter coli*	Retail chicken
CA21CH-0326-3-3	CA21CH-0326-3-3	SRR26215436	SAMN37598620	PRJNA1022324	*Campylobacter coli*	Retail chicken
CA21CH-0326-3-4	CA21CH-0326-3-4	SRR26215435	SAMN37598621	PRJNA1022324	*Campylobacter coli*	Retail chicken
CA21CH-0326-3-5	CA21CH-0326-3-5	SRR26215434	SAMN37598622	PRJNA1022324	*Campylobacter coli*	Retail chicken
CA21CH-0326-3-6	CA21CH-0326-3-6	SRR26215433	SAMN37598623	PRJNA1022324	*Campylobacter coli*	Retail chicken
CA21CH-0326-3-7	CA21CH-0326-3-7	SRR26215432	SAMN37598624	PRJNA1022324	*Campylobacter coli*	Retail chicken
CA21CH-0326-3-8	CA21CH-0326-3-8	SRR26215431	SAMN37598625	PRJNA1022324	*Campylobacter coli*	Retail chicken
CA21CH-0326-5-1	CA21CH-0326-5-1-R	SRR26215430	SAMN37598626	PRJNA1022324	*Campylobacter coli*	Retail chicken
CA21CH-0326-5-2	CA21CH-0326-5-2	SRR26215428	SAMN37598627	PRJNA1022324	*Campylobacter coli*	Retail chicken
CA21CH-0326-5-3	CA21CH-0326-5-3	SRR26215427	SAMN37598628	PRJNA1022324	*Campylobacter coli*	Retail chicken
CA21CH-0326-5-4	CA21CH-0326-5-4	SRR26215426	SAMN37598629	PRJNA1022324	*Campylobacter coli*	Retail chicken
CA21CH-0326-5-5	CA21CH-0326-5-5	SRR26215425	SAMN37598630	PRJNA1022324	*Campylobacter coli*	Retail chicken
CA21CH-0326-5-6	CA21CH-0326-5-6	SRR26215424	SAMN37598631	PRJNA1022324	*Campylobacter coli*	Retail chicken
CA21CH-0326-6-1	CA21CH-0326-6-1	SRR26215423	SAMN37598632	PRJNA1022324	*Campylobacter coli*	Retail chicken
CA21CH-0327-5-1	CA21CH-0327-5-1	SRR26215422	SAMN37598633	PRJNA1022324	*Campylobacter coli*	Retail chicken
CA21CH-0327-5-2	CA21CH-0327-5-2	SRR26215421	SAMN37598634	PRJNA1022324	*Campylobacter coli*	Retail chicken
CA21CH-0327-5-3	CA21CH-0327-5-3	SRR26215420	SAMN37598635	PRJNA1022324	*Campylobacter coli*	Retail chicken
CA21CH-0327-5-4	CA21CH-0327-5-4	SRR26215419	SAMN37598636	PRJNA1022324	*Campylobacter coli*	Retail chicken
CA21CH-0327-5-5	CA21CH-0327-5-5	SRR26215417	SAMN37598637	PRJNA1022324	*Campylobacter coli*	Retail chicken
CA21CH-0327-5-6	CA21CH-0327-5-6	SRR26215416	SAMN37598638	PRJNA1022324	*Campylobacter coli*	Retail chicken
CA21CH-0327-5-7	CA21CH-0327-5-7	SRR26215415	SAMN37598639	PRJNA1022324	*Campylobacter coli*	Retail chicken
CA21CH-0327-5-8	CA21CH-0327-5-8	SRR26215414	SAMN37598640	PRJNA1022324	*Campylobacter coli*	Retail chicken
CA21CH-0327-6-1	CA21CH-0327-6-1	SRR26215413	SAMN37598641	PRJNA1022324	*Campylobacter jejuni*	Retail chicken
CA21CH-0327-6-2	CA21CH-0327-6-2	SRR26215412	SAMN37598642	PRJNA1022324	*Campylobacter jejuni*	Retail chicken
CA21CH-0327-6-3	CA21CH-0327-6-3	SRR26215411	SAMN37598643	PRJNA1022324	*Campylobacter jejuni*	Retail chicken

CA21CH-0327-6-4	CA21CH-0327-6-4	SRR26215410	SAMN37598644	PRJNA1022324	*Campylobacter jejuni*	Retail chicken
CA21CH-0327-6-5	CA21CH-0327-6-5	SRR26215409	SAMN37598645	PRJNA1022324	*Campylobacter jejuni*	Retail chicken
CA21CH-0327-6-6	CA21CH-0327-6-6	SRR26215408	SAMN37598646	PRJNA1022324	*Campylobacter jejuni*	Retail chicken
CA21CH-0327-6-7	CA21CH-0327-6-7	SRR26215406	SAMN37598647	PRJNA1022324	*Campylobacter jejuni*	Retail chicken
CA21CH-0328-3-1	CA21CH-0328-3-1	SRR26215405	SAMN37598648	PRJNA1022324	*Campylobacter jejuni*	Retail chicken
CA21CH-0328-3-2	CA21CH-0328-3-2	SRR26215404	SAMN37598649	PRJNA1022324	*Campylobacter jejuni*	Retail chicken
CA21CH-0328-3-3	CA21CH-0328-3-3	SRR26215403	SAMN37598650	PRJNA1022324	*Campylobacter jejuni*	Retail chicken
CA21CH-0328-3-4	CA21CH-0328-3-4	SRR26215402	SAMN37598651	PRJNA1022324	*Campylobacter jejuni*	Retail chicken
CA21CH-0328-3-5	CA21CH-0328-3-5	SRR26215209	SAMN37598652	PRJNA1022324	*Campylobacter jejuni*	Retail chicken
CA21CH-0328-3-6	CA21CH-0328-3-6	SRR26215208	SAMN37598653	PRJNA1022324	*Campylobacter jejuni*	Retail chicken
CA21CH-0328-3-7	CA21CH-0328-3-7	SRR26215207	SAMN37598654	PRJNA1022324	*Campylobacter jejuni*	Retail chicken
CA21CH-0328-3-8	CA21CH-0328-3-8	SRR26215206	SAMN37598655	PRJNA1022324	*Campylobacter jejuni*	Retail chicken
CA21CH-0328-5-1	CA21CH-0328-5-1	SRR26215205	SAMN37598656	PRJNA1022324	*Campylobacter jejuni*	Retail chicken
CA21CH-0328-5-2	CA21CH-0328-5-2	SRR26215203	SAMN37598657	PRJNA1022324	*Campylobacter jejuni*	Retail chicken
CA21CH-0328-5-3	CA21CH-0328-5-3	SRR26215202	SAMN37598658	PRJNA1022324	*Campylobacter jejuni*	Retail chicken
CA21CH-0328-5-4	CA21CH-0328-5-4	SRR26215201	SAMN37598659	PRJNA1022324	*Campylobacter jejuni*	Retail chicken
CA21CH-0328-5-5	CA21CH-0328-5-5	SRR26215200	SAMN37598660	PRJNA1022324	*Campylobacter jejuni*	Retail chicken
CA21CH-0328-5-6	CA21CH-0328-5-6	SRR26215199	SAMN37598661	PRJNA1022324	*Campylobacter jejuni*	Retail chicken
CA21CH-0328-5-7	CA21CH-0328-5-7	SRR26215198	SAMN37598662	PRJNA1022324	*Campylobacter jejuni*	Retail chicken
CA21CH-0328-5-8	CA21CH-0328-5-8	SRR26215197	SAMN37598663	PRJNA1022324	*Campylobacter jejuni*	Retail chicken
CA21CH-0328-6-1	CA21CH-0328-6-1	SRR26215196	SAMN37598664	PRJNA1022324	*Campylobacter jejuni*	Retail chicken
CA21CH-0329-2-1	CA21CH-0329-2-1	SRR26215195	SAMN37598665	PRJNA1022324	*Campylobacter coli*	Retail chicken
CA21CH-0329-2-2	CA21CH-0329-2-2	SRR26215194	SAMN37598666	PRJNA1022324	*Campylobacter coli*	Retail chicken
CA21CH-0329-2-3	CA21CH-0329-2-3	SRR26215191	SAMN37598667	PRJNA1022324	*Campylobacter coli*	Retail chicken
CA21CH-0329-2-4	CA21CH-0329-2-4	SRR26215190	SAMN37598668	PRJNA1022324	*Campylobacter coli*	Retail chicken
CA21CH-0329-2-5	CA21CH-0329-2-5	SRR26215189	SAMN37598669	PRJNA1022324	*Campylobacter coli*	Retail chicken
CA21CH-0329-2-6	CA21CH-0329-2-6	SRR26215188	SAMN37598670	PRJNA1022324	*Campylobacter coli*	Retail chicken
CA21CH-0329-2-7	CA21CH-0329-2-7	SRR26215187	SAMN37598671	PRJNA1022324	*Campylobacter coli*	Retail chicken
CA21CH-0329-2-8	CA21CH-0329-2-8	SRR26215186	SAMN37598672	PRJNA1022324	*Campylobacter coli*	Retail chicken
CA21CH-0329-3-1	CA21CH-0329-3-1	SRR26215185	SAMN37598673	PRJNA1022324	*Campylobacter coli*	Retail chicken
CA21CH-0329-3-2	CA21CH-0329-3-2	SRR26215184	SAMN37598674	PRJNA1022324	*Campylobacter coli*	Retail chicken
CA21CH-0329-3-3	CA21CH-0329-3-3	SRR26215183	SAMN37598675	PRJNA1022324	*Campylobacter coli*	Retail chicken
CA21CH-0329-3-4	CA21CH-0329-3-4	SRR26215182	SAMN37598676	PRJNA1022324	*Campylobacter coli*	Retail chicken
CA21CH-0329-3-5	CA21CH-0329-3-5	SRR26215180	SAMN37598677	PRJNA1022324	*Campylobacter coli*	Retail chicken
CA21CH-0329-3-6	CA21CH-0329-3-6	SRR26215179	SAMN37598678	PRJNA1022324	*Campylobacter coli*	Retail chicken
CA21CH-0329-3-7	CA21CH-0329-3-7	SRR26215178	SAMN37598679	PRJNA1022324	*Campylobacter coli*	Retail chicken
CA21CH-0329-3-8	CA21CH-0329-3-8	SRR26215401	SAMN37598680	PRJNA1022324	*Campylobacter coli*	Retail chicken
CA21CH-0329-5-1	CA21CH-0329-5-1	SRR26215400	SAMN37598681	PRJNA1022324	*Campylobacter coli*	Retail chicken
CA21CH-0329-5-2	CA21CH-0329-5-2	SRR26215399	SAMN37598682	PRJNA1022324	*Campylobacter coli*	Retail chicken
CA21CH-0329-5-3	CA21CH-0329-5-3	SRR26215398	SAMN37598683	PRJNA1022324	*Campylobacter coli*	Retail chicken

CA21CH-0329-5-4	CA21CH-0329-5-4	SRR26215397	SAMN37598684	PRJNA1022324	*Campylobacter coli*	Retail chicken
CA21CH-0329-5-5	CA21CH-0329-5-5	SRR26215396	SAMN37598685	PRJNA1022324	*Campylobacter coli*	Retail chicken
CA21CH-0329-5-6	CA21CH-0329-5-6	SRR26215395	SAMN37598686	PRJNA1022324	*Campylobacter coli*	Retail chicken
CA21CH-0329-5-7	CA21CH-0329-5-7	SRR26215393	SAMN37598687	PRJNA1022324	*Campylobacter coli*	Retail chicken
CA21CH-0329-5-8	CA21CH-0329-5-8	SRR26215392	SAMN37598688	PRJNA1022324	*Campylobacter coli*	Retail chicken
CA21CH-0329-6-1	CA21CH-0329-6-1	SRR26215391	SAMN37598689	PRJNA1022324	*Campylobacter coli*	Retail chicken
CA21CH-0329-6-2	CA21CH-0329-6-2	SRR26215390	SAMN37598690	PRJNA1022324	*Campylobacter coli*	Retail chicken
CA21CH-0329-6-3	CA21CH-0329-6-3	SRR26215389	SAMN37598691	PRJNA1022324	*Campylobacter coli*	Retail chicken
CA21CH-0329-6-4	CA21CH-0329-6-4	SRR26215388	SAMN37598692	PRJNA1022324	*Campylobacter coli*	Retail chicken
CA21CH-0329-6-5	CA21CH-0329-6-5	SRR26215387	SAMN37598693	PRJNA1022324	*Campylobacter coli*	Retail chicken
CA21CH-0329-6-6	CA21CH-0329-6-6	SRR26215386	SAMN37598694	PRJNA1022324	*Campylobacter coli*	Retail chicken
CA21CH-0329-6-7	CA21CH-0329-6-7	SRR26215385	SAMN37598695	PRJNA1022324	*Campylobacter coli*	Retail chicken
CA21CH-0329-6-8	CA21CH-0329-6-8	SRR26215384	SAMN37598696	PRJNA1022324	*Campylobacter coli*	Retail chicken
CA21CH-0330-2-1	CA21CH-0330-2-1	SRR26215382	SAMN37598697	PRJNA1022324	*Campylobacter coli*	Retail chicken
CA21CH-0330-2-2	CA21CH-0330-2-2	SRR26215381	SAMN37598698	PRJNA1022324	*Campylobacter coli*	Retail chicken
CA21CH-0330-2-3	CA21CH-0330-2-3	SRR26215380	SAMN37598699	PRJNA1022324	*Campylobacter coli*	Retail chicken
CA21CH-0330-2-4	CA21CH-0330-2-4	SRR26215379	SAMN37598700	PRJNA1022324	*Campylobacter coli*	Retail chicken
CA21CH-0330-2-5	CA21CH-0330-2-5	SRR26215378	SAMN37598701	PRJNA1022324	*Campylobacter coli*	Retail chicken
CA21CH-0330-2-6	CA21CH-0330-2-6	SRR26215377	SAMN37598702	PRJNA1022324	*Campylobacter coli*	Retail chicken
CA21CH-0330-2-7	CA21CH-0330-2-7	SRR26215376	SAMN37598703	PRJNA1022324	*Campylobacter coli*	Retail chicken
CA21CH-0330-2-8	CA21CH-0330-2-8	SRR26215375	SAMN37598704	PRJNA1022324	*Campylobacter coli*	Retail chicken
CA21CH-0330-5-1	CA21CH-0330-5-1	SRR26215374	SAMN37598705	PRJNA1022324	*Campylobacter coli*	Retail chicken
CA21CH-0330-5-2	CA21CH-0330-5-2	SRR26215373	SAMN37598706	PRJNA1022324	*Campylobacter coli*	Retail chicken
CA21CH-0330-5-3	CA21CH-0330-5-3	SRR26215371	SAMN37598707	PRJNA1022324	*Campylobacter coli*	Retail chicken
CA21CH-0330-5-4	CA21CH-0330-5-4	SRR26215370	SAMN37598708	PRJNA1022324	*Campylobacter coli*	Retail chicken
CA21CH-0330-5-5	CA21CH-0330-5-5	SRR26215177	SAMN37598709	PRJNA1022324	*Campylobacter coli*	Retail chicken
CA21CH-0330-5-6	CA21CH-0330-5-6	SRR26215176	SAMN37598710	PRJNA1022324	*Campylobacter coli*	Retail chicken
CA21CH-0330-5-7	CA21CH-0330-5-7	SRR26215175	SAMN37598711	PRJNA1022324	*Campylobacter coli*	Retail chicken
CA21CH-0330-5-8	CA21CH-0330-5-8	SRR26215174	SAMN37598712	PRJNA1022324	*Campylobacter coli*	Retail chicken
CA21CH-0330-6-1	CA21CH-0330-6-1	SRR26215173	SAMN37598713	PRJNA1022324	*Campylobacter jejuni*	Retail chicken
CA21CH-0330-6-2	CA21CH-0330-6-2	SRR26215172	SAMN37598714	PRJNA1022324	*Campylobacter coli*	Retail chicken
CA21CH-0330-6-3	CA21CH-0330-6-3	SRR26215171	SAMN37598715	PRJNA1022324	*Campylobacter jejuni*	Retail chicken
CA21CH-0330-6-4	CA21CH-0330-6-4	SRR26215170	SAMN37598716	PRJNA1022324	*Campylobacter jejuni*	Retail chicken
CA21CH-0330-6-5	CA21CH-0330-6-5	SRR26215168	SAMN37598717	PRJNA1022324	*Campylobacter coli*	Retail chicken
CA21CH-0330-6-6	CA21CH-0330-6-6	SRR26215167	SAMN37598718	PRJNA1022324	*Campylobacter coli*	Retail chicken
CA21CH-0330-6-7	CA21CH-0330-6-7	SRR26215166	SAMN37598719	PRJNA1022324	*Campylobacter coli*	Retail chicken
CA21CH-0330-6-8	CA21CH-0330-6-8	SRR26215165	SAMN37598720	PRJNA1022324	*Campylobacter jejuni*	Retail chicken
CA21CH-0331-2-1	CA21CH-0331-2-1	SRR26215164	SAMN37598721	PRJNA1022324	*Campylobacter jejuni*	Retail chicken
CA21CH-0331-2-2	CA21CH-0331-2-2	SRR26215163	SAMN37598722	PRJNA1022324	*Campylobacter jejuni*	Retail chicken
CA21CH-0331-2-3	CA21CH-0331-2-3	SRR26215162	SAMN37598723	PRJNA1022324	*Campylobacter jejuni*	Retail chicken

CA21CH-0331-2-4	CA21CH-0331-2-4	SRR26215090	SAMN37598724	PRJNA1022324	*Campylobacter jejuni*	Retail chicken
CA21CH-0331-2-5	CA21CH-0331-2-5	SRR26215089	SAMN37598725	PRJNA1022324	*Campylobacter jejuni*	Retail chicken
CA21CH-0331-2-6	CA21CH-0331-2-6	SRR26215088	SAMN37598726	PRJNA1022324	*Campylobacter jejuni*	Retail chicken
CA21CH-0331-2-7	CA21CH-0331-2-7	SRR26215086	SAMN37598727	PRJNA1022324	*Campylobacter jejuni*	Retail chicken
CA21CH-0331-2-8	CA21CH-0331-2-8	SRR26215085	SAMN37598728	PRJNA1022324	*Campylobacter jejuni*	Retail chicken
CA21CH-0331-5-1	CA21CH-0331-5-1	SRR26215084	SAMN37598729	PRJNA1022324	*Campylobacter jejuni*	Retail chicken
CA21CH-0331-5-2	CA21CH-0331-5-2	SRR26215083	SAMN37598730	PRJNA1022324	*Campylobacter jejuni*	Retail chicken
CA21CH-0331-5-3	CA21CH-0331-5-3	SRR26215082	SAMN37598731	PRJNA1022324	*Campylobacter jejuni*	Retail chicken
CA21CH-0331-5-4	CA21CH-0331-5-4	SRR26215081	SAMN37598732	PRJNA1022324	*Campylobacter jejuni*	Retail chicken
CA21CH-0331-5-5	CA21CH-0331-5-5	SRR26215080	SAMN37598733	PRJNA1022324	*Campylobacter jejuni*	Retail chicken
CA21CH-0331-5-6	CA21CH-0331-5-6	SRR26215079	SAMN37598734	PRJNA1022324	*Campylobacter jejuni*	Retail chicken
CA21CH-0331-5-7	CA21CH-0331-5-7	SRR26215078	SAMN37598735	PRJNA1022324	*Campylobacter jejuni*	Retail chicken
CA21CH-0331-5-8	CA21CH-0331-5-8	SRR26215077	SAMN37598736	PRJNA1022324	*Campylobacter jejuni*	Retail chicken
CA21CH-0331-6-1	CA21CH-0331-6-1	SRR26215075	SAMN37598737	PRJNA1022324	*Campylobacter jejuni*	Retail chicken
CA21CH-0331-6-2	CA21CH-0331-6-2	SRR26215074	SAMN37598738	PRJNA1022324	*Campylobacter jejuni*	Retail chicken
CA21CH-0331-6-3	CA21CH-0331-6-3	SRR26215073	SAMN37598739	PRJNA1022324	*Campylobacter jejuni*	Retail chicken
CA21CH-0331-6-4	CA21CH-0331-6-4	SRR26215072	SAMN37598740	PRJNA1022324	*Campylobacter jejuni*	Retail chicken
CA21CH-0331-6-5	CA21CH-0331-6-5	SRR26215071	SAMN37598741	PRJNA1022324	*Campylobacter jejuni*	Retail chicken
CA21CH-0331-6-6	CA21CH-0331-6-6	SRR26215070	SAMN37598742	PRJNA1022324	*Campylobacter jejuni*	Retail chicken
CA21CH-0331-6-7	CA21CH-0331-6-7	SRR26215069	SAMN37598743	PRJNA1022324	*Campylobacter jejuni*	Retail chicken
CA21CH-0331-6-8	CA21CH-0331-6-8	SRR26215068	SAMN37598744	PRJNA1022324	*Campylobacter jejuni*	Retail chicken
CA21CH-0332-3-1	CA21CH-0332-3-1	SRR26215067	SAMN37598745	PRJNA1022324	*Campylobacter jejuni*	Retail chicken
CA21CH-0332-3-2	CA21CH-0332-3-2	SRR26215066	SAMN37598746	PRJNA1022324	*Campylobacter jejuni*	Retail chicken
CA21CH-0332-3-3	CA21CH-0332-3-3	SRR26215064	SAMN37598747	PRJNA1022324	*Campylobacter jejuni*	Retail chicken
CA21CH-0332-3-4	CA21CH-0332-3-4	SRR26215063	SAMN37598748	PRJNA1022324	*Campylobacter jejuni*	Retail chicken
CA21CH-0332-3-5	CA21CH-0332-3-5	SRR26215062	SAMN37598749	PRJNA1022324	*Campylobacter jejuni*	Retail chicken
CA21CH-0332-3-6	CA21CH-0332-3-6	SRR26215061	SAMN37598750	PRJNA1022324	*Campylobacter jejuni*	Retail chicken
CA21CH-0332-3-7	CA21CH-0332-3-7	SRR26215060	SAMN37598751	PRJNA1022324	*Campylobacter jejuni*	Retail chicken
CA21CH-0332-3-8	CA21CH-0332-3-8	SRR26215059	SAMN37598752	PRJNA1022324	*Campylobacter jejuni*	Retail chicken
CA21CH-0332-6-1	CA21CH-0332-6-1	SRR26215058	SAMN37598753	PRJNA1022324	*Campylobacter jejuni*	Retail chicken
CA21CH-0332-6-2	CA21CH-0332-6-2	SRR26215057	SAMN37598754	PRJNA1022324	*Campylobacter jejuni*	Retail chicken
CA21CH-0332-6-3	CA21CH-0332-6-3	SRR26215056	SAMN37598755	PRJNA1022324	*Campylobacter jejuni*	Retail chicken
CA21CH-0332-6-4	CA21CH-0332-6-4	SRR26215055	SAMN37598756	PRJNA1022324	*Campylobacter jejuni*	Retail chicken
CA21CH-0332-6-5	CA21CH-0332-6-5	SRR26215053	SAMN37598757	PRJNA1022324	*Campylobacter jejuni*	Retail chicken
CA21CH-0332-6-6	CA21CH-0332-6-6	SRR26215052	SAMN37598758	PRJNA1022324	*Campylobacter jejuni*	Retail chicken
CA21CH-0332-6-7	CA21CH-0332-6-7	SRR26215051	SAMN37598759	PRJNA1022324	*Campylobacter jejuni*	Retail chicken
CA21CH-0332-6-8	CA21CH-0332-6-8	SRR26215050	SAMN37598760	PRJNA1022324	*Campylobacter jejuni*	Retail chicken
CA21CH-0333-1-1	CA21CH-0333-1-1	SRR26215049	SAMN37598761	PRJNA1022324	*Campylobacter jejuni*	Retail chicken
CA21CH-0333-1-2	CA21CH-0333-1-2	SRR26215048	SAMN37598762	PRJNA1022324	*Campylobacter jejuni*	Retail chicken
CA21CH-0333-1-3	CA21CH-0333-1-3	SRR26215047	SAMN37598763	PRJNA1022324	*Campylobacter jejuni*	Retail chicken

CA21CH-0333-1-4	CA21CH-0333-1-4	SRR26215046	SAMN37598764	PRJNA1022324	*Campylobacter jejuni*	Retail chicken
CA21CH-0333-1-5	CA21CH-0333-1-5	SRR26215045	SAMN37598765	PRJNA1022324	*Campylobacter jejuni*	Retail chicken
CA21CH-0333-1-6	CA21CH-0333-1-6	SRR26215044	SAMN37598766	PRJNA1022324	*Campylobacter jejuni*	Retail chicken
CA21CH-0333-2-1	CA21CH-0333-2-1	SRR26215368	SAMN37598767	PRJNA1022324	*Campylobacter jejuni*	Retail chicken
CA21CH-0333-2-2	CA21CH-0333-2-2	SRR26215367	SAMN37598768	PRJNA1022324	*Campylobacter jejuni*	Retail chicken
CA21CH-0333-2-3	CA21CH-0333-2-3	SRR26215366	SAMN37598769	PRJNA1022324	*Campylobacter jejuni*	Retail chicken
CA21CH-0333-2-4	CA21CH-0333-2-4	SRR26215365	SAMN37598770	PRJNA1022324	*Campylobacter jejuni*	Retail chicken
CA21CH-0333-2-5	CA21CH-0333-2-5	SRR26215364	SAMN37598771	PRJNA1022324	*Campylobacter jejuni*	Retail chicken
CA21CH-0333-2-6	CA21CH-0333-2-6	SRR26215363	SAMN37598772	PRJNA1022324	*Campylobacter jejuni*	Retail chicken
CA21CH-0333-2-7	CA21CH-0333-2-7	SRR26215362	SAMN37598773	PRJNA1022324	*Campylobacter jejuni*	Retail chicken
CA21CH-0333-2-8	CA21CH-0333-2-8	SRR26215361	SAMN37598774	PRJNA1022324	*Campylobacter jejuni*	Retail chicken
CA21CH-0333-4-1	CA21CH-0333-4-1	SRR26215360	SAMN37598775	PRJNA1022324	*Campylobacter jejuni*	Retail chicken
CA21CH-0333-4-2	CA21CH-0333-4-2	SRR26215359	SAMN37598776	PRJNA1022324	*Campylobacter jejuni*	Retail chicken
CA21CH-0333-5-1	CA21CH-0333-5-1	SRR26215357	SAMN37598777	PRJNA1022324	*Campylobacter jejuni*	Retail chicken
CA21CH-0333-5-2	CA21CH-0333-5-2	SRR26215356	SAMN37598778	PRJNA1022324	*Campylobacter jejuni*	Retail chicken
CA21CH-0333-5-3	CA21CH-0333-5-3	SRR26215355	SAMN37598779	PRJNA1022324	*Campylobacter jejuni*	Retail chicken
CA21CH-0333-5-4	CA21CH-0333-5-4	SRR26215354	SAMN37598780	PRJNA1022324	*Campylobacter jejuni*	Retail chicken
CA21CH-0333-5-5	CA21CH-0333-5-5	SRR26215353	SAMN37598781	PRJNA1022324	*Campylobacter coli*	Retail chicken
CA21CH-0333-5-6	CA21CH-0333-5-6	SRR26215352	SAMN37598782	PRJNA1022324	*Campylobacter jejuni*	Retail chicken
CA21CH-0333-6-1	CA21CH-0333-6-1	SRR26215351	SAMN37598783	PRJNA1022324	*Campylobacter coli*	Retail chicken
CA21CH-0333-6-2	CA21CH-0333-6-2	SRR26215350	SAMN37598784	PRJNA1022324	*Campylobacter jejuni*	Retail chicken
CA21CH-0333-6-3	CA21CH-0333-6-3	SRR26215349	SAMN37598785	PRJNA1022324	*Campylobacter jejuni*	Retail chicken
CA21CH-0334-2-1	CA21CH-0334-2-1	SRR26215348	SAMN37598786	PRJNA1022324	*Campylobacter jejuni*	Retail chicken
CA21CH-0334-2-2	CA21CH-0334-2-2	SRR26215346	SAMN37598787	PRJNA1022324	*Campylobacter jejuni*	Retail chicken
CA21CH-0334-2-3	CA21CH-0334-2-3	SRR26215345	SAMN37598788	PRJNA1022324	*Campylobacter jejuni*	Retail chicken
CA21CH-0334-2-4	CA21CH-0334-2-4	SRR26215344	SAMN37598789	PRJNA1022324	*Campylobacter jejuni*	Retail chicken
CA21CH-0334-2-5	CA21CH-0334-2-5	SRR26215343	SAMN37598790	PRJNA1022324	*Campylobacter jejuni*	Retail chicken
CA21CH-0334-2-6	CA21CH-0334-2-6	SRR26215342	SAMN37598791	PRJNA1022324	*Campylobacter jejuni*	Retail chicken
CA21CH-0334-2-7	CA21CH-0334-2-7	SRR26215341	SAMN37598792	PRJNA1022324	*Campylobacter jejuni*	Retail chicken
CA21CH-0334-2-8	CA21CH-0334-2-8	SRR26215340	SAMN37598793	PRJNA1022324	*Campylobacter jejuni*	Retail chicken
CA21CH-0334-4-1	CA21CH-0334-4-1	SRR26215339	SAMN37598794	PRJNA1022324	*Campylobacter jejuni*	Retail chicken
CA21CH-0334-4-2	CA21CH-0334-4-2	SRR26215338	SAMN37598795	PRJNA1022324	*Campylobacter jejuni*	Retail chicken
CA21CH-0334-4-3	CA21CH-0334-4-3	SRR26214978	SAMN37598796	PRJNA1022324	*Campylobacter jejuni*	Retail chicken
CA21CH-0334-5-1	CA21CH-0334-5-1	SRR26214976	SAMN37598797	PRJNA1022324	*Campylobacter jejuni*	Retail chicken
CA21CH-0334-5-2	CA21CH-0334-5-2	SRR26214975	SAMN37598798	PRJNA1022324	*Campylobacter jejuni*	Retail chicken
CA21CH-0334-5-3	CA21CH-0334-5-3	SRR26214974	SAMN37598799	PRJNA1022324	*Campylobacter jejuni*	Retail chicken
CA21CH-0334-5-4	CA21CH-0334-5-4	SRR26214973	SAMN37598800	PRJNA1022324	*Campylobacter jejuni*	Retail chicken
CA21CH-0334-5-5	CA21CH-0334-5-5	SRR26214972	SAMN37598801	PRJNA1022324	*Campylobacter jejuni*	Retail chicken
CA21CH-0334-5-6	CA21CH-0334-5-6	SRR26214971	SAMN37598802	PRJNA1022324	*Campylobacter jejuni*	Retail chicken
CA21CH-0334-5-7	CA21CH-0334-5-7	SRR26214970	SAMN37598803	PRJNA1022324	*Campylobacter jejuni*	Retail chicken

CA21CH-0334-5-8	CA21CH-0334-5-8	SRR26214969	SAMN37598804	PRJNA1022324	*Campylobacter jejuni*	Retail chicken
CA21CH-0334-6-1	CA21CH-0334-6-1	SRR26214968	SAMN37598805	PRJNA1022324	*Campylobacter jejuni*	Retail chicken
CA21CH-0334-6-2	CA21CH-0334-6-2	SRR26214967	SAMN37598806	PRJNA1022324	*Campylobacter jejuni*	Retail chicken
CA21CH-0334-6-3	CA21CH-0334-6-3	SRR26214965	SAMN37598807	PRJNA1022324	*Campylobacter jejuni*	Retail chicken
CA21CH-0334-6-4	CA21CH-0334-6-4	SRR26214964	SAMN37598808	PRJNA1022324	*Campylobacter jejuni*	Retail chicken
CA21CH-0334-6-5	CA21CH-0334-6-5	SRR26214963	SAMN37598809	PRJNA1022324	*Campylobacter jejuni*	Retail chicken
CA21CH-0335-1-1	CA21CH-0335-1-1	SRR26214962	SAMN37598810	PRJNA1022324	*Campylobacter jejuni*	Retail chicken
CA21CH-0335-1-2	CA21CH-0335-1-2	SRR26214961	SAMN37598811	PRJNA1022324	*Campylobacter jejuni*	Retail chicken
CA21CH-0335-2-1	CA21CH-0335-2-1	SRR26214960	SAMN37598812	PRJNA1022324	*Campylobacter coli*	Retail chicken
CA21CH-0335-2-2	CA21CH-0335-2-2	SRR26214959	SAMN37598813	PRJNA1022324	*Campylobacter coli*	Retail chicken
CA21CH-0335-2-3	CA21CH-0335-2-3	SRR26214958	SAMN37598814	PRJNA1022324	*Campylobacter coli*	Retail chicken
CA21CH-0335-2-4	CA21CH-0335-2-4	SRR26214957	SAMN37598815	PRJNA1022324	*Campylobacter jejuni*	Retail chicken
CA21CH-0335-2-5	CA21CH-0335-2-5	SRR26214956	SAMN37598816	PRJNA1022324	*Campylobacter coli*	Retail chicken
CA21CH-0335-2-6	CA21CH-0335-2-6	SRR26214954	SAMN37598817	PRJNA1022324	*Campylobacter coli*	Retail chicken
CA21CH-0335-2-7	CA21CH-0335-2-7	SRR26214953	SAMN37598818	PRJNA1022324	*Campylobacter coli*	Retail chicken
CA21CH-0335-2-8	CA21CH-0335-2-8	SRR26214952	SAMN37598819	PRJNA1022324	*Campylobacter coli*	Retail chicken
CA21CH-0335-3-1	CA21CH-0335-3-1	SRR26214951	SAMN37598820	PRJNA1022324	*Campylobacter coli*	Retail chicken
CA21CH-0335-3-2	CA21CH-0335-3-2	SRR26214950	SAMN37598821	PRJNA1022324	*Campylobacter jejuni*	Retail chicken
CA21CH-0335-3-3	CA21CH-0335-3-3	SRR26214949	SAMN37598822	PRJNA1022324	*Campylobacter coli*	Retail chicken
CA21CH-0335-3-4	CA21CH-0335-3-4	SRR26214948	SAMN37598823	PRJNA1022324	*Campylobacter coli*	Retail chicken
CA21CH-0335-3-5	CA21CH-0335-3-5	SRR26214947	SAMN37598824	PRJNA1022324	*Campylobacter coli*	Retail chicken
CA21CH-0335-3-6	CA21CH-0335-3-6	SRR26215337	SAMN37598825	PRJNA1022324	*Campylobacter coli*	Retail chicken
CA21CH-0335-3-7	CA21CH-0335-3-7	SRR26215336	SAMN37598826	PRJNA1022324	*Campylobacter coli*	Retail chicken
CA21CH-0335-3-8	CA21CH-0335-3-8	SRR26215334	SAMN37598827	PRJNA1022324	*Campylobacter coli*	Retail chicken
CA21CH-0335-4-1	CA21CH-0335-4-1	SRR26215333	SAMN37598828	PRJNA1022324	*Campylobacter jejuni*	Retail chicken
CA21CH-0335-5-1	CA21CH-0335-5-1	SRR26215332	SAMN37598829	PRJNA1022324	*Campylobacter coli*	Retail chicken
CA21CH-0335-5-2	CA21CH-0335-5-2	SRR26215331	SAMN37598830	PRJNA1022324	*Campylobacter coli*	Retail chicken
CA21CH-0335-5-3	CA21CH-0335-5-3	SRR26215330	SAMN37598831	PRJNA1022324	*Campylobacter coli*	Retail chicken
CA21CH-0335-5-4	CA21CH-0335-5-4	SRR26215329	SAMN37598832	PRJNA1022324	*Campylobacter coli*	Retail chicken
CA21CH-0335-5-5	CA21CH-0335-5-5	SRR26215328	SAMN37598833	PRJNA1022324	*Campylobacter coli*	Retail chicken
CA21CH-0335-5-6	CA21CH-0335-5-6	SRR26215327	SAMN37598834	PRJNA1022324	*Campylobacter coli*	Retail chicken
CA21CH-0335-5-7	CA21CH-0335-5-7	SRR26215326	SAMN37598835	PRJNA1022324	*Campylobacter coli*	Retail chicken
CA21CH-0335-5-8	CA21CH-0335-5-8	SRR26215325	SAMN37598836	PRJNA1022324	*Campylobacter coli*	Retail chicken
CA21CH-0335-6-1	CA21CH-0335-6-1	SRR26215323	SAMN37598837	PRJNA1022324	*Campylobacter coli*	Retail chicken
CA21CH-0335-6-2	CA21CH-0335-6-2	SRR26215322	SAMN37598838	PRJNA1022324	*Campylobacter coli*	Retail chicken
CA21CH-0335-6-3	CA21CH-0335-6-3	SRR26215321	SAMN37598839	PRJNA1022324	*Campylobacter coli*	Retail chicken
CA21CH-0335-6-4	CA21CH-0335-6-4	SRR26215320	SAMN37598840	PRJNA1022324	*Campylobacter coli*	Retail chicken
CA21CH-0335-6-5	CA21CH-0335-6-5	SRR26215319	SAMN37598841	PRJNA1022324	*Campylobacter coli*	Retail chicken
CA21CH-0335-6-6	CA21CH-0335-6-6	SRR26215318	SAMN37598842	PRJNA1022324	*Campylobacter coli*	Retail chicken
CA21CH-0335-6-7	CA21CH-0335-6-7	SRR26215317	SAMN37598843	PRJNA1022324	*Campylobacter coli*	Retail chicken

CA21CH-0335-6-8	CA21CH-0335-6-8	SRR26215316	SAMN37598844	PRJNA1022324	*Campylobacter jejuni*	Retail chicken
CA21CH-0336-2-1	CA21CH-0336-2-1	SRR26215315	SAMN37598845	PRJNA1022324	*Campylobacter jejuni*	Retail chicken
CA21CH-0336-2-2	CA21CH-0336-2-2	SRR26215314	SAMN37598846	PRJNA1022324	*Campylobacter jejuni*	Retail chicken
CA21CH-0336-2-3	CA21CH-0336-2-3	SRR26215312	SAMN37598847	PRJNA1022324	*Campylobacter jejuni*	Retail chicken
CA21CH-0336-2-4	CA21CH-0336-2-4	SRR26215311	SAMN37598848	PRJNA1022324	*Campylobacter jejuni*	Retail chicken
CA21CH-0336-2-5	CA21CH-0336-2-5	SRR26215310	SAMN37598849	PRJNA1022324	*Campylobacter jejuni*	Retail chicken
CA21CH-0336-2-6	CA21CH-0336-2-6	SRR26215309	SAMN37598850	PRJNA1022324	*Campylobacter jejuni*	Retail chicken
CA21CH-0336-2-7	CA21CH-0336-2-7	SRR26215308	SAMN37598851	PRJNA1022324	*Campylobacter jejuni*	Retail chicken
CA21CH-0336-2-8	CA21CH-0336-2-8	SRR26215307	SAMN37598852	PRJNA1022324	*Campylobacter jejuni*	Retail chicken
CA21CH-0336-3-1	CA21CH-0336-3-1	SRR26215306	SAMN37598853	PRJNA1022324	*Campylobacter jejuni*	Retail chicken
CA21CH-0336-3-2	CA21CH-0336-3-2	SRR26214946	SAMN37598854	PRJNA1022324	*Campylobacter jejuni*	Retail chicken
CA21CH-0336-3-3	CA21CH-0336-3-3	SRR26214945	SAMN37598855	PRJNA1022324	*Campylobacter jejuni*	Retail chicken
CA21CH-0336-3-4	CA21CH-0336-3-4	SRR26214944	SAMN37598856	PRJNA1022324	*Campylobacter jejuni*	Retail chicken
CA21CH-0336-3-5	CA21CH-0336-3-5	SRR26214942	SAMN37598857	PRJNA1022324	*Campylobacter jejuni*	Retail chicken
CA21CH-0336-3-6	CA21CH-0336-3-6	SRR26214941	SAMN37598858	PRJNA1022324	*Campylobacter jejuni*	Retail chicken
CA21CH-0336-3-7	CA21CH-0336-3-7	SRR26214940	SAMN37598859	PRJNA1022324	*Campylobacter jejuni*	Retail chicken
CA21CH-0336-3-8	CA21CH-0336-3-8	SRR26214939	SAMN37598860	PRJNA1022324	*Campylobacter jejuni*	Retail chicken
CA21CH-0336-5-1	CA21CH-0336-5-1	SRR26214938	SAMN37598861	PRJNA1022324	*Campylobacter jejuni*	Retail chicken
CA21CH-0336-5-2	CA21CH-0336-5-2	SRR26214937	SAMN37598862	PRJNA1022324	*Campylobacter jejuni*	Retail chicken
CA21CH-0336-5-3	CA21CH-0336-5-3	SRR26214936	SAMN37598863	PRJNA1022324	*Campylobacter jejuni*	Retail chicken
CA21CH-0336-5-4	CA21CH-0336-5-4	SRR26214935	SAMN37598864	PRJNA1022324	*Campylobacter jejuni*	Retail chicken
CA21CH-0336-5-5	CA21CH-0336-5-5	SRR26214934	SAMN37598865	PRJNA1022324	*Campylobacter jejuni*	Retail chicken
CA21CH-0336-5-6	CA21CH-0336-5-6	SRR26214933	SAMN37598866	PRJNA1022324	*Campylobacter jejuni*	Retail chicken
CA21CH-0336-5-7	CA21CH-0336-5-7	SRR26214930	SAMN37598867	PRJNA1022324	*Campylobacter jejuni*	Retail chicken
CA21CH-0336-6-1	CA21CH-0336-6-1	SRR26214929	SAMN37598868	PRJNA1022324	*Campylobacter jejuni*	Retail chicken
CA21CH-0336-6-2	CA21CH-0336-6-2	SRR26214928	SAMN37598869	PRJNA1022324	*Campylobacter jejuni*	Retail chicken
CA21CH-0336-6-3	CA21CH-0336-6-3	SRR26214927	SAMN37598870	PRJNA1022324	*Campylobacter jejuni*	Retail chicken
CA21CH-0336-6-4	CA21CH-0336-6-4	SRR26214926	SAMN37598871	PRJNA1022324	*Campylobacter jejuni*	Retail chicken
CA21CH-0336-6-5	CA21CH-0336-6-5	SRR26214925	SAMN37598872	PRJNA1022324	*Campylobacter jejuni*	Retail chicken
CA21CH-0336-6-6	CA21CH-0336-6-6	SRR26214924	SAMN37598873	PRJNA1022324	*Campylobacter jejuni*	Retail chicken
CA21CH-0336-6-7	CA21CH-0336-6-7	SRR26214923	SAMN37598874	PRJNA1022324	*Campylobacter jejuni*	Retail chicken
CA21CH-0336-6-8	CA21CH-0336-6-8	SRR26214922	SAMN37598875	PRJNA1022324	*Campylobacter jejuni*	Retail chicken
CA21CH-0337-2-1	CA21CH-0337-2-1	SRR26214921	SAMN37598876	PRJNA1022324	*Campylobacter jejuni*	Retail chicken
CA21CH-0337-2-2	CA21CH-0337-2-2	SRR26214919	SAMN37598877	PRJNA1022324	*Campylobacter jejuni*	Retail chicken
CA21CH-0337-2-3	CA21CH-0337-2-3	SRR26214918	SAMN37598878	PRJNA1022324	*Campylobacter jejuni*	Retail chicken
CA21CH-0337-2-4	CA21CH-0337-2-4	SRR26214917	SAMN37598879	PRJNA1022324	*Campylobacter jejuni*	Retail chicken
CA21CH-0337-2-5	CA21CH-0337-2-5	SRR26214916	SAMN37598880	PRJNA1022324	*Campylobacter jejuni*	Retail chicken
CA21CH-0337-2-6	CA21CH-0337-2-6	SRR26214915	SAMN37598881	PRJNA1022324	*Campylobacter jejuni*	Retail chicken
CA21CH-0337-2-7	CA21CH-0337-2-7	SRR26214818	SAMN37598882	PRJNA1022324	*Campylobacter jejuni*	Retail chicken
CA21CH-0337-2-8	CA21CH-0337-2-8	SRR26214817	SAMN37598883	PRJNA1022324	*Campylobacter jejuni*	Retail chicken

CA21CH-0337-4-1	CA21CH-0337-4-1	SRR26214816	SAMN37598884	PRJNA1022324	*Campylobacter jejuni*	Retail chicken
CA21CH-0337-5-1	CA21CH-0337-5-1	SRR26214815	SAMN37598885	PRJNA1022324	*Campylobacter jejuni*	Retail chicken
CA21CH-0337-5-2	CA21CH-0337-5-2	SRR26214814	SAMN37598886	PRJNA1022324	*Campylobacter jejuni*	Retail chicken
CA21CH-0337-5-3	CA21CH-0337-5-3	SRR26214812	SAMN37598887	PRJNA1022324	*Campylobacter jejuni*	Retail chicken
CA21CH-0337-5-4	CA21CH-0337-5-4	SRR26214811	SAMN37598888	PRJNA1022324	*Campylobacter jejuni*	Retail chicken
CA21CH-0337-5-5	CA21CH-0337-5-5	SRR26214810	SAMN37598889	PRJNA1022324	*Campylobacter jejuni*	Retail chicken
CA21CH-0337-5-6	CA21CH-0337-5-6	SRR26214809	SAMN37598890	PRJNA1022324	*Campylobacter jejuni*	Retail chicken
CA21CH-0337-5-7	CA21CH-0337-5-7	SRR26214808	SAMN37598891	PRJNA1022324	*Campylobacter jejuni*	Retail chicken
CA21CH-0337-5-8	CA21CH-0337-5-8	SRR26214807	SAMN37598892	PRJNA1022324	*Campylobacter jejuni*	Retail chicken
CA21CH-0337-6-1	CA21CH-0337-6-1	SRR26214806	SAMN37598893	PRJNA1022324	*Campylobacter jejuni*	Retail chicken
CA21CH-0337-6-2	CA21CH-0337-6-2	SRR26214805	SAMN37598894	PRJNA1022324	*Campylobacter jejuni*	Retail chicken
CA21CH-0337-6-3	CA21CH-0337-6-3	SRR26214804	SAMN37598895	PRJNA1022324	*Campylobacter jejuni*	Retail chicken
CA21CH-0337-6-4	CA21CH-0337-6-4	SRR26214803	SAMN37598896	PRJNA1022324	*Campylobacter jejuni*	Retail chicken
CA21CH-0338-2-1	CA21CH-0338-2-1	SRR26214801	SAMN37598897	PRJNA1022324	*Campylobacter jejuni*	Retail chicken
CA21CH-0338-2-2	CA21CH-0338-2-2	SRR26214800	SAMN37598898	PRJNA1022324	*Campylobacter jejuni*	Retail chicken
CA21CH-0338-2-3	CA21CH-0338-2-3	SRR26214799	SAMN37598899	PRJNA1022324	*Campylobacter jejuni*	Retail chicken
CA21CH-0338-2-4	CA21CH-0338-2-4	SRR26214798	SAMN37598900	PRJNA1022324	*Campylobacter coli*	Retail chicken
CA21CH-0338-2-5	CA21CH-0338-2-5	SRR26214797	SAMN37598901	PRJNA1022324	*Campylobacter jejuni*	Retail chicken
CA21CH-0338-2-6	CA21CH-0338-2-6	SRR26214796	SAMN37598902	PRJNA1022324	*Campylobacter jejuni*	Retail chicken
CA21CH-0338-2-7	CA21CH-0338-2-7	SRR26214795	SAMN37598903	PRJNA1022324	*Campylobacter jejuni*	Retail chicken
CA21CH-0338-2-8	CA21CH-0338-2-8	SRR26214794	SAMN37598904	PRJNA1022324	*Campylobacter jejuni*	Retail chicken
CA21CH-0338-3-1	CA21CH-0338-3-1	SRR26214793	SAMN37598905	PRJNA1022324	*Campylobacter coli*	Retail chicken
CA21CH-0338-3-2	CA21CH-0338-3-2	SRR26214792	SAMN37598906	PRJNA1022324	*Campylobacter coli*	Retail chicken
CA21CH-0338-3-3	CA21CH-0338-3-3	SRR26214790	SAMN37598907	PRJNA1022324	*Campylobacter coli*	Retail chicken
CA21CH-0338-3-4	CA21CH-0338-3-4	SRR26214789	SAMN37598908	PRJNA1022324	*Campylobacter jejuni*	Retail chicken
CA21CH-0338-3-5	CA21CH-0338-3-5	SRR26214788	SAMN37598909	PRJNA1022324	*Campylobacter coli*	Retail chicken
CA21CH-0338-3-6	CA21CH-0338-3-6	SRR26214787	SAMN37598910	PRJNA1022324	*Campylobacter jejuni*	Retail chicken
CA21CH-0338-3-7	CA21CH-0338-3-7	SRR26214914	SAMN37598911	PRJNA1022324	*Campylobacter coli*	Retail chicken
CA21CH-0338-3-8	CA21CH-0338-3-8	SRR26214913	SAMN37598912	PRJNA1022324	*Campylobacter coli*	Retail chicken
CA21CH-0338-5-1	CA21CH-0338-5-1	SRR26214912	SAMN37598913	PRJNA1022324	*Campylobacter coli*	Retail chicken
CA21CH-0338-5-2	CA21CH-0338-5-2	SRR26214911	SAMN37598914	PRJNA1022324	*Campylobacter coli*	Retail chicken
CA21CH-0338-5-3	CA21CH-0338-5-3	SRR26214910	SAMN37598915	PRJNA1022324	*Campylobacter coli*	Retail chicken
CA21CH-0338-5-4	CA21CH-0338-5-4	SRR26214909	SAMN37598916	PRJNA1022324	*Campylobacter coli*	Retail chicken
CA21CH-0338-5-5	CA21CH-0338-5-5	SRR26214907	SAMN37598917	PRJNA1022324	*Campylobacter coli*	Retail chicken
CA21CH-0338-5-6	CA21CH-0338-5-6	SRR26214906	SAMN37598918	PRJNA1022324	*Campylobacter coli*	Retail chicken
CA21CH-0338-5-7	CA21CH-0338-5-7	SRR26214905	SAMN37598919	PRJNA1022324	*Campylobacter coli*	Retail chicken
CA21CH-0338-5-8	CA21CH-0338-5-8	SRR26214904	SAMN37598920	PRJNA1022324	*Campylobacter coli*	Retail chicken
CA21CH-0338-6-1	CA21CH-0338-6-1	SRR26214903	SAMN37598921	PRJNA1022324	*Campylobacter coli*	Retail chicken
CA21CH-0338-6-2	CA21CH-0338-6-2	SRR26214902	SAMN37598922	PRJNA1022324	*Campylobacter coli*	Retail chicken
CA21CH-0338-6-3	CA21CH-0338-6-3	SRR26214901	SAMN37598923	PRJNA1022324	*Campylobacter coli*	Retail chicken

CA21CH-0338-6-4	CA21CH-0338-6-4	SRR26214900	SAMN37598924	PRJNA1022324	*Campylobacter coli*	Retail chicken
CA21CH-0338-6-5	CA21CH-0338-6-5	SRR26214899	SAMN37598925	PRJNA1022324	*Campylobacter coli*	Retail chicken
CA21CH-0338-6-6	CA21CH-0338-6-6	SRR26214898	SAMN37598926	PRJNA1022324	*Campylobacter coli*	Retail chicken
CA21CH-0338-6-7	CA21CH-0338-6-7	SRR26214896	SAMN37598927	PRJNA1022324	*Campylobacter coli*	Retail chicken
CA21CH-0338-6-8	CA21CH-0338-6-8	SRR26214895	SAMN37598928	PRJNA1022324	*Campylobacter coli*	Retail chicken
CA21CH-0339-2-1	CA21CH-0339-2-1	SRR26214894	SAMN37598929	PRJNA1022324	*Campylobacter jejuni*	Retail chicken
CA21CH-0339-2-2	CA21CH-0339-2-2	SRR26214893	SAMN37598930	PRJNA1022324	*Campylobacter jejuni*	Retail chicken
CA21CH-0339-2-3	CA21CH-0339-2-3	SRR26214892	SAMN37598931	PRJNA1022324	*Campylobacter jejuni*	Retail chicken
CA21CH-0339-2-4	CA21CH-0339-2-4	SRR26214891	SAMN37598932	PRJNA1022324	*Campylobacter jejuni*	Retail chicken
CA21CH-0339-2-5	CA21CH-0339-2-5	SRR26214890	SAMN37598933	PRJNA1022324	*Campylobacter jejuni*	Retail chicken
CA21CH-0339-2-6	CA21CH-0339-2-6	SRR26214889	SAMN37598934	PRJNA1022324	*Campylobacter jejuni*	Retail chicken
CA21CH-0339-2-7	CA21CH-0339-2-7	SRR26214888	SAMN37598935	PRJNA1022324	*Campylobacter jejuni*	Retail chicken
CA21CH-0339-2-8	CA21CH-0339-2-8	SRR26214887	SAMN37598936	PRJNA1022324	*Campylobacter jejuni*	Retail chicken
CA21CH-0339-3-1	CA21CH-0339-3-1	SRR26214885	SAMN37598937	PRJNA1022324	*Campylobacter jejuni*	Retail chicken
CA21CH-0339-3-2	CA21CH-0339-3-2	SRR26214884	SAMN37598938	PRJNA1022324	*Campylobacter jejuni*	Retail chicken
CA21CH-0339-3-3	CA21CH-0339-3-3	SRR26214883	SAMN37598939	PRJNA1022324	*Campylobacter jejuni*	Retail chicken
CA21CH-0339-3-4	CA21CH-0339-3-4	SRR26214786	SAMN37598940	PRJNA1022324	*Campylobacter jejuni*	Retail chicken
CA21CH-0339-3-5	CA21CH-0339-3-5	SRR26214785	SAMN37598941	PRJNA1022324	*Campylobacter jejuni*	Retail chicken
CA21CH-0339-3-6	CA21CH-0339-3-6	SRR26214784	SAMN37598942	PRJNA1022324	*Campylobacter jejuni*	Retail chicken
CA21CH-0339-3-7	CA21CH-0339-3-7	SRR26214783	SAMN37598943	PRJNA1022324	*Campylobacter jejuni*	Retail chicken
CA21CH-0339-3-8	CA21CH-0339-3-8	SRR26214782	SAMN37598944	PRJNA1022324	*Campylobacter jejuni*	Retail chicken
CA21CH-0339-5-1	CA21CH-0339-5-1	SRR26214781	SAMN37598945	PRJNA1022324	*Campylobacter jejuni*	Retail chicken
CA21CH-0339-5-2	CA21CH-0339-5-2	SRR26214780	SAMN37598946	PRJNA1022324	*Campylobacter jejuni*	Retail chicken
CA21CH-0339-5-3	CA21CH-0339-5-3	SRR26214778	SAMN37598947	PRJNA1022324	*Campylobacter jejuni*	Retail chicken
CA21CH-0339-5-4	CA21CH-0339-5-4	SRR26214777	SAMN37598948	PRJNA1022324	*Campylobacter jejuni*	Retail chicken
CA21CH-0339-5-5	CA21CH-0339-5-5	SRR26214776	SAMN37598949	PRJNA1022324	*Campylobacter jejuni*	Retail chicken
CA21CH-0339-5-6	CA21CH-0339-5-6	SRR26214775	SAMN37598950	PRJNA1022324	*Campylobacter jejuni*	Retail chicken
CA21CH-0339-5-7	CA21CH-0339-5-7	SRR26214774	SAMN37598951	PRJNA1022324	*Campylobacter jejuni*	Retail chicken
CA21CH-0339-5-8	CA21CH-0339-5-8	SRR26214773	SAMN37598952	PRJNA1022324	*Campylobacter jejuni*	Retail chicken
CA21CH-0339-6-1	CA21CH-0339-6-1	SRR26214772	SAMN37598953	PRJNA1022324	*Campylobacter jejuni*	Retail chicken
CA21CH-0339-6-2	CA21CH-0339-6-2	SRR26214771	SAMN37598954	PRJNA1022324	*Campylobacter jejuni*	Retail chicken
CA21CH-0339-6-3	CA21CH-0339-6-3	SRR26214770	SAMN37598955	PRJNA1022324	*Campylobacter coli*	Retail chicken
CA21CH-0339-6-4	CA21CH-0339-6-4	SRR26214769	SAMN37598956	PRJNA1022324	*Campylobacter coli*	Retail chicken
CA21CH-0339-6-5	CA21CH-0339-6-5	SRR26214767	SAMN37598957	PRJNA1022324	*Campylobacter coli*	Retail chicken
CA21CH-0339-6-6	CA21CH-0339-6-6	SRR26214766	SAMN37598958	PRJNA1022324	*Campylobacter coli*	Retail chicken
CA21CH-0339-6-7	CA21CH-0339-6-7	SRR26214765	SAMN37598959	PRJNA1022324	*Campylobacter jejuni*	Retail chicken
CA21CH-0340-1-1	CA21CH-0340-1-1	SRR26214764	SAMN37598960	PRJNA1022324	*Campylobacter jejuni*	Retail chicken
CA21CH-0340-2-1	CA21CH-0340-2-1	SRR26214763	SAMN37598961	PRJNA1022324	*Campylobacter jejuni*	Retail chicken
CA21CH-0340-2-2	CA21CH-0340-2-2	SRR26214762	SAMN37598962	PRJNA1022324	*Campylobacter jejuni*	Retail chicken
CA21CH-0340-2-3	CA21CH-0340-2-3	SRR26214761	SAMN37598963	PRJNA1022324	*Campylobacter jejuni*	Retail chicken

CA21CH-0340-2-4	CA21CH-0340-2-4	SRR26214760	SAMN37598964	PRJNA1022324	*Campylobacter jejuni*	Retail chicken
CA21CH-0340-2-5	CA21CH-0340-2-5	SRR26214759	SAMN37598965	PRJNA1022324	*Campylobacter jejuni*	Retail chicken
CA21CH-0340-2-6	CA21CH-0340-2-6	SRR26214758	SAMN37598966	PRJNA1022324	*Campylobacter jejuni*	Retail chicken
CA21CH-0340-2-7	CA21CH-0340-2-7	SRR26214755	SAMN37598967	PRJNA1022324	*Campylobacter jejuni*	Retail chicken
CA21CH-0340-2-8	CA21CH-0340-2-8	SRR26214882	SAMN37598968	PRJNA1022324	*Campylobacter jejuni*	Retail chicken
CA21CH-0340-3-1	CA21CH-0340-3-1	SRR26214881	SAMN37598969	PRJNA1022324	*Campylobacter jejuni*	Retail chicken
CA21CH-0340-3-2	CA21CH-0340-3-2	SRR26214880	SAMN37598970	PRJNA1022324	*Campylobacter jejuni*	Retail chicken
CA21CH-0340-3-3	CA21CH-0340-3-3	SRR26214879	SAMN37598971	PRJNA1022324	*Campylobacter jejuni*	Retail chicken
CA21CH-0340-3-4	CA21CH-0340-3-4	SRR26214878	SAMN37598972	PRJNA1022324	*Campylobacter jejuni*	Retail chicken
CA21CH-0340-5-1	CA21CH-0340-5-1	SRR26214877	SAMN37598973	PRJNA1022324	*Campylobacter jejuni*	Retail chicken
CA21CH-0340-5-2	CA21CH-0340-5-2	SRR26214876	SAMN37598974	PRJNA1022324	*Campylobacter jejuni*	Retail chicken
CA21CH-0340-5-3	CA21CH-0340-5-3	SRR26214875	SAMN37598975	PRJNA1022324	*Campylobacter jejuni*	Retail chicken
CA21CH-0340-5-4	CA21CH-0340-5-4	SRR26214874	SAMN37598976	PRJNA1022324	*Campylobacter jejuni*	Retail chicken
CA21CH-0340-5-5	CA21CH-0340-5-5	SRR26214872	SAMN37598977	PRJNA1022324	*Campylobacter coli*	Retail chicken
CA21CH-0340-5-6	CA21CH-0340-5-6	SRR26214871	SAMN37598978	PRJNA1022324	*Campylobacter coli*	Retail chicken
CA21CH-0340-5-7	CA21CH-0340-5-7	SRR26214870	SAMN37598979	PRJNA1022324	*Campylobacter coli*	Retail chicken
CA21CH-0340-5-8	CA21CH-0340-5-8	SRR26214869	SAMN37598980	PRJNA1022324	*Campylobacter jejuni*	Retail chicken
CA21CH-0340-6-1	CA21CH-0340-6-1	SRR26214868	SAMN37598981	PRJNA1022324	*Campylobacter coli*	Retail chicken
CA21CH-0340-6-2	CA21CH-0340-6-2	SRR26214867	SAMN37598982	PRJNA1022324	*Campylobacter coli*	Retail chicken
CA21CH-0340-6-3	CA21CH-0340-6-3	SRR26214866	SAMN37598983	PRJNA1022324	*Campylobacter jejuni*	Retail chicken
CA21CH-0340-6-4	CA21CH-0340-6-4	SRR26214865	SAMN37598984	PRJNA1022324	*Campylobacter coli*	Retail chicken
CA21CH-0340-6-5	CA21CH-0340-6-5	SRR26214864	SAMN37598985	PRJNA1022324	*Campylobacter coli*	Retail chicken
CA21CH-0340-6-6	CA21CH-0340-6-6	SRR26214863	SAMN37598986	PRJNA1022324	*Campylobacter coli*	Retail chicken
CA21CH-0340-6-7	CA21CH-0340-6-7	SRR26214861	SAMN37598987	PRJNA1022324	*Campylobacter coli*	Retail chicken
CA21CH-0341-2-1	CA21CH-0341-2-1	SRR26214860	SAMN37598988	PRJNA1022324	*Campylobacter coli*	Retail chicken
CA21CH-0341-2-2	CA21CH-0341-2-2	SRR26214859	SAMN37598989	PRJNA1022324	*Campylobacter coli*	Retail chicken
CA21CH-0341-2-3	CA21CH-0341-2-3	SRR26214858	SAMN37598990	PRJNA1022324	*Campylobacter coli*	Retail chicken
CA21CH-0341-2-4	CA21CH-0341-2-4	SRR26214857	SAMN37598991	PRJNA1022324	*Campylobacter coli*	Retail chicken
CA21CH-0341-2-5	CA21CH-0341-2-5	SRR26214856	SAMN37598992	PRJNA1022324	*Campylobacter jejuni*	Retail chicken
CA21CH-0341-2-6	CA21CH-0341-2-6	SRR26214855	SAMN37598993	PRJNA1022324	*Campylobacter jejuni*	Retail chicken
CA21CH-0341-2-7	CA21CH-0341-2-7	SRR26214854	SAMN37598994	PRJNA1022324	*Campylobacter coli*	Retail chicken
CA21CH-0341-2-8	CA21CH-0341-2-8	SRR26214853	SAMN37598995	PRJNA1022324	*Campylobacter jejuni*	Retail chicken
CA21CH-0341-3-1	CA21CH-0341-3-1	SRR26214852	SAMN37598996	PRJNA1022324	*Campylobacter jejuni*	Retail chicken
CA21CH-0341-3-2	CA21CH-0341-3-2	SRR26215161	SAMN37598997	PRJNA1022324	*Campylobacter jejuni*	Retail chicken
CA21CH-0341-3-3	CA21CH-0341-3-3	SRR26215160	SAMN37598998	PRJNA1022324	*Campylobacter jejuni*	Retail chicken
CA21CH-0341-3-4	CA21CH-0341-3-4	SRR26215159	SAMN37598999	PRJNA1022324	*Campylobacter jejuni*	Retail chicken
CA21CH-0341-3-5	CA21CH-0341-3-5	SRR26215158	SAMN37599000	PRJNA1022324	*Campylobacter jejuni*	Retail chicken
CA21CH-0341-3-6	CA21CH-0341-3-6	SRR26215157	SAMN37599001	PRJNA1022324	*Campylobacter jejuni*	Retail chicken
CA21CH-0341-3-7	CA21CH-0341-3-7	SRR26215156	SAMN37599002	PRJNA1022324	*Campylobacter jejuni*	Retail chicken
CA21CH-0341-3-8	CA21CH-0341-3-8	SRR26215155	SAMN37599003	PRJNA1022324	*Campylobacter jejuni*	Retail chicken

CA21CH-0341-5-1	CA21CH-0341-5-1	SRR26215154	SAMN37599004	PRJNA1022324	*Campylobacter coli*	Retail chicken
CA21CH-0341-5-2	CA21CH-0341-5-2	SRR26215153	SAMN37599005	PRJNA1022324	*Campylobacter coli*	Retail chicken
CA21CH-0341-5-3	CA21CH-0341-5-3	SRR26215152	SAMN37599006	PRJNA1022324	*Campylobacter coli*	Retail chicken
CA21CH-0341-5-4	CA21CH-0341-5-4	SRR26215150	SAMN37599007	PRJNA1022324	*Campylobacter coli*	Retail chicken
CA21CH-0341-5-5	CA21CH-0341-5-5	SRR26215149	SAMN37599008	PRJNA1022324	*Campylobacter coli*	Retail chicken
CA21CH-0341-5-6	CA21CH-0341-5-6	SRR26215148	SAMN37599009	PRJNA1022324	*Campylobacter coli*	Retail chicken
CA21CH-0341-5-7	CA21CH-0341-5-7	SRR26215147	SAMN37599010	PRJNA1022324	*Campylobacter coli*	Retail chicken
CA21CH-0341-5-8	CA21CH-0341-5-8	SRR26215146	SAMN37599011	PRJNA1022324	*Campylobacter coli*	Retail chicken
CA21CH-0341-6-1	CA21CH-0341-6-1	SRR26215145	SAMN37599012	PRJNA1022324	*Campylobacter coli*	Retail chicken
CA21CH-0341-6-2	CA21CH-0341-6-2	SRR26215144	SAMN37599013	PRJNA1022324	*Campylobacter coli*	Retail chicken
CA21CH-0341-6-3	CA21CH-0341-6-3	SRR26215143	SAMN37599014	PRJNA1022324	*Campylobacter coli*	Retail chicken
CA21CH-0341-6-4	CA21CH-0341-6-4	SRR26215142	SAMN37599015	PRJNA1022324	*Campylobacter coli*	Retail chicken
CA21CH-0341-6-5	CA21CH-0341-6-5	SRR26215141	SAMN37599016	PRJNA1022324	*Campylobacter coli*	Retail chicken
CA21CH-0341-6-6	CA21CH-0341-6-6	SRR26215139	SAMN37599017	PRJNA1022324	*Campylobacter coli*	Retail chicken
CA21CH-0341-6-7	CA21CH-0341-6-7	SRR26215138	SAMN37599018	PRJNA1022324	*Campylobacter jejuni*	Retail chicken
CA21CH-0341-6-8	CA21CH-0341-6-8	SRR26215137	SAMN37599019	PRJNA1022324	*Campylobacter coli*	Retail chicken
CA21CH-0347-3-1	CA21CH-0347-3-1	SRR26215136	SAMN37599020	PRJNA1022324	*Campylobacter jejuni*	Retail chicken
CA21CH-0347-3-2	CA21CH-0347-3-2	SRR26215135	SAMN37599021	PRJNA1022324	*Campylobacter jejuni*	Retail chicken
CA21CH-0347-3-3	CA21CH-0347-3-3	SRR26215134	SAMN37599022	PRJNA1022324	*Campylobacter jejuni*	Retail chicken
CA21CH-0347-5-1	CA21CH-0347-5-1	SRR26215133	SAMN37599023	PRJNA1022324	*Campylobacter jejuni*	Retail chicken
CA21CH-0347-5-2	CA21CH-0347-5-2	SRR26215132	SAMN37599024	PRJNA1022324	*Campylobacter jejuni*	Retail chicken
CA21CH-0347-5-3	CA21CH-0347-5-3	SRR26215131	SAMN37599025	PRJNA1022324	*Campylobacter jejuni*	Retail chicken
CA21CH-0347-5-4	CA21CH-0347-5-4	SRR26215130	SAMN37599026	PRJNA1022324	*Campylobacter jejuni*	Retail chicken
CA21CH-0347-5-5	CA21CH-0347-5-5	SRR26215041	SAMN37599027	PRJNA1022324	*Campylobacter jejuni*	Retail chicken
CA21CH-0347-5-6	CA21CH-0347-5-6	SRR26215040	SAMN37599028	PRJNA1022324	*Campylobacter jejuni*	Retail chicken
CA21CH-0347-6-1	CA21CH-0347-6-1	SRR26215039	SAMN37599029	PRJNA1022324	*Campylobacter jejuni*	Retail chicken
CA21CH-0347-6-2	CA21CH-0347-6-2	SRR26215038	SAMN37599030	PRJNA1022324	*Campylobacter jejuni*	Retail chicken
CA21CH-0347-6-3	CA21CH-0347-6-3	SRR26215037	SAMN37599031	PRJNA1022324	*Campylobacter jejuni*	Retail chicken
CA21CH-0347-6-4	CA21CH-0347-6-4	SRR26215036	SAMN37599032	PRJNA1022324	*Campylobacter jejuni*	Retail chicken
CA21CH-0347-6-5	CA21CH-0347-6-5	SRR26215035	SAMN37599033	PRJNA1022324	*Campylobacter jejuni*	Retail chicken
CA21CH-0347-6-6	CA21CH-0347-6-6	SRR26215034	SAMN37599034	PRJNA1022324	*Campylobacter jejuni*	Retail chicken
CA21CH-0348-6-1	CA21CH-0348-6-1	SRR26215033	SAMN37599035	PRJNA1022324	*Campylobacter coli*	Retail chicken
CA21CH-0348-6-2	CA21CH-0348-6-2	SRR26215032	SAMN37599036	PRJNA1022324	*Campylobacter coli*	Retail chicken
CA21CH-0348-6-3	CA21CH-0348-6-3	SRR26215030	SAMN37599037	PRJNA1022324	*Campylobacter coli*	Retail chicken
CA21CH-0348-6-4	CA21CH-0348-6-4	SRR26215029	SAMN37599038	PRJNA1022324	*Campylobacter coli*	Retail chicken
CA21CH-0348-6-5	CA21CH-0348-6-5	SRR26215028	SAMN37599039	PRJNA1022324	*Campylobacter coli*	Retail chicken
CA21CH-0348-6-6	CA21CH-0348-6-6	SRR26215027	SAMN37599040	PRJNA1022324	*Campylobacter coli*	Retail chicken
CA21CH-0349-2-1	CA21CH-0349-2-1	SRR26215026	SAMN37599041	PRJNA1022324	*Campylobacter coli*	Retail chicken
CA21CH-0349-2-2	CA21CH-0349-2-2	SRR26215025	SAMN37599042	PRJNA1022324	*Campylobacter coli*	Retail chicken
CA21CH-0349-2-3	CA21CH-0349-2-3	SRR26215024	SAMN37599043	PRJNA1022324	*Campylobacter coli*	Retail chicken

CA21CH-0349-2-4	CA21CH-0349-2-4	SRR26215023	SAMN37599044	PRJNA1022324	*Campylobacter coli*	Retail chicken
CA21CH-0349-2-5	CA21CH-0349-2-5	SRR26215022	SAMN37599045	PRJNA1022324	*Campylobacter coli*	Retail chicken
CA21CH-0349-2-6	CA21CH-0349-2-6	SRR26215021	SAMN37599046	PRJNA1022324	*Campylobacter coli*	Retail chicken
CA21CH-0349-5-1	CA21CH-0349-5-1	SRR26215019	SAMN37599047	PRJNA1022324	*Campylobacter jejuni*	Retail chicken
CA21CH-0349-5-2	CA21CH-0349-5-2	SRR26215018	SAMN37599048	PRJNA1022324	*Campylobacter jejuni*	Retail chicken
CA21CH-0349-5-3	CA21CH-0349-5-3	SRR26215017	SAMN37599049	PRJNA1022324	*Campylobacter jejuni*	Retail chicken
CA21CH-0349-5-4	CA21CH-0349-5-4	SRR26215016	SAMN37599050	PRJNA1022324	*Campylobacter jejuni*	Retail chicken
CA21CH-0349-5-5	CA21CH-0349-5-5	SRR26215015	SAMN37599051	PRJNA1022324	*Campylobacter jejuni*	Retail chicken
CA21CH-0349-5-6	CA21CH-0349-5-6	SRR26215014	SAMN37599052	PRJNA1022324	*Campylobacter jejuni*	Retail chicken
CA21CH-0349-6-1	CA21CH-0349-6-1	SRR26215013	SAMN37599053	PRJNA1022324	*Campylobacter coli*	Retail chicken
CA21CH-0349-6-2	CA21CH-0349-6-2	SRR26215012	SAMN37599054	PRJNA1022324	*Campylobacter coli*	Retail chicken
CA21CH-0349-6-3	CA21CH-0349-6-3	SRR26215011	SAMN37599055	PRJNA1022324	*Campylobacter coli*	Retail chicken
CA21CH-0349-6-4	CA21CH-0349-6-4	SRR26215305	SAMN37599056	PRJNA1022324	*Campylobacter coli*	Retail chicken
CA21CH-0349-6-5	CA21CH-0349-6-5	SRR26215303	SAMN37599057	PRJNA1022324	*Campylobacter coli*	Retail chicken
CA21CH-0349-6-6	CA21CH-0349-6-6	SRR26215302	SAMN37599058	PRJNA1022324	*Campylobacter coli*	Retail chicken
CA21CH-0350-2-1	CA21CH-0350-2-1	SRR26215301	SAMN37599059	PRJNA1022324	*Campylobacter jejuni*	Retail chicken
CA21CH-0350-2-2	CA21CH-0350-2-2	SRR26215300	SAMN37599060	PRJNA1022324	*Campylobacter jejuni*	Retail chicken
CA21CH-0350-2-3	CA21CH-0350-2-3	SRR26215299	SAMN37599061	PRJNA1022324	*Campylobacter jejuni*	Retail chicken
CA21CH-0350-2-4	CA21CH-0350-2-4	SRR26215298	SAMN37599062	PRJNA1022324	*Campylobacter jejuni*	Retail chicken
CA21CH-0350-2-5	CA21CH-0350-2-5	SRR26215297	SAMN37599063	PRJNA1022324	*Campylobacter jejuni*	Retail chicken
CA21CH-0350-2-6	CA21CH-0350-2-6	SRR26215296	SAMN37599064	PRJNA1022324	*Campylobacter jejuni*	Retail chicken
CA21CH-0350-3-1	CA21CH-0350-3-1	SRR26215295	SAMN37599065	PRJNA1022324	*Campylobacter jejuni*	Retail chicken
CA21CH-0350-3-2	CA21CH-0350-3-2	SRR26215294	SAMN37599066	PRJNA1022324	*Campylobacter jejuni*	Retail chicken
CA21CH-0350-3-3	CA21CH-0350-3-3	SRR26215291	SAMN37599067	PRJNA1022324	*Campylobacter jejuni*	Retail chicken
CA21CH-0350-5-1	CA21CH-0350-5-1	SRR26215290	SAMN37599068	PRJNA1022324	*Campylobacter jejuni*	Retail chicken
CA21CH-0350-5-2	CA21CH-0350-5-2	SRR26215289	SAMN37599069	PRJNA1022324	*Campylobacter jejuni*	Retail chicken
CA21CH-0350-5-3	CA21CH-0350-5-3	SRR26215288	SAMN37599070	PRJNA1022324	*Campylobacter jejuni*	Retail chicken
CA21CH-0350-5-4	CA21CH-0350-5-4	SRR26215287	SAMN37599071	PRJNA1022324	*Campylobacter jejuni*	Retail chicken
CA21CH-0350-5-5	CA21CH-0350-5-5	SRR26215286	SAMN37599072	PRJNA1022324	*Campylobacter jejuni*	Retail chicken
CA21CH-0350-5-6	CA21CH-0350-5-6	SRR26215285	SAMN37599073	PRJNA1022324	*Campylobacter jejuni*	Retail chicken
CA21CH-0350-6-1	CA21CH-0350-6-1	SRR26215284	SAMN37599074	PRJNA1022324	*Campylobacter jejuni*	Retail chicken
CA21CH-0350-6-2	CA21CH-0350-6-2	SRR26215283	SAMN37599075	PRJNA1022324	*Campylobacter jejuni*	Retail chicken
CA21CH-0350-6-3	CA21CH-0350-6-3	SRR26215282	SAMN37599076	PRJNA1022324	*Campylobacter jejuni*	Retail chicken
CA21CH-0350-6-4	CA21CH-0350-6-4	SRR26215280	SAMN37599077	PRJNA1022324	*Campylobacter jejuni*	Retail chicken
CA21CH-0350-6-5	CA21CH-0350-6-5	SRR26215279	SAMN37599078	PRJNA1022324	*Campylobacter jejuni*	Retail chicken
CA21CH-0350-6-6	CA21CH-0350-6-6	SRR26215278	SAMN37599079	PRJNA1022324	*Campylobacter jejuni*	Retail chicken
CA21CH-0351-1-1	CA21CH-0351-1-1	SRR26215277	SAMN37599080	PRJNA1022324	*Campylobacter coli*	Retail chicken
CA21CH-0351-2-1	CA21CH-0351-2-1	SRR26215276	SAMN37599081	PRJNA1022324	*Campylobacter jejuni*	Retail chicken
CA21CH-0351-2-2	CA21CH-0351-2-2	SRR26215275	SAMN37599082	PRJNA1022324	*Campylobacter coli*	Retail chicken
CA21CH-0351-2-3	CA21CH-0351-2-3	SRR26215274	SAMN37599083	PRJNA1022324	*Campylobacter jejuni*	Retail chicken

CA21CH-0351-2-4	CA21CH-0351-2-4	SRR26214850	SAMN37599084	PRJNA1022324	*Campylobacter jejuni*	Retail chicken
CA21CH-0351-2-5	CA21CH-0351-2-5	SRR26214849	SAMN37599085	PRJNA1022324	*Campylobacter jejuni*	Retail chicken
CA21CH-0351-2-6	CA21CH-0351-2-6	SRR26214848	SAMN37599086	PRJNA1022324	*Campylobacter jejuni*	Retail chicken
CA21CH-0351-3-1	CA21CH-0351-3-1	SRR26214846	SAMN37599087	PRJNA1022324	*Campylobacter coli*	Retail chicken
CA21CH-0351-5-1	CA21CH-0351-5-1	SRR26214845	SAMN37599088	PRJNA1022324	*Campylobacter coli*	Retail chicken
CA21CH-0351-5-2	CA21CH-0351-5-2	SRR26214844	SAMN37599089	PRJNA1022324	*Campylobacter coli*	Retail chicken
CA21CH-0351-5-3	CA21CH-0351-5-3	SRR26214843	SAMN37599090	PRJNA1022324	*Campylobacter coli*	Retail chicken
CA21CH-0351-5-4	CA21CH-0351-5-4	SRR26214842	SAMN37599091	PRJNA1022324	*Campylobacter coli*	Retail chicken
CA21CH-0351-5-5	CA21CH-0351-5-5	SRR26214841	SAMN37599092	PRJNA1022324	*Campylobacter coli*	Retail chicken
CA21CH-0351-5-6	CA21CH-0351-5-6	SRR26214840	SAMN37599093	PRJNA1022324	*Campylobacter coli*	Retail chicken
CA21CH-0351-6-1	CA21CH-0351-6-1	SRR26214839	SAMN37599094	PRJNA1022324	*Campylobacter coli*	Retail chicken
CA21CH-0351-6-2	CA21CH-0351-6-2	SRR26214838	SAMN37599095	PRJNA1022324	*Campylobacter coli*	Retail chicken
CA21CH-0351-6-3	CA21CH-0351-6-3	SRR26214837	SAMN37599096	PRJNA1022324	*Campylobacter coli*	Retail chicken
CA21CH-0353-2-1	CA21CH-0353-2-1	SRR26214835	SAMN37599097	PRJNA1022324	*Campylobacter jejuni*	Retail chicken
CA21CH-0353-2-2	CA21CH-0353-2-2	SRR26214834	SAMN37599098	PRJNA1022324	*Campylobacter jejuni*	Retail chicken
CA21CH-0353-2-3	CA21CH-0353-2-3	SRR26214833	SAMN37599099	PRJNA1022324	*Campylobacter jejuni*	Retail chicken
CA21CH-0353-2-4	CA21CH-0353-2-4	SRR26214832	SAMN37599100	PRJNA1022324	*Campylobacter jejuni*	Retail chicken
CA21CH-0353-2-5	CA21CH-0353-2-5	SRR26214831	SAMN37599101	PRJNA1022324	*Campylobacter jejuni*	Retail chicken
CA21CH-0353-2-6	CA21CH-0353-2-6	SRR26214830	SAMN37599102	PRJNA1022324	*Campylobacter jejuni*	Retail chicken
CA21CH-0353-3-1	CA21CH-0353-3-1	SRR26214829	SAMN37599103	PRJNA1022324	*Campylobacter jejuni*	Retail chicken
CA21CH-0353-3-2	CA21CH-0353-3-2	SRR26214828	SAMN37599104	PRJNA1022324	*Campylobacter jejuni*	Retail chicken
CA21CH-0353-3-3	CA21CH-0353-3-3	SRR26214827	SAMN37599105	PRJNA1022324	*Campylobacter jejuni*	Retail chicken
CA21CH-0353-3-4	CA21CH-0353-3-4	SRR26214826	SAMN37599106	PRJNA1022324	*Campylobacter jejuni*	Retail chicken
CA21CH-0353-3-5	CA21CH-0353-3-5	SRR26214824	SAMN37599107	PRJNA1022324	*Campylobacter jejuni*	Retail chicken
CA21CH-0353-3-6	CA21CH-0353-3-6	SRR26214823	SAMN37599108	PRJNA1022324	*Campylobacter jejuni*	Retail chicken
CA21CH-0353-5-1	CA21CH-0353-5-1	SRR26214822	SAMN37599109	PRJNA1022324	*Campylobacter coli*	Retail chicken
CA21CH-0353-5-2	CA21CH-0353-5-2	SRR26214821	SAMN37599110	PRJNA1022324	*Campylobacter coli*	Retail chicken
CA21CH-0353-5-3	CA21CH-0353-5-3	SRR26214820	SAMN37599111	PRJNA1022324	*Campylobacter coli*	Retail chicken
CA21CH-0353-5-4	CA21CH-0353-5-4	SRR26214819	SAMN37599112	PRJNA1022324	*Campylobacter coli*	Retail chicken
CA21CH-0353-5-5	CA21CH-0353-5-5	SRR26215010	SAMN37599113	PRJNA1022324	*Campylobacter coli*	Retail chicken
CA21CH-0353-5-6	CA21CH-0353-5-6	SRR26215009	SAMN37599114	PRJNA1022324	*Campylobacter coli*	Retail chicken
CA21CH-0353-6-1	CA21CH-0353-6-1	SRR26215008	SAMN37599115	PRJNA1022324	*Campylobacter jejuni*	Retail chicken
CA21CH-0353-6-2	CA21CH-0353-6-2	SRR26215007	SAMN37599116	PRJNA1022324	*Campylobacter jejuni*	Retail chicken
CA21CH-0353-6-3	CA21CH-0353-6-3	SRR26215005	SAMN37599117	PRJNA1022324	*Campylobacter jejuni*	Retail chicken
CA21CH-0353-6-4	CA21CH-0353-6-4	SRR26215004	SAMN37599118	PRJNA1022324	*Campylobacter jejuni*	Retail chicken
CA21CH-0353-6-5	CA21CH-0353-6-5	SRR26215003	SAMN37599119	PRJNA1022324	*Campylobacter jejuni*	Retail chicken
CA21CH-0353-6-6	CA21CH-0353-6-6	SRR26215002	SAMN37599120	PRJNA1022324	*Campylobacter jejuni*	Retail chicken
CA21CH-0355-3-1	CA21CH-0355-3-1	SRR26215001	SAMN37599121	PRJNA1022324	*Campylobacter jejuni*	Retail chicken
CA21CH-0355-3-2	CA21CH-0355-3-2	SRR26215000	SAMN37599122	PRJNA1022324	*Campylobacter jejuni*	Retail chicken
CA21CH-0355-3-3	CA21CH-0355-3-3	SRR26214999	SAMN37599123	PRJNA1022324	*Campylobacter jejuni*	Retail chicken

CA21CH-0355-5-1	CA21CH-0355-5-1	SRR26214998	SAMN37599124	PRJNA1022324	*Campylobacter jejuni*	Retail chicken
CA21CH-0355-5-2	CA21CH-0355-5-2	SRR26214997	SAMN37599125	PRJNA1022324	*Campylobacter jejuni*	Retail chicken
CA21CH-0355-5-3	CA21CH-0355-5-3	SRR26214996	SAMN37599126	PRJNA1022324	*Campylobacter jejuni*	Retail chicken
CA21CH-0355-5-4	CA21CH-0355-5-4	SRR26214994	SAMN37599127	PRJNA1022324	*Campylobacter jejuni*	Retail chicken
CA21CH-0355-5-5	CA21CH-0355-5-5	SRR26214993	SAMN37599128	PRJNA1022324	*Campylobacter jejuni*	Retail chicken
CA21CH-0355-5-6	CA21CH-0355-5-6	SRR26214992	SAMN37599129	PRJNA1022324	*Campylobacter jejuni*	Retail chicken
CA21CH-0356-6-1	CA21CH-0356-6-1	SRR26214991	SAMN37599130	PRJNA1022324	*Campylobacter jejuni*	Retail chicken
CA21CH-0356-6-2	CA21CH-0356-6-2	SRR26214990	SAMN37599131	PRJNA1022324	*Campylobacter jejuni*	Retail chicken
CA21CH-0356-6-3	CA21CH-0356-6-3	SRR26214989	SAMN37599132	PRJNA1022324	*Campylobacter jejuni*	Retail chicken
CA21CH-0356-6-4	CA21CH-0356-6-4	SRR26214988	SAMN37599133	PRJNA1022324	*Campylobacter jejuni*	Retail chicken
CA21CH-0356-6-5	CA21CH-0356-6-5	SRR26214987	SAMN37599134	PRJNA1022324	*Campylobacter jejuni*	Retail chicken
CA21CH-0356-6-6	CA21CH-0356-6-6	SRR26214986	SAMN37599135	PRJNA1022324	*Campylobacter jejuni*	Retail chicken
CA21CH-0357-2-1	CA21CH-0357-2-1	SRR26214985	SAMN37599136	PRJNA1022324	*Campylobacter jejuni*	Retail chicken
CA21CH-0357-2-2	CA21CH-0357-2-2	SRR26214983	SAMN37599137	PRJNA1022324	*Campylobacter jejuni*	Retail chicken
CA21CH-0357-2-3	CA21CH-0357-2-3	SRR26214982	SAMN37599138	PRJNA1022324	*Campylobacter jejuni*	Retail chicken
CA21CH-0357-2-4	CA21CH-0357-2-4	SRR26214981	SAMN37599139	PRJNA1022324	*Campylobacter jejuni*	Retail chicken
CA21CH-0357-2-5	CA21CH-0357-2-5	SRR26214980	SAMN37599140	PRJNA1022324	*Campylobacter jejuni*	Retail chicken
CA21CH-0357-2-6	CA21CH-0357-2-6	SRR26214979	SAMN37599141	PRJNA1022324	*Campylobacter jejuni*	Retail chicken
CA21CH-0357-3-1	CA21CH-0357-3-1	SRR26215273	SAMN37599142	PRJNA1022324	*Campylobacter jejuni*	Retail chicken
CA21CH-0357-3-2	CA21CH-0357-3-2	SRR26215272	SAMN37599143	PRJNA1022324	*Campylobacter jejuni*	Retail chicken
CA21CH-0357-3-3	CA21CH-0357-3-3	SRR26215271	SAMN37599144	PRJNA1022324	*Campylobacter jejuni*	Retail chicken
CA21CH-0357-5-1	CA21CH-0357-5-1	SRR26215270	SAMN37599145	PRJNA1022324	*Campylobacter jejuni*	Retail chicken
CA21CH-0357-5-2	CA21CH-0357-5-2	SRR26215269	SAMN37599146	PRJNA1022324	*Campylobacter jejuni*	Retail chicken
CA21CH-0357-5-3	CA21CH-0357-5-3	SRR26215267	SAMN37599147	PRJNA1022324	*Campylobacter jejuni*	Retail chicken
CA21CH-0357-5-4	CA21CH-0357-5-4	SRR26215266	SAMN37599148	PRJNA1022324	*Campylobacter jejuni*	Retail chicken
CA21CH-0357-5-5	CA21CH-0357-5-5	SRR26215265	SAMN37599149	PRJNA1022324	*Campylobacter jejuni*	Retail chicken
CA21CH-0357-5-6	CA21CH-0357-5-6	SRR26215264	SAMN37599150	PRJNA1022324	*Campylobacter jejuni*	Retail chicken
CA21CH-0357-6-1	CA21CH-0357-6-1	SRR26215263	SAMN37599151	PRJNA1022324	*Campylobacter jejuni*	Retail chicken
CA21CH-0357-6-2	CA21CH-0357-6-2	SRR26215262	SAMN37599152	PRJNA1022324	*Campylobacter jejuni*	Retail chicken
CA21CH-0357-6-3	CA21CH-0357-6-3	SRR26215261	SAMN37599153	PRJNA1022324	*Campylobacter jejuni*	Retail chicken
CA21CH-0357-6-4	CA21CH-0357-6-4	SRR26215260	SAMN37599154	PRJNA1022324	*Campylobacter jejuni*	Retail chicken
CA21CH-0357-6-5	CA21CH-0357-6-5	SRR26215259	SAMN37599155	PRJNA1022324	*Campylobacter jejuni*	Retail chicken
CA21CH-0357-6-6	CA21CH-0357-6-6	SRR26215258	SAMN37599156	PRJNA1022324	*Campylobacter jejuni*	Retail chicken
CA21CH-0358-5-1	CA21CH-0358-5-1	SRR26215256	SAMN37599157	PRJNA1022324	*Campylobacter jejuni*	Retail chicken
CA21CH-0358-5-2	CA21CH-0358-5-2	SRR26215255	SAMN37599158	PRJNA1022324	*Campylobacter jejuni*	Retail chicken
CA21CH-0358-5-3	CA21CH-0358-5-3	SRR26215254	SAMN37599159	PRJNA1022324	*Campylobacter jejuni*	Retail chicken
CA21CH-0358-5-4	CA21CH-0358-5-4	SRR26215253	SAMN37599160	PRJNA1022324	*Campylobacter jejuni*	Retail chicken
CA21CH-0358-5-5	CA21CH-0358-5-5	SRR26215252	SAMN37599161	PRJNA1022324	*Campylobacter jejuni*	Retail chicken
CA21CH-0358-5-6	CA21CH-0358-5-6	SRR26215251	SAMN37599162	PRJNA1022324	*Campylobacter jejuni*	Retail chicken
CA21CH-0359-2-1	CA21CH-0359-2-1	SRR26215250	SAMN37599163	PRJNA1022324	*Campylobacter coli*	Retail chicken

CA21CH-0359-2-2	CA21CH-0359-2-2	SRR26215249	SAMN37599164	PRJNA1022324	*Campylobacter jejuni*	Retail chicken
CA21CH-0359-2-3	CA21CH-0359-2-3	SRR26215248	SAMN37599165	PRJNA1022324	*Campylobacter coli*	Retail chicken
CA21CH-0359-2-4	CA21CH-0359-2-4	SRR26215247	SAMN37599166	PRJNA1022324	*Campylobacter jejuni*	Retail chicken
CA21CH-0359-2-5	CA21CH-0359-2-5	SRR26215244	SAMN37599167	PRJNA1022324	*Campylobacter coli*	Retail chicken
CA21CH-0359-2-6	CA21CH-0359-2-6	SRR26215243	SAMN37599168	PRJNA1022324	*Campylobacter coli*	Retail chicken
CA21CH-0359-3-1	CA21CH-0359-3-1	SRR26215242	SAMN37599169	PRJNA1022324	*Campylobacter coli*	Retail chicken
CA21CH-0359-3-2	CA21CH-0359-3-2	SRR26215129	SAMN37599170	PRJNA1022324	*Campylobacter jejuni*	Retail chicken
CA21CH-0359-3-3	CA21CH-0359-3-3	SRR26215128	SAMN37599171	PRJNA1022324	*Campylobacter coli*	Retail chicken
CA21CH-0359-3-4	CA21CH-0359-3-4	SRR26215127	SAMN37599172	PRJNA1022324	*Campylobacter coli*	Retail chicken
CA21CH-0359-3-5	CA21CH-0359-3-5	SRR26215126	SAMN37599173	PRJNA1022324	*Campylobacter coli*	Retail chicken
CA21CH-0359-3-6	CA21CH-0359-3-6	SRR26215125	SAMN37599174	PRJNA1022324	*Campylobacter coli*	Retail chicken
CA21CH-0359-5-1	CA21CH-0359-5-1	SRR26215124	SAMN37599175	PRJNA1022324	*Campylobacter coli*	Retail chicken
CA21CH-0359-5-2	CA21CH-0359-5-2	SRR26215123	SAMN37599176	PRJNA1022324	*Campylobacter coli*	Retail chicken
CA21CH-0359-5-3	CA21CH-0359-5-3	SRR26215121	SAMN37599177	PRJNA1022324	*Campylobacter coli*	Retail chicken
CA21CH-0359-5-4	CA21CH-0359-5-4	SRR26215120	SAMN37599178	PRJNA1022324	*Campylobacter coli*	Retail chicken
CA21CH-0359-5-5	CA21CH-0359-5-5	SRR26215119	SAMN37599179	PRJNA1022324	*Campylobacter coli*	Retail chicken
CA21CH-0359-5-6	CA21CH-0359-5-6	SRR26215118	SAMN37599180	PRJNA1022324	*Campylobacter coli*	Retail chicken
CA21CH-0359-6-1	CA21CH-0359-6-1	SRR26215117	SAMN37599181	PRJNA1022324	*Campylobacter coli*	Retail chicken
CA21CH-0359-6-2	CA21CH-0359-6-2	SRR26215116	SAMN37599182	PRJNA1022324	*Campylobacter coli*	Retail chicken
CA21CH-0359-6-3	CA21CH-0359-6-3	SRR26215115	SAMN37599183	PRJNA1022324	*Campylobacter coli*	Retail chicken
CA21CH-0359-6-4	CA21CH-0359-6-4	SRR26215114	SAMN37599184	PRJNA1022324	*Campylobacter coli*	Retail chicken
CA21CH-0359-6-5	CA21CH-0359-6-5	SRR26215113	SAMN37599185	PRJNA1022324	*Campylobacter coli*	Retail chicken
CA21CH-0361-2-1	CA21CH-0361-2-1	SRR26215112	SAMN37599186	PRJNA1022324	*Campylobacter jejuni*	Retail chicken
CA21CH-0361-2-2	CA21CH-0361-2-2	SRR26215110	SAMN37599187	PRJNA1022324	*Campylobacter jejuni*	Retail chicken
CA21CH-0361-2-3	CA21CH-0361-2-3	SRR26215109	SAMN37599188	PRJNA1022324	*Campylobacter jejuni*	Retail chicken
CA21CH-0361-2-4	CA21CH-0361-2-4	SRR26215108	SAMN37599189	PRJNA1022324	*Campylobacter jejuni*	Retail chicken
CA21CH-0361-2-5	CA21CH-0361-2-5	SRR26215107	SAMN37599190	PRJNA1022324	*Campylobacter jejuni*	Retail chicken
CA21CH-0361-2-6	CA21CH-0361-2-6	SRR26215106	SAMN37599191	PRJNA1022324	*Campylobacter jejuni*	Retail chicken
CA21CH-0361-3-1	CA21CH-0361-3-1	SRR26215105	SAMN37599192	PRJNA1022324	*Campylobacter coli*	Retail chicken
CA21CH-0361-3-2	CA21CH-0361-3-2	SRR26215104	SAMN37599193	PRJNA1022324	*Campylobacter coli*	Retail chicken
CA21CH-0361-3-3	CA21CH-0361-3-3	SRR26215103	SAMN37599194	PRJNA1022324	*Campylobacter coli*	Retail chicken
CA21CH-0361-3-4	CA21CH-0361-3-4	SRR26215102	SAMN37599195	PRJNA1022324	*Campylobacter coli*	Retail chicken
CA21CH-0361-3-5	CA21CH-0361-3-5	SRR26215101	SAMN37599196	PRJNA1022324	*Campylobacter coli*	Retail chicken
CA21CH-0361-3-6	CA21CH-0361-3-6	SRR26215099	SAMN37599197	PRJNA1022324	*Campylobacter coli*	Retail chicken
CA21CH-0361-5-1	CA21CH-0361-5-1	SRR26215098	SAMN37599198	PRJNA1022324	*Campylobacter jejuni*	Retail chicken
CA21CH-0361-5-2	CA21CH-0361-5-2	SRR26215241	SAMN37599199	PRJNA1022324	*Campylobacter jejuni*	Retail chicken
CA21CH-0361-5-3	CA21CH-0361-5-3	SRR26215240	SAMN37599200	PRJNA1022324	*Campylobacter jejuni*	Retail chicken
CA21CH-0361-5-4	CA21CH-0361-5-4	SRR26215239	SAMN37599201	PRJNA1022324	*Campylobacter jejuni*	Retail chicken
CA21CH-0361-5-5	CA21CH-0361-5-5	SRR26215238	SAMN37599202	PRJNA1022324	*Campylobacter jejuni*	Retail chicken
CA21CH-0361-5-6	CA21CH-0361-5-6	SRR26215237	SAMN37599203	PRJNA1022324	*Campylobacter jejuni*	Retail chicken

CA21CH-0361-6-1	CA21CH-0361-6-1	SRR26215236	SAMN37599204	PRJNA1022324	*Campylobacter jejuni*	Retail chicken
CA21CH-0361-6-2	CA21CH-0361-6-2	SRR26215235	SAMN37599205	PRJNA1022324	*Campylobacter jejuni*	Retail chicken
CA21CH-0361-6-3	CA21CH-0361-6-3	SRR26215234	SAMN37599206	PRJNA1022324	*Campylobacter jejuni*	Retail chicken
CA21CH-0361-6-4	CA21CH-0361-6-4	SRR26215232	SAMN37599207	PRJNA1022324	*Campylobacter jejuni*	Retail chicken
CA21CH-0361-6-5	CA21CH-0361-6-5	SRR26215231	SAMN37599208	PRJNA1022324	*Campylobacter jejuni*	Retail chicken
CA21CH-0361-6-6	CA21CH-0361-6-6	SRR26215230	SAMN37599209	PRJNA1022324	*Campylobacter jejuni*	Retail chicken

Supplementary Method 2: Read prefiltering, assembly and quality control

The raw Illumina paired reads were trimmed in Galaxy using fastp v0.19.5+galaxy1 [1]. The SPAdes assembler was used for contig assembly with Shovill v1.1.0+galaxy0 (https://github.com/tseemann/shovill), with an estimated genome size of 2,000,000 and minimum coverage to call part of a contig set to 0 (AUTO). The quality of the assemblies was assessed with QUAST v5.0.2 [2] and CheckM v1.0.11 [3] and coverage estimated using BWA-MEM Galaxy v0.7.17.1 [4] and CoverM v0.3.2 (https://github.com/wwood/CoverM). Genomes that appeared mixed were re-sequenced.

Supplementary Method 3: Additional antimicrobial resistance determinant screening

For quinolone resistance mutations in *gyrA*, hits were verified using starAMR v0.5.1 [5] with the PointFinder database [6] using 98% BLAST identity and 95% PointFinder BLAST overlap thresholds. If starAMR did not identify the mutation, the ARIBA results were manually screened to confirm presence of nonsynonymous mutations (T86I and T86I+P104S). T86I mutations have been extensively reported and verified to confer quinolone resistance [7], with double mutations (T86I and P104S) also observed in resistant isolates [8].

For 23S rRNA gene mutations conferring resistance to macrolides, the ARIBA results were summarised with ARIBA summary to identify any mutations at the 2074 and 2075 position in the *C. jejuni* 23S rRNA gene, 2075 or 2076 in the *C. lari* gene and 2232 or 2233 in the *C. coli* gene, in cases where any of these assembled, regardless of the species of the genome. In cases where none of the genes assembled, the genome assemblies were annotated with Bakta v1.7.1+galaxy0 [9] to manually extract the nucleotide sequences of the 23S rRNA gene. The genes were aligned to the references using muscle v3.8.31 in SeaView v5.0.4 [10–12] for manual screening of mutations at these positions.

Supplementary Method 4: Culturing procedure for isolates representing STs identified with Bolton broth enrichment or CAT broth enrichment only

The isolates were cultured from glycerol stock on Columbia blood agar (CBA) at 37°C for 48 hours in microaerophilic conditions, using an anaerobic cabinet (Don Whitley Scientific). For each isolate, an initial inoculum equivalent to 0.5 McFarland was prepared in sterile phosphate buffered saline (PBS), determined using an optical density meter (Fisher Scientific Cell Density Meter Model 40, Loughborough, UK) value of 0.1 (±0.01).

For direct plating, approximate 1 in 100 serial dilutions were performed in PBS by adding 100 µL to 10 mL PBS and 100 µL of the approximate 10^{-4}, 10^{-6} and 10^{-8} dilutions were spread on four mCCDA and four CBA plates. Two plates of each type and each dilution were incubated at 37°C and the remaining plates at 42°C in microaerophilic conditions (85% nitrogen, 10% carbon dioxide and 5% oxygen) using an anaerobic cabinet (Don Whitley Scientific) and Anoxomat jars (Mart Microbiology B.V), respectively.

Two tissue flasks containing 20 mL Bolton broth and two tissue flasks containing 20 mL CAT broth were inoculated with 200 µL of the undiluted initial inoculum. One flask of each type was incubated at 37°C and the other at 42°C in microaerophilic conditions, as detailed previously. Following enrichment, approximate 1 in 100 serial dilutions were performed in PBS and 100 µL of the approximate 10^{-4}, 10^{-6} and 10^{-8} dilutions were spread on mCCDA and CBA in duplicate.

During the first trial involving isolate 330-6-5, the post-enrichment plates from the 42°C enrichments were also incubated at 42°C. Due to large amounts of swarming on the plates, the post-enrichment plates for the remaining isolates tested were incubated at 37°C instead.

Colonies on the mCCDA and CBA plates were counted after incubation. The average number of colonies, dilution factor and dilution volume spread on the plates were used to calculate the concentration of *Campylobacter* (cfu/mL). Due to 100 µL being added to 10 mL during the dilution series, the dilution factor for each dilution was slightly lower than 10^{-4}, 10^{-6} and 10^{-8}. However, 10^{-4}, 10^{-6} and 10^{-8} values were used in calculations. The slight variation would not significantly affect the cfu/mL values obtained, since the primary aim of this experiment was to evaluate growth or no growth in the tested conditions. The values were log-transformed for plotting and statistical analysis.

Table S3: Summary of the *Campylobacter* genomes obtained in this study, the samples they were obtained from, the culture method they were isolated with and their ST

Sample	Genome	Species	ST	Temperature	Broth	Plate
CH-0312	CA21CH-0312-6-1	*C. jejuni*	45	42	CAT	mCCDA
CH-0313	CA19CH-0313-3-1	*C. jejuni*	257	37	CAT	u-mCCDA
CH-0313	CA21CH-0313-3-2	*C. jejuni*	5136	37	CAT	u-mCCDA
CH-0313	CA21CH-0313-3-3	*C. jejuni*	257	37	CAT	u-mCCDA
CH-0313	CA21CH-0313-3-4	*C. jejuni*	257	37	CAT	u-mCCDA
CH-0313	CA21CH-0313-3-5	*C. jejuni*	257	37	CAT	u-mCCDA
CH-0313	CA21CH-0313-3-6	*C. jejuni*	257	37	CAT	u-mCCDA
CH-0313	CA21CH-0313-6-1	*C. jejuni*	5136	42	CAT	mCCDA
CH-0313	CA21CH-0313-6-2	*C. jejuni*	257	42	CAT	mCCDA
CH-0313	CA21CH-0313-6-3	*C. jejuni*	5136	42	CAT	u-mCCDA
CH-0313	CA21CH-0313-6-4	*C. jejuni*	5136	42	CAT	u-mCCDA
CH-0313	CA21CH-0313-6-5	*C. jejuni*	257	42	CAT	u-mCCDA
CH-0313	CA21CH-0313-6-6	*C. jejuni*	5136	42	CAT	u-mCCDA
CH-0314	CA21CH-0314-3-2	*C. jejuni*	464	37	CAT	mCCDA
CH-0314	CA21CH-0314-3-3	*C. jejuni*	6175	37	CAT	mCCDA
CH-0314	CA21CH-0314-3-4	*C. jejuni*	6175	37	CAT	mCCDA
CH-0314	CA21CH-0314-6-1R2	*C. jejuni*	464	42	CAT	u-mCCDA
CH-0314	CA21CH-0314-6-2R2	*C. jejuni*	464	42	CAT	u-mCCDA
CH-0314	CA21CH-0314-6-3R2	*C. jejuni*	464	42	CAT	u-mCCDA
CH-0315	CA21CH-0315-1-1	*C. jejuni*	cj unknown6	37	None (Direct plating)	mCCDA
CH-0315	CA21CH-0315-1-2-R2	*C. jejuni*	cj unknown6	37	None (Direct plating)	mCCDA
CH-0317	CA21CH-0317-1-1	*C. jejuni*	400	37	None (Direct plating)	mCCDA
CH-0317	CA21CH-0317-1-2	*C. jejuni*	400	37	None (Direct plating)	mCCDA
CH-0317	CA21CH-0317-1-3	*C. jejuni*	400	37	None (Direct plating)	mCCDA
CH-0317	CA21CH-0317-1-4	*C. jejuni*	400	37	None (Direct plating)	mCCDA
CH-0317	CA21CH-0317-1-5	*C. jejuni*	400	37	None (Direct plating)	u-mCCDA
CH-0317	CA21CH-0317-1-6	*C. jejuni*	400	37	None (Direct plating)	u-mCCDA
CH-0317	CA21CH-0317-1-7	*C. jejuni*	400	37	None (Direct plating)	u-mCCDA
CH-0317	CA21CH-0317-2-1-R	*C. jejuni*	400	37	Bolton	mCCDA
CH-0317	CA21CH-0317-2-2-R	*C. jejuni*	400	37	Bolton	mCCDA
CH-0317	CA21CH-0317-2-3	*C. jejuni*	400	37	Bolton	mCCDA
CH-0317	CA21CH-0317-2-4	*C. jejuni*	400	37	Bolton	mCCDA
CH-0317	CA21CH-0317-2-5	*C. jejuni*	400	37	Bolton	u-mCCDA
CH-0317	CA21CH-0317-2-6-R	*C. jejuni*	400	37	Bolton	u-mCCDA
CH-0317	CA21CH-0317-2-7-R	*C. jejuni*	400	37	Bolton	u-mCCDA
CH-0317	CA21CH-0317-2-8	*C. jejuni*	400	37	Bolton	u-mCCDA
CH-0317	CA21CH-0317-3-1	*C. jejuni*	400	37	CAT	mCCDA
CH-0317	CA21CH-0317-3-2	*C. jejuni*	400	37	CAT	mCCDA
CH-0317	CA21CH-0317-3-3	*C. jejuni*	400	37	CAT	mCCDA
CH-0317	CA21CH-0317-3-4	*C. jejuni*	400	37	CAT	mCCDA
CH-0317	CA21CH-0317-3-5	*C. jejuni*	400	37	CAT	u-mCCDA
CH-0317	CA21CH-0317-3-6	*C. jejuni*	400	37	CAT	u-mCCDA
CH-0317	CA21CH-0317-3-7	*C. jejuni*	400	37	CAT	u-mCCDA
CH-0317	CA21CH-0317-3-8	*C. jejuni*	400	37	CAT	u-mCCDA
CH-0317	CA21CH-0317-4-1	*C. jejuni*	400	42	None (Direct plating)	mCCDA
CH-0317	CA21CH-0317-4-2	*C. jejuni*	400	42	None (Direct plating)	mCCDA

CH-0317	CA21CH-0317-4-3	*C. jejuni*	400	42	None (Direct plating)	mCCDA
CH-0317	CA21CH-0317-4-4	*C. jejuni*	400	42	None (Direct plating)	mCCDA
CH-0317	CA21CH-0317-4-5	*C. jejuni*	400	42	None (Direct plating)	u-mCCDA
CH-0317	CA21CH-0317-4-6	*C. jejuni*	400	42	None (Direct plating)	u-mCCDA
CH-0317	CA21CH-0317-5-1	*C. jejuni*	400	42	Bolton	mCCDA
CH-0317	CA21CH-0317-5-2	*C. jejuni*	400	42	Bolton	mCCDA
CH-0317	CA21CH-0317-5-3	*C. jejuni*	400	42	Bolton	mCCDA
CH-0317	CA21CH-0317-5-4	*C. jejuni*	400	42	Bolton	mCCDA
CH-0317	CA21CH-0317-5-5	*C. jejuni*	400	42	Bolton	u-mCCDA
CH-0317	CA21CH-0317-5-6	*C. jejuni*	400	42	Bolton	u-mCCDA
CH-0317	CA21CH-0317-5-7	*C. jejuni*	400	42	Bolton	u-mCCDA
CH-0317	CA21CH-0317-5-8	*C. jejuni*	400	42	Bolton	u-mCCDA
CH-0317	CA21CH-0317-6-1	*C. jejuni*	400	42	CAT	mCCDA
CH-0317	CA21CH-0317-6-2	*C. jejuni*	400	42	CAT	mCCDA
CH-0317	CA21CH-0317-6-3	*C. jejuni*	400	42	CAT	mCCDA
CH-0317	CA21CH-0317-6-4	*C. jejuni*	400	42	CAT	mCCDA
CH-0317	CA21CH-0317-6-5	*C. jejuni*	400	42	CAT	u-mCCDA
CH-0317	CA21CH-0317-6-6	*C. jejuni*	400	42	CAT	u-mCCDA
CH-0317	CA21CH-0317-6-7	*C. jejuni*	400	42	CAT	u-mCCDA
CH-0317	CA21CH-0317-6-8	*C. jejuni*	400	42	CAT	u-mCCDA
CH-0318	CA21CH-0318-3-1	*C. jejuni*	5136	37	CAT	mCCDA
CH-0318	CA21CH-0318-3-2	*C. jejuni*	5136	37	CAT	mCCDA
CH-0318	CA21CH-0318-3-3	*C. jejuni*	5136	37	CAT	mCCDA
CH-0318	CA21CH-0318-3-4	*C. jejuni*	5136	37	CAT	mCCDA
CH-0318	CA21CH-0318-4-1	*C. jejuni*	5136	42	None (Direct plating)	mCCDA
CH-0318	CA21CH-0318-4-2	*C. jejuni*	5136	42	None (Direct plating)	mCCDA
CH-0318	CA21CH-0318-4-3	*C. jejuni*	5136	42	None (Direct plating)	mCCDA
CH-0318	CA21CH-0318-4-4	*C. jejuni*	5136	42	None (Direct plating)	mCCDA
CH-0319	CA21CH-0319-3-1	*C. jejuni*	6175	37	CAT	mCCDA
CH-0319	CA21CH-0319-3-2	*C. jejuni*	6175	37	CAT	mCCDA
CH-0319	CA21CH-0319-3-3	*C. jejuni*	6175	37	CAT	mCCDA
CH-0319	CA21CH-0319-3-4	*C. jejuni*	6175	37	CAT	mCCDA
CH-0319	CA21CH-0319-3-5	*C. jejuni*	6175	37	CAT	u-mCCDA
CH-0319	CA21CH-0319-3-6-R	*C. jejuni*	6175	37	CAT	u-mCCDA
CH-0319	CA21CH-0319-3-7-R	*C. jejuni*	6175	37	CAT	u-mCCDA
CH-0319	CA21CH-0319-3-8	*C. jejuni*	6175	37	CAT	u-mCCDA
CH-0319	CA21CH-0319-6-1	*C. jejuni*	6175	42	CAT	mCCDA
CH-0319	CA21CH-0319-6-2	*C. jejuni*	122	42	CAT	mCCDA
CH-0319	CA21CH-0319-6-3	*C. jejuni*	6175	42	CAT	mCCDA
CH-0319	CA21CH-0319-6-4	*C. jejuni*	6175	42	CAT	mCCDA
CH-0319	CA21CH-0319-6-5	*C. jejuni*	6175	42	CAT	u-mCCDA
CH-0319	CA21CH-0319-6-6	*C. jejuni*	6175	42	CAT	u-mCCDA
CH-0319	CA21CH-0319-6-7	*C. jejuni*	6175	42	CAT	u-mCCDA
CH-0319	CA21CH-0319-6-8	*C. jejuni*	6175	42	CAT	u-mCCDA
CH-0320	CA21CH-0320-2-1-R	*C. lari*	27	37	Bolton	mCCDA
CH-0320	CA21CH-0320-2-2-R	*C. lari*	27	37	Bolton	mCCDA
CH-0320	CA21CH-0320-2-3-R	*C. lari*	27	37	Bolton	mCCDA
CH-0320	CA21CH-0320-2-4-R	*C. lari*	27	37	Bolton	mCCDA
CH-0320	CA21CH-0320-2-5-R	*C. lari*	27	37	Bolton	u-mCCDA

CH-0320	CA21CH-0320-2-6-R	*C. lari*	27	37	Bolton	u-mCCDA
CH-0320	CA21CH-0320-2-7-R	*C. lari*	27	37	Bolton	u-mCCDA
CH-0320	CA21CH-0320-2-8-R	*C. lari*	27	37	Bolton	u-mCCDA
CH-0320	CA21CH-0320-3-1	*C. lari*	27	37	CAT	mCCDA
CH-0320	CA21CH-0320-3-2	*C. lari*	27	37	CAT	mCCDA
CH-0320	CA21CH-0320-3-3	*C. lari*	27	37	CAT	mCCDA
CH-0320	CA21CH-0320-3-4	*C. lari*	27	37	CAT	mCCDA
CH-0320	CA21CH-0320-3-5	*C. lari*	27	37	CAT	u-mCCDA
CH-0320	CA21CH-0320-3-6	*C. lari*	27	37	CAT	u-mCCDA
CH-0320	CA21CH-0320-3-7	*C. lari*	27	37	CAT	u-mCCDA
CH-0320	CA21CH-0320-3-8	*C. lari*	27	37	CAT	u-mCCDA
CH-0320	CA21CH-0320-6-1	*C. jejuni*	464	42	CAT	mCCDA
CH-0320	CA21CH-0320-6-2	*C. jejuni*	464	42	CAT	mCCDA
CH-0320	CA21CH-0320-6-3	*C. jejuni*	464	42	CAT	mCCDA
CH-0320	CA21CH-0320-6-4	*C. jejuni*	464	42	CAT	mCCDA
CH-0320	CA21CH-0320-6-5	*C. jejuni*	464	42	CAT	u-mCCDA
CH-0320	CA21CH-0320-6-6	*C. jejuni*	464	42	CAT	u-mCCDA
CH-0320	CA21CH-0320-6-7	*C. jejuni*	464	42	CAT	u-mCCDA
CH-0320	CA21CH-0320-6-8-R	*C. jejuni*	464	42	CAT	u-mCCDA
CH-0321	CA21CH-0321-3-1	*C. jejuni*	5136	37	CAT	mCCDA
CH-0321	CA21CH-0321-3-2	*C. jejuni*	5136	37	CAT	mCCDA
CH-0321	CA21CH-0321-3-3	*C. jejuni*	5136	37	CAT	mCCDA
CH-0321	CA21CH-0321-3-4	*C. jejuni*	5136	37	CAT	mCCDA
CH-0321	CA21CH-0321-4-1	*C. jejuni*	5136	42	None (Direct plating)	mCCDA
CH-0321	CA21CH-0321-4-2	*C. jejuni*	5136	42	None (Direct plating)	mCCDA
CH-0321	CA21CH-0321-6-1	*C. jejuni*	6175	42	CAT	mCCDA
CH-0321	CA21CH-0321-6-2	*C. jejuni*	6175	42	CAT	mCCDA
CH-0321	CA21CH-0321-6-3	*C. jejuni*	6175	42	CAT	mCCDA
CH-0321	CA21CH-0321-6-4	*C. jejuni*	5136	42	CAT	mCCDA
CH-0321	CA21CH-0321-6-5	*C. jejuni*	6175	42	CAT	u-mCCDA
CH-0321	CA21CH-0321-6-6	*C. jejuni*	5136	42	CAT	u-mCCDA
CH-0321	CA21CH-0321-6-7	*C. jejuni*	5136	42	CAT	u-mCCDA
CH-0321	CA21CH-0321-6-8	*C. jejuni*	6175	42	CAT	u-mCCDA
CH-0323	CA21CH-0323-6-1	*C. jejuni*	cj unknown7	42	CAT	mCCDA
CH-0323	CA21CH-0323-6-2	*C. jejuni*	cj unknown7	42	CAT	mCCDA
CH-0323	CA21CH-0323-6-3	*C. jejuni*	cj unknown7	42	CAT	mCCDA
CH-0323	CA21CH-0323-6-4	*C. jejuni*	cj unknown7	42	CAT	u-mCCDA
CH-0323	CA21CH-0323-6-5-R	*C. jejuni*	cj unknown7	42	CAT	u-mCCDA
CH-0323	CA21CH-0323-6-6	*C. jejuni*	cj unknown7	42	CAT	u-mCCDA
CH-0323	CA21CH-0323-6-7	*C. jejuni*	cj unknown7	42	CAT	u-mCCDA
CH-0325	CA21CH-0325-2-1	*C. jejuni*	21	37	Bolton	mCCDA
CH-0325	CA21CH-0325-3-1	*C. coli*	cc unknown4	37	CAT	mCCDA
CH-0325	CA21CH-0325-3-2	*C. jejuni*	21	37	CAT	mCCDA
CH-0325	CA21CH-0325-3-3	*C. jejuni*	21	37	CAT	mCCDA
CH-0325	CA21CH-0325-3-4	*C. jejuni*	21	37	CAT	mCCDA
CH-0325	CA21CH-0325-3-5-R	*C. jejuni*	21	37	CAT	u-mCCDA
CH-0325	CA21CH-0325-3-6	*C. jejuni*	21	37	CAT	u-mCCDA
CH-0325	CA21CH-0325-3-7	*C. jejuni*	21	37	CAT	u-mCCDA
CH-0325	CA21CH-0325-3-8	*C. jejuni*	21	37	CAT	u-mCCDA

CH-0325	CA21CH-0325-6-1	*C. coli*	1096	42	CAT	mCCDA
CH-0325	CA21CH-0325-6-2	*C. coli*	1096	42	CAT	u-mCCDA
CH-0325	CA21CH-0325-6-3	*C. jejuni*	21	42	CAT	u-mCCDA
CH-0325	CA21CH-0325-6-4	*C. jejuni*	21	42	CAT	u-mCCDA
CH-0325	CA21CH-0325-6-5	*C. jejuni*	21	42	CAT	u-mCCDA
CH-0325	CA21CH-0325-6-6	*C. jejuni*	21	42	CAT	u-mCCDA
CH-0325	CA21CH-0325-6-7	*C. coli*	1096	42	CAT	u-mCCDA
CH-0326	CA21CH-0326-3-1-R	*C. coli*	1595	37	CAT	mCCDA
CH-0326	CA21CH-0326-3-2	*C. coli*	1595	37	CAT	mCCDA
CH-0326	CA21CH-0326-3-3	*C. coli*	1595	37	CAT	mCCDA
CH-0326	CA21CH-0326-3-4	*C. coli*	1595	37	CAT	mCCDA
CH-0326	CA21CH-0326-3-5	*C. coli*	1595	37	CAT	mCCDA
CH-0326	CA21CH-0326-3-6	*C. coli*	1595	37	CAT	mCCDA
CH-0326	CA21CH-0326-3-7	*C. coli*	1595	37	CAT	mCCDA
CH-0326	CA21CH-0326-3-8	*C. coli*	1595	37	CAT	mCCDA
CH-0326	CA21CH-0326-5-1-R	*C. coli*	1595	42	Bolton	mCCDA
CH-0326	CA21CH-0326-5-2	*C. coli*	1595	42	Bolton	mCCDA
CH-0326	CA21CH-0326-5-3	*C. coli*	1595	42	Bolton	u-mCCDA
CH-0326	CA21CH-0326-5-4	*C. coli*	1595	42	Bolton	u-mCCDA
CH-0326	CA21CH-0326-5-5	*C. coli*	1595	42	Bolton	u-mCCDA
CH-0326	CA21CH-0326-5-6	*C. coli*	1595	42	Bolton	u-mCCDA
CH-0326	CA21CH-0326-6-1	*C. coli*	1595	42	CAT	mCCDA
CH-0327	CA21CH-0327-5-1	*C. coli*	1096	42	Bolton	mCCDA
CH-0327	CA21CH-0327-5-2	*C. coli*	1096	42	Bolton	mCCDA
CH-0327	CA21CH-0327-5-3	*C. coli*	1096	42	Bolton	mCCDA
CH-0327	CA21CH-0327-5-4	*C. coli*	1096	42	Bolton	mCCDA
CH-0327	CA21CH-0327-5-5	*C. coli*	1096	42	Bolton	u-mCCDA
CH-0327	CA21CH-0327-5-6	*C. coli*	1096	42	Bolton	u-mCCDA
CH-0327	CA21CH-0327-5-7	*C. coli*	1096	42	Bolton	u-mCCDA
CH-0327	CA21CH-0327-5-8	*C. coli*	1096	42	Bolton	u-mCCDA
CH-0327	CA21CH-0327-6-1	*C. jejuni*	2282	42	CAT	mCCDA
CH-0327	CA21CH-0327-6-2	*C. jejuni*	2282	42	CAT	mCCDA
CH-0327	CA21CH-0327-6-3	*C. jejuni*	2282	42	CAT	mCCDA
CH-0327	CA21CH-0327-6-4	*C. jejuni*	2282	42	CAT	u-mCCDA
CH-0327	CA21CH-0327-6-5	*C. jejuni*	2282	42	CAT	u-mCCDA
CH-0327	CA21CH-0327-6-6	*C. jejuni*	2282	42	CAT	u-mCCDA
CH-0327	CA21CH-0327-6-7	*C. jejuni*	2282	42	CAT	u-mCCDA
CH-0328	CA21CH-0328-3-1	*C. jejuni*	cj unknown4	37	CAT	mCCDA
CH-0328	CA21CH-0328-3-2	*C. jejuni*	cj unknown4	37	CAT	mCCDA
CH-0328	CA21CH-0328-3-3	*C. jejuni*	cj unknown4	37	CAT	mCCDA
CH-0328	CA21CH-0328-3-4	*C. jejuni*	9401	37	CAT	mCCDA
CH-0328	CA21CH-0328-3-5	*C. jejuni*	cj unknown4	37	CAT	mCCDA
CH-0328	CA21CH-0328-3-6	*C. jejuni*	cj unknown4	37	CAT	mCCDA
CH-0328	CA21CH-0328-3-7	*C. jejuni*	cj unknown4	37	CAT	mCCDA
CH-0328	CA21CH-0328-3-8	*C. jejuni*	814	37	CAT	mCCDA
CH-0328	CA21CH-0328-5-1	*C. jejuni*	814	42	Bolton	mCCDA
CH-0328	CA21CH-0328-5-2	*C. jejuni*	814	42	Bolton	mCCDA
CH-0328	CA21CH-0328-5-3	*C. jejuni*	814	42	Bolton	mCCDA
CH-0328	CA21CH-0328-5-4	*C. jejuni*	814	42	Bolton	mCCDA

CH-0328	CA21CH-0328-5-5	*C. jejuni*	814	42	Bolton	u-mCCDA
CH-0328	CA21CH-0328-5-6	*C. jejuni*	814	42	Bolton	u-mCCDA
CH-0328	CA21CH-0328-5-7	*C. jejuni*	814	42	Bolton	u-mCCDA
CH-0328	CA21CH-0328-5-8	*C. jejuni*	814	42	Bolton	u-mCCDA
CH-0328	CA21CH-0328-6-1	*C. jejuni*	814	42	CAT	mCCDA
CH-0329	CA21CH-0329-2-1	*C. coli*	829	37	Bolton	mCCDA
CH-0329	CA21CH-0329-2-2	*C. coli*	829	37	Bolton	mCCDA
CH-0329	CA21CH-0329-2-3	*C. coli*	829	37	Bolton	mCCDA
CH-0329	CA21CH-0329-2-4	*C. coli*	829	37	Bolton	mCCDA
CH-0329	CA21CH-0329-2-5	*C. coli*	829	37	Bolton	u-mCCDA
CH-0329	CA21CH-0329-2-6	*C. coli*	829	37	Bolton	u-mCCDA
CH-0329	CA21CH-0329-2-7	*C. coli*	829	37	Bolton	u-mCCDA
CH-0329	CA21CH-0329-2-8	*C. coli*	829	37	Bolton	u-mCCDA
CH-0329	CA21CH-0329-3-1	*C. coli*	829	37	CAT	mCCDA
CH-0329	CA21CH-0329-3-2	*C. coli*	829	37	CAT	mCCDA
CH-0329	CA21CH-0329-3-3	*C. coli*	829	37	CAT	mCCDA
CH-0329	CA21CH-0329-3-4	*C. coli*	828	37	CAT	mCCDA
CH-0329	CA21CH-0329-3-5	*C. coli*	829	37	CAT	u-mCCDA
CH-0329	CA21CH-0329-3-6	*C. coli*	829	37	CAT	u-mCCDA
CH-0329	CA21CH-0329-3-7	*C. coli*	829	37	CAT	u-mCCDA
CH-0329	CA21CH-0329-3-8	*C. coli*	829	37	CAT	u-mCCDA
CH-0329	CA21CH-0329-5-1	*C. coli*	829	42	Bolton	mCCDA
CH-0329	CA21CH-0329-5-2	*C. coli*	829	42	Bolton	mCCDA
CH-0329	CA21CH-0329-5-3	*C. coli*	829	42	Bolton	mCCDA
CH-0329	CA21CH-0329-5-4	*C. coli*	829	42	Bolton	mCCDA
CH-0329	CA21CH-0329-5-5	*C. coli*	829	42	Bolton	u-mCCDA
CH-0329	CA21CH-0329-5-6	*C. coli*	829	42	Bolton	u-mCCDA
CH-0329	CA21CH-0329-5-7	*C. coli*	829	42	Bolton	u-mCCDA
CH-0329	CA21CH-0329-5-8	*C. coli*	829	42	Bolton	u-mCCDA
CH-0329	CA21CH-0329-6-1	*C. coli*	829	42	CAT	mCCDA
CH-0329	CA21CH-0329-6-2	*C. coli*	829	42	CAT	mCCDA
CH-0329	CA21CH-0329-6-3	*C. coli*	829	42	CAT	mCCDA
CH-0329	CA21CH-0329-6-4	*C. coli*	829	42	CAT	mCCDA
CH-0329	CA21CH-0329-6-5	*C. coli*	829	42	CAT	u-mCCDA
CH-0329	CA21CH-0329-6-6	*C. coli*	829	42	CAT	u-mCCDA
CH-0329	CA21CH-0329-6-7	*C. coli*	829	42	CAT	u-mCCDA
CH-0329	CA21CH-0329-6-8	*C. coli*	829	42	CAT	u-mCCDA
CH-0330	CA21CH-0330-2-1	*C. coli*	1191	37	Bolton	mCCDA
CH-0330	CA21CH-0330-2-2	*C. coli*	1191	37	Bolton	mCCDA
CH-0330	CA21CH-0330-2-3	*C. coli*	1191	37	Bolton	mCCDA
CH-0330	CA21CH-0330-2-4	*C. coli*	1191	37	Bolton	mCCDA
CH-0330	CA21CH-0330-2-5	*C. coli*	1191	37	Bolton	u-mCCDA
CH-0330	CA21CH-0330-2-6	*C. coli*	1191	37	Bolton	u-mCCDA
CH-0330	CA21CH-0330-2-7	*C. coli*	1191	37	Bolton	u-mCCDA
CH-0330	CA21CH-0330-2-8	*C. coli*	1191	37	Bolton	u-mCCDA
CH-0330	CA21CH-0330-5-1	*C. coli*	1191	42	Bolton	mCCDA
CH-0330	CA21CH-0330-5-2	*C. coli*	1191	42	Bolton	mCCDA
CH-0330	CA21CH-0330-5-3	*C. coli*	1191	42	Bolton	mCCDA
CH-0330	CA21CH-0330-5-4	*C. coli*	1191	42	Bolton	mCCDA

CH-0330	CA21CH-0330-5-5	*C. coli*	1191	42	Bolton	u-mCCDA
CH-0330	CA21CH-0330-5-6	*C. coli*	1191	42	Bolton	u-mCCDA
CH-0330	CA21CH-0330-5-7	*C. coli*	1191	42	Bolton	u-mCCDA
CH-0330	CA21CH-0330-5-8	*C. coli*	1191	42	Bolton	u-mCCDA
CH-0330	CA21CH-0330-6-1	*C. jejuni*	441	42	CAT	mCCDA
CH-0330	CA21CH-0330-6-2	*C. coli*	1191	42	CAT	mCCDA
CH-0330	CA21CH-0330-6-3	*C. jejuni*	441	42	CAT	mCCDA
CH-0330	CA21CH-0330-6-4	*C. jejuni*	441	42	CAT	mCCDA
CH-0330	CA21CH-0330-6-5	*C. coli*	830	42	CAT	u-mCCDA
CH-0330	CA21CH-0330-6-6	*C. coli*	830	42	CAT	u-mCCDA
CH-0330	CA21CH-0330-6-7	*C. coli*	830	42	CAT	u-mCCDA
CH-0330	CA21CH-0330-6-8	*C. jejuni*	441	42	CAT	u-mCCDA
CH-0331	CA21CH-0331-2-1	*C. jejuni*	5136	37	Bolton	mCCDA
CH-0331	CA21CH-0331-2-2	*C. jejuni*	5136	37	Bolton	mCCDA
CH-0331	CA21CH-0331-2-3	*C. jejuni*	5136	37	Bolton	mCCDA
CH-0331	CA21CH-0331-2-4	*C. jejuni*	5136	37	Bolton	mCCDA
CH-0331	CA21CH-0331-2-5	*C. jejuni*	5136	37	Bolton	u-mCCDA
CH-0331	CA21CH-0331-2-6	*C. jejuni*	5136	37	Bolton	u-mCCDA
CH-0331	CA21CH-0331-2-7	*C. jejuni*	5136	37	Bolton	u-mCCDA
CH-0331	CA21CH-0331-2-8	*C. jejuni*	5136	37	Bolton	u-mCCDA
CH-0331	CA21CH-0331-5-1	*C. jejuni*	6175	42	Bolton	mCCDA
CH-0331	CA21CH-0331-5-2	*C. jejuni*	6175	42	Bolton	mCCDA
CH-0331	CA21CH-0331-5-3	*C. jejuni*	6175	42	Bolton	mCCDA
CH-0331	CA21CH-0331-5-4	*C. jejuni*	6175	42	Bolton	mCCDA
CH-0331	CA21CH-0331-5-5	*C. jejuni*	6175	42	Bolton	u-mCCDA
CH-0331	CA21CH-0331-5-6	*C. jejuni*	6175	42	Bolton	u-mCCDA
CH-0331	CA21CH-0331-5-7	*C. jejuni*	6175	42	Bolton	u-mCCDA
CH-0331	CA21CH-0331-5-8	*C. jejuni*	6175	42	Bolton	u-mCCDA
CH-0331	CA21CH-0331-6-1	*C. jejuni*	2282	42	CAT	mCCDA
CH-0331	CA21CH-0331-6-2	*C. jejuni*	2282	42	CAT	mCCDA
CH-0331	CA21CH-0331-6-3	*C. jejuni*	2282	42	CAT	mCCDA
CH-0331	CA21CH-0331-6-4	*C. jejuni*	2282	42	CAT	mCCDA
CH-0331	CA21CH-0331-6-5	*C. jejuni*	5136	42	CAT	u-mCCDA
CH-0331	CA21CH-0331-6-6	*C. jejuni*	2282	42	CAT	u-mCCDA
CH-0331	CA21CH-0331-6-7	*C. jejuni*	2282	42	CAT	u-mCCDA
CH-0331	CA21CH-0331-6-8	*C. jejuni*	2282	42	CAT	u-mCCDA
CH-0332	CA21CH-0332-3-1	*C. jejuni*	51	37	CAT	mCCDA
CH-0332	CA21CH-0332-3-2	*C. jejuni*	51	37	CAT	mCCDA
CH-0332	CA21CH-0332-3-3	*C. jejuni*	51	37	CAT	mCCDA
CH-0332	CA21CH-0332-3-4	*C. jejuni*	51	37	CAT	mCCDA
CH-0332	CA21CH-0332-3-5	*C. jejuni*	51	37	CAT	u-mCCDA
CH-0332	CA21CH-0332-3-6	*C. jejuni*	51	37	CAT	u-mCCDA
CH-0332	CA21CH-0332-3-7	*C. jejuni*	51	37	CAT	u-mCCDA
CH-0332	CA21CH-0332-3-8	*C. jejuni*	51	37	CAT	u-mCCDA
CH-0332	CA21CH-0332-6-1	*C. jejuni*	61	42	CAT	mCCDA
CH-0332	CA21CH-0332-6-2	*C. jejuni*	61	42	CAT	mCCDA
CH-0332	CA21CH-0332-6-3	*C. jejuni*	61	42	CAT	mCCDA
CH-0332	CA21CH-0332-6-4	*C. jejuni*	61	42	CAT	mCCDA
CH-0332	CA21CH-0332-6-5	*C. jejuni*	61	42	CAT	u-mCCDA

CH-0332	CA21CH-0332-6-6	*C. jejuni*	61	42	CAT	u-mCCDA
CH-0332	CA21CH-0332-6-7	*C. jejuni*	61	42	CAT	u-mCCDA
CH-0332	CA21CH-0332-6-8	*C. jejuni*	61	42	CAT	u-mCCDA
CH-0333	CA21CH-0333-1-1	*C. jejuni*	cj unknown1	37	None (Direct plating)	mCCDA
CH-0333	CA21CH-0333-1-2	*C. jejuni*	48	37	None (Direct plating)	mCCDA
CH-0333	CA21CH-0333-1-3	*C. jejuni*	cj unknown2	37	None (Direct plating)	mCCDA
CH-0333	CA21CH-0333-1-4	*C. jejuni*	53	37	None (Direct plating)	mCCDA
CH-0333	CA21CH-0333-1-5	*C. jejuni*	cj unknown1	37	None (Direct plating)	u-mCCDA
CH-0333	CA21CH-0333-1-6	*C. jejuni*	cj unknown2	37	None (Direct plating)	u-mCCDA
CH-0333	CA21CH-0333-2-1	*C. jejuni*	267	37	Bolton	mCCDA
CH-0333	CA21CH-0333-2-2	*C. jejuni*	267	37	Bolton	mCCDA
CH-0333	CA21CH-0333-2-3	*C. jejuni*	267	37	Bolton	mCCDA
CH-0333	CA21CH-0333-2-4	*C. jejuni*	267	37	Bolton	mCCDA
CH-0333	CA21CH-0333-2-5	*C. jejuni*	267	37	Bolton	u-mCCDA
CH-0333	CA21CH-0333-2-6	*C. jejuni*	267	37	Bolton	u-mCCDA
CH-0333	CA21CH-0333-2-7	*C. jejuni*	267	37	Bolton	u-mCCDA
CH-0333	CA21CH-0333-2-8	*C. jejuni*	267	37	Bolton	u-mCCDA
CH-0333	CA21CH-0333-4-1	*C. jejuni*	cj unknown3	42	None (Direct plating)	mCCDA
CH-0333	CA21CH-0333-4-2	*C. jejuni*	5136	42	None (Direct plating)	mCCDA
CH-0333	CA21CH-0333-5-1	*C. jejuni*	267	42	Bolton	mCCDA
CH-0333	CA21CH-0333-5-2	*C. jejuni*	267	42	Bolton	mCCDA
CH-0333	CA21CH-0333-5-3	*C. jejuni*	267	42	Bolton	mCCDA
CH-0333	CA21CH-0333-5-4	*C. jejuni*	267	42	Bolton	mCCDA
CH-0333	CA21CH-0333-5-5	*C. coli*	9012	42	Bolton	u-mCCDA
CH-0333	CA21CH-0333-5-6	*C. jejuni*	267	42	Bolton	u-mCCDA
CH-0333	CA21CH-0333-6-1	*C. coli*	9012	42	CAT	mCCDA
CH-0333	CA21CH-0333-6-2	*C. jejuni*	267	42	CAT	u-mCCDA
CH-0333	CA21CH-0333-6-3	*C. jejuni*	267	42	CAT	u-mCCDA
CH-0334	CA21CH-0334-2-1	*C. jejuni*	53	37	Bolton	mCCDA
CH-0334	CA21CH-0334-2-2	*C. jejuni*	53	37	Bolton	mCCDA
CH-0334	CA21CH-0334-2-3	*C. jejuni*	53	37	Bolton	mCCDA
CH-0334	CA21CH-0334-2-4	*C. jejuni*	53	37	Bolton	mCCDA
CH-0334	CA21CH-0334-2-5	*C. jejuni*	53	37	Bolton	u-mCCDA
CH-0334	CA21CH-0334-2-6	*C. jejuni*	53	37	Bolton	u-mCCDA
CH-0334	CA21CH-0334-2-7	*C. jejuni*	53	37	Bolton	u-mCCDA
CH-0334	CA21CH-0334-2-8	*C. jejuni*	53	37	Bolton	u-mCCDA
CH-0334	CA21CH-0334-4-1	*C. jejuni*	cj unknown2	42	None (Direct plating)	mCCDA
CH-0334	CA21CH-0334-4-2	*C. jejuni*	53	42	None (Direct plating)	mCCDA
CH-0334	CA21CH-0334-4-3	*C. jejuni*	257	42	None (Direct plating)	mCCDA
CH-0334	CA21CH-0334-5-1	*C. jejuni*	53	42	Bolton	mCCDA
CH-0334	CA21CH-0334-5-2	*C. jejuni*	230	42	Bolton	mCCDA
CH-0334	CA21CH-0334-5-3	*C. jejuni*	230	42	Bolton	mCCDA
CH-0334	CA21CH-0334-5-4	*C. jejuni*	53	42	Bolton	mCCDA
CH-0334	CA21CH-0334-5-5	*C. jejuni*	230	42	Bolton	u-mCCDA
CH-0334	CA21CH-0334-5-6	*C. jejuni*	230	42	Bolton	u-mCCDA
CH-0334	CA21CH-0334-5-7	*C. jejuni*	53	42	Bolton	u-mCCDA
CH-0334	CA21CH-0334-5-8	*C. jejuni*	53	42	Bolton	u-mCCDA
CH-0334	CA21CH-0334-6-1	*C. jejuni*	53	42	CAT	mCCDA
CH-0334	CA21CH-0334-6-2	*C. jejuni*	53	42	CAT	mCCDA

CH-0334	CA21CH-0334-6-3	*C. jejuni*	53	42	CAT	mCCDA
CH-0334	CA21CH-0334-6-4	*C. jejuni*	53	42	CAT	mCCDA
CH-0334	CA21CH-0334-6-5	*C. jejuni*	449	42	CAT	u-mCCDA
CH-0335	CA21CH-0335-1-1	*C. jejuni*	21	37	None (Direct plating)	mCCDA
CH-0335	CA21CH-0335-1-2	*C. jejuni*	6175	37	None (Direct plating)	mCCDA
CH-0335	CA21CH-0335-2-1	*C. coli*	cc unknown1	37	Bolton	mCCDA
CH-0335	CA21CH-0335-2-2	*C. coli*	cc unknown1	37	Bolton	mCCDA
CH-0335	CA21CH-0335-2-3	*C. coli*	cc unknown1	37	Bolton	mCCDA
CH-0335	CA21CH-0335-2-4	*C. jejuni*	227	37	Bolton	mCCDA
CH-0335	CA21CH-0335-2-5	*C. coli*	cc unknown1	37	Bolton	u-mCCDA
CH-0335	CA21CH-0335-2-6	*C. coli*	cc unknown1	37	Bolton	u-mCCDA
CH-0335	CA21CH-0335-2-7	*C. coli*	cc unknown1	37	Bolton	u-mCCDA
CH-0335	CA21CH-0335-2-8	*C. coli*	cc unknown1	37	Bolton	u-mCCDA
CH-0335	CA21CH-0335-3-1	*C. coli*	cc unknown1	37	CAT	mCCDA
CH-0335	CA21CH-0335-3-2	*C. jejuni*	227	37	CAT	mCCDA
CH-0335	CA21CH-0335-3-3	*C. coli*	cc unknown1	37	CAT	mCCDA
CH-0335	CA21CH-0335-3-4	*C. coli*	cc unknown1	37	CAT	mCCDA
CH-0335	CA21CH-0335-3-5	*C. coli*	cc unknown1	37	CAT	u-mCCDA
CH-0335	CA21CH-0335-3-6	*C. coli*	cc unknown1	37	CAT	u-mCCDA
CH-0335	CA21CH-0335-3-7	*C. coli*	cc unknown1	37	CAT	u-mCCDA
CH-0335	CA21CH-0335-3-8	*C. coli*	cc unknown1	37	CAT	u-mCCDA
CH-0335	CA21CH-0335-4-1	*C. jejuni*	2254	42	None (Direct plating)	mCCDA
CH-0335	CA21CH-0335-5-1	*C. coli*	cc unknown1	42	Bolton	mCCDA
CH-0335	CA21CH-0335-5-2	*C. coli*	cc unknown1	42	Bolton	mCCDA
CH-0335	CA21CH-0335-5-3	*C. coli*	cc unknown1	42	Bolton	mCCDA
CH-0335	CA21CH-0335-5-4	*C. coli*	cc unknown1	42	Bolton	mCCDA
CH-0335	CA21CH-0335-5-5	*C. coli*	cc unknown1	42	Bolton	u-mCCDA
CH-0335	CA21CH-0335-5-6	*C. coli*	cc unknown1	42	Bolton	u-mCCDA
CH-0335	CA21CH-0335-5-7	*C. coli*	cc unknown1	42	Bolton	u-mCCDA
CH-0335	CA21CH-0335-5-8	*C. coli*	cc unknown1	42	Bolton	u-mCCDA
CH-0335	CA21CH-0335-6-1	*C. coli*	cc unknown1	42	CAT	mCCDA
CH-0335	CA21CH-0335-6-2	*C. coli*	cc unknown1	42	CAT	mCCDA
CH-0335	CA21CH-0335-6-3	*C. coli*	cc unknown1	42	CAT	mCCDA
CH-0335	CA21CH-0335-6-4	*C. coli*	cc unknown1	42	CAT	mCCDA
CH-0335	CA21CH-0335-6-5	*C. coli*	cc unknown1	42	CAT	u-mCCDA
CH-0335	CA21CH-0335-6-6	*C. coli*	cc unknown1	42	CAT	u-mCCDA
CH-0335	CA21CH-0335-6-7	*C. coli*	cc unknown1	42	CAT	u-mCCDA
CH-0335	CA21CH-0335-6-8	*C. jejuni*	227	42	CAT	u-mCCDA
CH-0336	CA21CH-0336-2-1	*C. jejuni*	7743	37	Bolton	mCCDA
CH-0336	CA21CH-0336-2-2	*C. jejuni*	6175	37	Bolton	mCCDA
CH-0336	CA21CH-0336-2-3	*C. jejuni*	7743	37	Bolton	mCCDA
CH-0336	CA21CH-0336-2-4	*C. jejuni*	6175	37	Bolton	mCCDA
CH-0336	CA21CH-0336-2-5	*C. jejuni*	7743	37	Bolton	u-mCCDA
CH-0336	CA21CH-0336-2-6	*C. jejuni*	6175	37	Bolton	u-mCCDA
CH-0336	CA21CH-0336-2-7	*C. jejuni*	7743	37	Bolton	u-mCCDA
CH-0336	CA21CH-0336-2-8	*C. jejuni*	7743	37	Bolton	u-mCCDA
CH-0336	CA21CH-0336-3-1	*C. jejuni*	5136	37	CAT	mCCDA
CH-0336	CA21CH-0336-3-2	*C. jejuni*	6175	37	CAT	mCCDA
CH-0336	CA21CH-0336-3-3	*C. jejuni*	6175	37	CAT	mCCDA

CH-0336	CA21CH-0336-3-4	*C. jejuni*	6175	37	CAT	mCCDA
CH-0336	CA21CH-0336-3-5	*C. jejuni*	6175	37	CAT	u-mCCDA
CH-0336	CA21CH-0336-3-6	*C. jejuni*	6175	37	CAT	u-mCCDA
CH-0336	CA21CH-0336-3-7	*C. jejuni*	6175	37	CAT	u-mCCDA
CH-0336	CA21CH-0336-3-8	*C. jejuni*	6175	37	CAT	u-mCCDA
CH-0336	CA21CH-0336-5-1	*C. jejuni*	230	42	Bolton	mCCDA
CH-0336	CA21CH-0336-5-2	*C. jejuni*	230	42	Bolton	mCCDA
CH-0336	CA21CH-0336-5-3	*C. jejuni*	230	42	Bolton	mCCDA
CH-0336	CA21CH-0336-5-4	*C. jejuni*	230	42	Bolton	mCCDA
CH-0336	CA21CH-0336-5-5	*C. jejuni*	230	42	Bolton	u-mCCDA
CH-0336	CA21CH-0336-5-6	*C. jejuni*	230	42	Bolton	u-mCCDA
CH-0336	CA21CH-0336-5-7	*C. jejuni*	230	42	Bolton	u-mCCDA
CH-0336	CA21CH-0336-6-1	*C. jejuni*	6175	42	CAT	mCCDA
CH-0336	CA21CH-0336-6-2	*C. jejuni*	400	42	CAT	mCCDA
CH-0336	CA21CH-0336-6-3	*C. jejuni*	400	42	CAT	mCCDA
CH-0336	CA21CH-0336-6-4	*C. jejuni*	400	42	CAT	mCCDA
CH-0336	CA21CH-0336-6-5	*C. jejuni*	5136	42	CAT	u-mCCDA
CH-0336	CA21CH-0336-6-6	*C. jejuni*	400	42	CAT	u-mCCDA
CH-0336	CA21CH-0336-6-7	*C. jejuni*	400	42	CAT	u-mCCDA
CH-0336	CA21CH-0336-6-8	*C. jejuni*	5136	42	CAT	u-mCCDA
CH-0337	CA21CH-0337-2-1	*C. jejuni*	2066	37	Bolton	mCCDA
CH-0337	CA21CH-0337-2-2	*C. jejuni*	2211	37	Bolton	mCCDA
CH-0337	CA21CH-0337-2-3	*C. jejuni*	2066	37	Bolton	mCCDA
CH-0337	CA21CH-0337-2-4	*C. jejuni*	2066	37	Bolton	mCCDA
CH-0337	CA21CH-0337-2-5	*C. jejuni*	2066	37	Bolton	u-mCCDA
CH-0337	CA21CH-0337-2-6	*C. jejuni*	2066	37	Bolton	u-mCCDA
CH-0337	CA21CH-0337-2-7	*C. jejuni*	2211	37	Bolton	u-mCCDA
CH-0337	CA21CH-0337-2-8	*C. jejuni*	2211	37	Bolton	u-mCCDA
CH-0337	CA21CH-0337-4-1	*C. jejuni*	2066	42	None (Direct plating)	u-mCCDA
CH-0337	CA21CH-0337-5-1	*C. jejuni*	51	42	Bolton	mCCDA
CH-0337	CA21CH-0337-5-2	*C. jejuni*	2066	42	Bolton	mCCDA
CH-0337	CA21CH-0337-5-3	*C. jejuni*	400	42	Bolton	mCCDA
CH-0337	CA21CH-0337-5-4	*C. jejuni*	2066	42	Bolton	mCCDA
CH-0337	CA21CH-0337-5-5	*C. jejuni*	2066	42	Bolton	u-mCCDA
CH-0337	CA21CH-0337-5-6	*C. jejuni*	2066	42	Bolton	u-mCCDA
CH-0337	CA21CH-0337-5-7	*C. jejuni*	2066	42	Bolton	u-mCCDA
CH-0337	CA21CH-0337-5-8	*C. jejuni*	2066	42	Bolton	u-mCCDA
CH-0337	CA21CH-0337-6-1	*C. jejuni*	2066	42	CAT	mCCDA
CH-0337	CA21CH-0337-6-2	*C. jejuni*	2066	42	CAT	u-mCCDA
CH-0337	CA21CH-0337-6-3	*C. jejuni*	2066	42	CAT	u-mCCDA
CH-0337	CA21CH-0337-6-4	*C. jejuni*	2066	42	CAT	u-mCCDA
CH-0338	CA21CH-0338-2-1	*C. jejuni*	6876	37	Bolton	mCCDA
CH-0338	CA21CH-0338-2-2	*C. jejuni*	6876	37	Bolton	mCCDA
CH-0338	CA21CH-0338-2-3	*C. jejuni*	50	37	Bolton	mCCDA
CH-0338	CA21CH-0338-2-4	*C. coli*	cc unknown3	37	Bolton	mCCDA
CH-0338	CA21CH-0338-2-5	*C. jejuni*	6876	37	Bolton	u-mCCDA
CH-0338	CA21CH-0338-2-6	*C. jejuni*	6876	37	Bolton	u-mCCDA
CH-0338	CA21CH-0338-2-7	*C. jejuni*	6876	37	Bolton	u-mCCDA
CH-0338	CA21CH-0338-2-8	*C. jejuni*	6876	37	Bolton	u-mCCDA

CH-0338	CA21CH-0338-3-1	*C. coli*	828	37	CAT	mCCDA
CH-0338	CA21CH-0338-3-2	*C. coli*	cc unknown3	37	CAT	mCCDA
CH-0338	CA21CH-0338-3-3	*C. coli*	828	37	CAT	mCCDA
CH-0338	CA21CH-0338-3-4	*C. jejuni*	6175	37	CAT	mCCDA
CH-0338	CA21CH-0338-3-5	*C. coli*	cc unknown3	37	CAT	u-mCCDA
CH-0338	CA21CH-0338-3-6	*C. jejuni*	6175	37	CAT	u-mCCDA
CH-0338	CA21CH-0338-3-7	*C. coli*	828	37	CAT	u-mCCDA
CH-0338	CA21CH-0338-3-8	*C. coli*	828	37	CAT	u-mCCDA
CH-0338	CA21CH-0338-5-1	*C. coli*	1541	42	Bolton	mCCDA
CH-0338	CA21CH-0338-5-2	*C. coli*	1541	42	Bolton	mCCDA
CH-0338	CA21CH-0338-5-3	*C. coli*	1541	42	Bolton	mCCDA
CH-0338	CA21CH-0338-5-4	*C. coli*	cc unknown3	42	Bolton	mCCDA
CH-0338	CA21CH-0338-5-5	*C. coli*	1541	42	Bolton	u-mCCDA
CH-0338	CA21CH-0338-5-6	*C. coli*	1541	42	Bolton	u-mCCDA
CH-0338	CA21CH-0338-5-7	*C. coli*	cc unknown3	42	Bolton	u-mCCDA
CH-0338	CA21CH-0338-5-8	*C. coli*	1541	42	Bolton	u-mCCDA
CH-0338	CA21CH-0338-6-1	*C. coli*	cc unknown3	42	CAT	mCCDA
CH-0338	CA21CH-0338-6-2	*C. coli*	cc unknown3	42	CAT	mCCDA
CH-0338	CA21CH-0338-6-3	*C. coli*	cc unknown3	42	CAT	mCCDA
CH-0338	CA21CH-0338-6-4	*C. coli*	cc unknown3	42	CAT	mCCDA
CH-0338	CA21CH-0338-6-5	*C. coli*	1541	42	CAT	u-mCCDA
CH-0338	CA21CH-0338-6-6	*C. coli*	cc unknown3	42	CAT	u-mCCDA
CH-0338	CA21CH-0338-6-7	*C. coli*	cc unknown3	42	CAT	u-mCCDA
CH-0338	CA21CH-0338-6-8	*C. coli*	cc unknown3	42	CAT	u-mCCDA
CH-0339	CA21CH-0339-2-1	*C. jejuni*	cj unknown5	37	Bolton	mCCDA
CH-0339	CA21CH-0339-2-2	*C. jejuni*	cj unknown5	37	Bolton	mCCDA
CH-0339	CA21CH-0339-2-3	*C. jejuni*	cj unknown5	37	Bolton	mCCDA
CH-0339	CA21CH-0339-2-4	*C. jejuni*	cj unknown5	37	Bolton	mCCDA
CH-0339	CA21CH-0339-2-5	*C. jejuni*	cj unknown5	37	Bolton	u-mCCDA
CH-0339	CA21CH-0339-2-6	*C. jejuni*	cj unknown5	37	Bolton	u-mCCDA
CH-0339	CA21CH-0339-2-7	*C. jejuni*	cj unknown5	37	Bolton	u-mCCDA
CH-0339	CA21CH-0339-2-8	*C. jejuni*	cj unknown5	37	Bolton	u-mCCDA
CH-0339	CA21CH-0339-3-1	*C. jejuni*	51	37	CAT	mCCDA
CH-0339	CA21CH-0339-3-2	*C. jejuni*	51	37	CAT	mCCDA
CH-0339	CA21CH-0339-3-3	*C. jejuni*	51	37	CAT	mCCDA
CH-0339	CA21CH-0339-3-4	*C. jejuni*	51	37	CAT	mCCDA
CH-0339	CA21CH-0339-3-5	*C. jejuni*	51	37	CAT	u-mCCDA
CH-0339	CA21CH-0339-3-6	*C. jejuni*	51	37	CAT	u-mCCDA
CH-0339	CA21CH-0339-3-7	*C. jejuni*	51	37	CAT	u-mCCDA
CH-0339	CA21CH-0339-3-8	*C. jejuni*	51	37	CAT	u-mCCDA
CH-0339	CA21CH-0339-5-1	*C. jejuni*	51	42	Bolton	mCCDA
CH-0339	CA21CH-0339-5-2	*C. jejuni*	51	42	Bolton	mCCDA
CH-0339	CA21CH-0339-5-3	*C. jejuni*	51	42	Bolton	mCCDA
CH-0339	CA21CH-0339-5-4	*C. jejuni*	51	42	Bolton	mCCDA
CH-0339	CA21CH-0339-5-5	*C. jejuni*	51	42	Bolton	u-mCCDA
CH-0339	CA21CH-0339-5-6	*C. jejuni*	51	42	Bolton	u-mCCDA
CH-0339	CA21CH-0339-5-7	*C. jejuni*	51	42	Bolton	u-mCCDA
CH-0339	CA21CH-0339-5-8	*C. jejuni*	51	42	Bolton	u-mCCDA
CH-0339	CA21CH-0339-6-1	*C. jejuni*	2258	42	CAT	mCCDA

CH-0339	CA21CH-0339-6-2	*C. jejuni*	2258	42	CAT	mCCDA
CH-0339	CA21CH-0339-6-3	*C. coli*	6795	42	CAT	mCCDA
CH-0339	CA21CH-0339-6-4	*C. coli*	6795	42	CAT	mCCDA
CH-0339	CA21CH-0339-6-5	*C. coli*	6795	42	CAT	u-mCCDA
CH-0339	CA21CH-0339-6-6	*C. coli*	6795	42	CAT	u-mCCDA
CH-0339	CA21CH-0339-6-7	*C. jejuni*	2258	42	CAT	u-mCCDA
CH-0340	CA21CH-0340-1-1	*C. jejuni*	257	37	None (Direct plating)	u-mCCDA
CH-0340	CA21CH-0340-2-1	*C. jejuni*	441	37	Bolton	mCCDA
CH-0340	CA21CH-0340-2-2	*C. jejuni*	441	37	Bolton	mCCDA
CH-0340	CA21CH-0340-2-3	*C. jejuni*	441	37	Bolton	mCCDA
CH-0340	CA21CH-0340-2-4	*C. jejuni*	441	37	Bolton	mCCDA
CH-0340	CA21CH-0340-2-5	*C. jejuni*	441	37	Bolton	u-mCCDA
CH-0340	CA21CH-0340-2-6	*C. jejuni*	441	37	Bolton	u-mCCDA
CH-0340	CA21CH-0340-2-7	*C. jejuni*	257	37	Bolton	u-mCCDA
CH-0340	CA21CH-0340-2-8	*C. jejuni*	441	37	Bolton	u-mCCDA
CH-0340	CA21CH-0340-3-1	*C. jejuni*	447	37	CAT	mCCDA
CH-0340	CA21CH-0340-3-2	*C. jejuni*	6175	37	CAT	mCCDA
CH-0340	CA21CH-0340-3-3	*C. jejuni*	6175	37	CAT	mCCDA
CH-0340	CA21CH-0340-3-4	*C. jejuni*	447	37	CAT	mCCDA
CH-0340	CA21CH-0340-5-1	*C. jejuni*	441	42	Bolton	mCCDA
CH-0340	CA21CH-0340-5-2	*C. jejuni*	441	42	Bolton	mCCDA
CH-0340	CA21CH-0340-5-3	*C. jejuni*	441	42	Bolton	mCCDA
CH-0340	CA21CH-0340-5-4	*C. jejuni*	441	42	Bolton	mCCDA
CH-0340	CA21CH-0340-5-5	*C. coli*	827	42	Bolton	u-mCCDA
CH-0340	CA21CH-0340-5-6	*C. coli*	827	42	Bolton	u-mCCDA
CH-0340	CA21CH-0340-5-7	*C. coli*	cc unknown3	42	Bolton	u-mCCDA
CH-0340	CA21CH-0340-5-8	*C. jejuni*	441	42	Bolton	u-mCCDA
CH-0340	CA21CH-0340-6-1	*C. coli*	cc unknown3	42	CAT	mCCDA
CH-0340	CA21CH-0340-6-2	*C. coli*	cc unknown3	42	CAT	mCCDA
CH-0340	CA21CH-0340-6-3	*C. jejuni*	441	42	CAT	mCCDA
CH-0340	CA21CH-0340-6-4	*C. coli*	cc unknown3	42	CAT	u-mCCDA
CH-0340	CA21CH-0340-6-5	*C. coli*	cc unknown3	42	CAT	u-mCCDA
CH-0340	CA21CH-0340-6-6	*C. coli*	cc unknown3	42	CAT	u-mCCDA
CH-0340	CA21CH-0340-6-7	*C. coli*	cc unknown3	42	CAT	u-mCCDA
CH-0341	CA21CH-0341-2-1	*C. coli*	cc unknown3	37	Bolton	mCCDA
CH-0341	CA21CH-0341-2-2	*C. coli*	cc unknown3	37	Bolton	mCCDA
CH-0341	CA21CH-0341-2-3	*C. coli*	1595	37	Bolton	mCCDA
CH-0341	CA21CH-0341-2-4	*C. coli*	1595	37	Bolton	mCCDA
CH-0341	CA21CH-0341-2-5	*C. jejuni*	447	37	Bolton	u-mCCDA
CH-0341	CA21CH-0341-2-6	*C. jejuni*	6175	37	Bolton	u-mCCDA
CH-0341	CA21CH-0341-2-7	*C. coli*	cc unknown3	37	Bolton	u-mCCDA
CH-0341	CA21CH-0341-2-8	*C. jejuni*	447	37	Bolton	u-mCCDA
CH-0341	CA21CH-0341-3-1	*C. jejuni*	21	37	CAT	mCCDA
CH-0341	CA21CH-0341-3-2	*C. jejuni*	21	37	CAT	mCCDA
CH-0341	CA21CH-0341-3-3	*C. jejuni*	21	37	CAT	mCCDA
CH-0341	CA21CH-0341-3-4	*C. jejuni*	21	37	CAT	mCCDA
CH-0341	CA21CH-0341-3-5	*C. jejuni*	21	37	CAT	u-mCCDA
CH-0341	CA21CH-0341-3-6	*C. jejuni*	21	37	CAT	u-mCCDA
CH-0341	CA21CH-0341-3-7	*C. jejuni*	21	37	CAT	u-mCCDA

CH-0341	CA21CH-0341-3-8	*C. jejuni*	21	37	CAT	u-mCCDA
CH-0341	CA21CH-0341-5-1	*C. coli*	827	42	Bolton	mCCDA
CH-0341	CA21CH-0341-5-2	*C. coli*	827	42	Bolton	mCCDA
CH-0341	CA21CH-0341-5-3	*C. coli*	827	42	Bolton	mCCDA
CH-0341	CA21CH-0341-5-4	*C. coli*	827	42	Bolton	mCCDA
CH-0341	CA21CH-0341-5-5	*C. coli*	827	42	Bolton	u-mCCDA
CH-0341	CA21CH-0341-5-6	*C. coli*	827	42	Bolton	u-mCCDA
CH-0341	CA21CH-0341-5-7	*C. coli*	827	42	Bolton	u-mCCDA
CH-0341	CA21CH-0341-5-8	*C. coli*	827	42	Bolton	u-mCCDA
CH-0341	CA21CH-0341-6-1	*C. coli*	827	42	CAT	mCCDA
CH-0341	CA21CH-0341-6-2	*C. coli*	827	42	CAT	mCCDA
CH-0341	CA21CH-0341-6-3	*C. coli*	827	42	CAT	mCCDA
CH-0341	CA21CH-0341-6-4	*C. coli*	827	42	CAT	mCCDA
CH-0341	CA21CH-0341-6-5	*C. coli*	827	42	CAT	u-mCCDA
CH-0341	CA21CH-0341-6-6	*C. coli*	827	42	CAT	u-mCCDA
CH-0341	CA21CH-0341-6-7	*C. jejuni*	cj unknown8	42	CAT	u-mCCDA
CH-0341	CA21CH-0341-6-8	*C. coli*	827	42	CAT	u-mCCDA
CH-0347	CA21CH-0347-3-1	*C. jejuni*	262	37	CAT	mCCDA
CH-0347	CA21CH-0347-3-2	*C. jejuni*	262	37	CAT	mCCDA
CH-0347	CA21CH-0347-3-3	*C. jejuni*	262	37	CAT	mCCDA
CH-0347	CA21CH-0347-5-1	*C. jejuni*	262	42	Bolton	mCCDA
CH-0347	CA21CH-0347-5-2	*C. jejuni*	262	42	Bolton	mCCDA
CH-0347	CA21CH-0347-5-3	*C. jejuni*	262	42	Bolton	mCCDA
CH-0347	CA21CH-0347-5-4	*C. jejuni*	262	42	Bolton	u-mCCDA
CH-0347	CA21CH-0347-5-5	*C. jejuni*	262	42	Bolton	u-mCCDA
CH-0347	CA21CH-0347-5-6	*C. jejuni*	262	42	Bolton	u-mCCDA
CH-0347	CA21CH-0347-6-1	*C. jejuni*	262	42	CAT	mCCDA
CH-0347	CA21CH-0347-6-2	*C. jejuni*	262	42	CAT	mCCDA
CH-0347	CA21CH-0347-6-3	*C. jejuni*	262	42	CAT	mCCDA
CH-0347	CA21CH-0347-6-4	*C. jejuni*	262	42	CAT	u-mCCDA
CH-0347	CA21CH-0347-6-5	*C. jejuni*	262	42	CAT	u-mCCDA
CH-0347	CA21CH-0347-6-6	*C. jejuni*	262	42	CAT	u-mCCDA
CH-0348	CA21CH-0348-6-1	*C. coli*	827	42	CAT	mCCDA
CH-0348	CA21CH-0348-6-2	*C. coli*	827	42	CAT	mCCDA
CH-0348	CA21CH-0348-6-3	*C. coli*	827	42	CAT	mCCDA
CH-0348	CA21CH-0348-6-4	*C. coli*	827	42	CAT	u-mCCDA
CH-0348	CA21CH-0348-6-5	*C. coli*	827	42	CAT	u-mCCDA
CH-0348	CA21CH-0348-6-6	*C. coli*	827	42	CAT	u-mCCDA
CH-0349	CA21CH-0349-2-1	*C. coli*	cc unknown2	37	Bolton	mCCDA
CH-0349	CA21CH-0349-2-2	*C. coli*	cc unknown2	37	Bolton	mCCDA
CH-0349	CA21CH-0349-2-3	*C. coli*	cc unknown2	37	Bolton	mCCDA
CH-0349	CA21CH-0349-2-4	*C. coli*	cc unknown2	37	Bolton	u-mCCDA
CH-0349	CA21CH-0349-2-5	*C. coli*	cc unknown2	37	Bolton	u-mCCDA
CH-0349	CA21CH-0349-2-6	*C. coli*	cc unknown2	37	Bolton	u-mCCDA
CH-0349	CA21CH-0349-5-1	*C. jejuni*	814	42	Bolton	mCCDA
CH-0349	CA21CH-0349-5-2	*C. jejuni*	814	42	Bolton	mCCDA
CH-0349	CA21CH-0349-5-3	*C. jejuni*	814	42	Bolton	mCCDA
CH-0349	CA21CH-0349-5-4	*C. jejuni*	814	42	Bolton	u-mCCDA
CH-0349	CA21CH-0349-5-5	*C. jejuni*	814	42	Bolton	u-mCCDA

CH-0349	CA21CH-0349-5-6	*C. jejuni*	814	42	Bolton	u-mCCDA
CH-0349	CA21CH-0349-6-1	*C. coli*	cc unknown2	42	CAT	mCCDA
CH-0349	CA21CH-0349-6-2	*C. coli*	cc unknown2	42	CAT	mCCDA
CH-0349	CA21CH-0349-6-3	*C. coli*	cc unknown2	42	CAT	mCCDA
CH-0349	CA21CH-0349-6-4	*C. coli*	cc unknown2	42	CAT	u-mCCDA
CH-0349	CA21CH-0349-6-5	*C. coli*	cc unknown2	42	CAT	u-mCCDA
CH-0349	CA21CH-0349-6-6	*C. coli*	cc unknown2	42	CAT	u-mCCDA
CH-0350	CA21CH-0350-2-1	*C. jejuni*	19	37	Bolton	mCCDA
CH-0350	CA21CH-0350-2-2	*C. jejuni*	19	37	Bolton	mCCDA
CH-0350	CA21CH-0350-2-3	*C. jejuni*	48	37	Bolton	mCCDA
CH-0350	CA21CH-0350-2-4	*C. jejuni*	48	37	Bolton	u-mCCDA
CH-0350	CA21CH-0350-2-5	*C. jejuni*	48	37	Bolton	u-mCCDA
CH-0350	CA21CH-0350-2-6	*C. jejuni*	19	37	Bolton	u-mCCDA
CH-0350	CA21CH-0350-3-1	*C. jejuni*	2066	37	CAT	mCCDA
CH-0350	CA21CH-0350-3-2	*C. jejuni*	2066	37	CAT	mCCDA
CH-0350	CA21CH-0350-3-3	*C. jejuni*	50	37	CAT	mCCDA
CH-0350	CA21CH-0350-5-1	*C. jejuni*	19	42	Bolton	mCCDA
CH-0350	CA21CH-0350-5-2	*C. jejuni*	19	42	Bolton	mCCDA
CH-0350	CA21CH-0350-5-3	*C. jejuni*	19	42	Bolton	mCCDA
CH-0350	CA21CH-0350-5-4	*C. jejuni*	19	42	Bolton	u-mCCDA
CH-0350	CA21CH-0350-5-5	*C. jejuni*	19	42	Bolton	u-mCCDA
CH-0350	CA21CH-0350-5-6	*C. jejuni*	19	42	Bolton	u-mCCDA
CH-0350	CA21CH-0350-6-1	*C. jejuni*	50	42	CAT	mCCDA
CH-0350	CA21CH-0350-6-2	*C. jejuni*	50	42	CAT	mCCDA
CH-0350	CA21CH-0350-6-3	*C. jejuni*	50	42	CAT	mCCDA
CH-0350	CA21CH-0350-6-4	*C. jejuni*	8334	42	CAT	u-mCCDA
CH-0350	CA21CH-0350-6-5	*C. jejuni*	2066	42	CAT	u-mCCDA
CH-0350	CA21CH-0350-6-6	*C. jejuni*	2066	42	CAT	u-mCCDA
CH-0351	CA21CH-0351-1-1	*C. coli*	825	37	None (Direct plating)	mCCDA
CH-0351	CA21CH-0351-2-1	*C. jejuni*	cj unknown8	37	Bolton	mCCDA
CH-0351	CA21CH-0351-2-2	*C. coli*	829	37	Bolton	mCCDA
CH-0351	CA21CH-0351-2-3	*C. jejuni*	48	37	Bolton	mCCDA
CH-0351	CA21CH-0351-2-4	*C. jejuni*	cj unknown8	37	Bolton	u-mCCDA
CH-0351	CA21CH-0351-2-5	*C. jejuni*	48	37	Bolton	u-mCCDA
CH-0351	CA21CH-0351-2-6	*C. jejuni*	cj unknown8	37	Bolton	u-mCCDA
CH-0351	CA21CH-0351-3-1	*C. coli*	829	37	CAT	mCCDA
CH-0351	CA21CH-0351-5-1	*C. coli*	825	42	Bolton	mCCDA
CH-0351	CA21CH-0351-5-2	*C. coli*	825	42	Bolton	mCCDA
CH-0351	CA21CH-0351-5-3	*C. coli*	825	42	Bolton	mCCDA
CH-0351	CA21CH-0351-5-4	*C. coli*	825	42	Bolton	u-mCCDA
CH-0351	CA21CH-0351-5-5	*C. coli*	825	42	Bolton	u-mCCDA
CH-0351	CA21CH-0351-5-6	*C. coli*	825	42	Bolton	u-mCCDA
CH-0351	CA21CH-0351-6-1	*C. coli*	825	42	CAT	mCCDA
CH-0351	CA21CH-0351-6-2	*C. coli*	825	42	CAT	mCCDA
CH-0351	CA21CH-0351-6-3	*C. coli*	825	42	CAT	mCCDA
CH-0353	CA21CH-0353-2-1	*C. jejuni*	814	37	Bolton	mCCDA
CH-0353	CA21CH-0353-2-2	*C. jejuni*	814	37	Bolton	mCCDA
CH-0353	CA21CH-0353-2-3	*C. jejuni*	814	37	Bolton	mCCDA
CH-0353	CA21CH-0353-2-4	*C. jejuni*	814	37	Bolton	u-mCCDA

CH-0353	CA21CH-0353-2-5	*C. jejuni*	814	37	Bolton	u-mCCDA
CH-0353	CA21CH-0353-2-6	*C. jejuni*	814	37	Bolton	u-mCCDA
CH-0353	CA21CH-0353-3-1	*C. jejuni*	814	37	CAT	mCCDA
CH-0353	CA21CH-0353-3-2	*C. jejuni*	814	37	CAT	mCCDA
CH-0353	CA21CH-0353-3-3	*C. jejuni*	814	37	CAT	mCCDA
CH-0353	CA21CH-0353-3-4	*C. jejuni*	814	37	CAT	u-mCCDA
CH-0353	CA21CH-0353-3-5	*C. jejuni*	814	37	CAT	u-mCCDA
CH-0353	CA21CH-0353-3-6	*C. jejuni*	814	37	CAT	u-mCCDA
CH-0353	CA21CH-0353-5-1	*C. coli*	827	42	Bolton	mCCDA
CH-0353	CA21CH-0353-5-2	*C. coli*	827	42	Bolton	mCCDA
CH-0353	CA21CH-0353-5-3	*C. coli*	827	42	Bolton	mCCDA
CH-0353	CA21CH-0353-5-4	*C. coli*	827	42	Bolton	u-mCCDA
CH-0353	CA21CH-0353-5-5	*C. coli*	827	42	Bolton	u-mCCDA
CH-0353	CA21CH-0353-5-6	*C. coli*	827	42	Bolton	u-mCCDA
CH-0353	CA21CH-0353-6-1	*C. jejuni*	814	42	CAT	mCCDA
CH-0353	CA21CH-0353-6-2	*C. jejuni*	814	42	CAT	mCCDA
CH-0353	CA21CH-0353-6-3	*C. jejuni*	814	42	CAT	mCCDA
CH-0353	CA21CH-0353-6-4	*C. jejuni*	814	42	CAT	u-mCCDA
CH-0353	CA21CH-0353-6-5	*C. jejuni*	814	42	CAT	u-mCCDA
CH-0353	CA21CH-0353-6-6	*C. jejuni*	814	42	CAT	u-mCCDA
CH-0355	CA21CH-0355-3-1	*C. jejuni*	9401	37	CAT	mCCDA
CH-0355	CA21CH-0355-3-2	*C. jejuni*	9401	37	CAT	mCCDA
CH-0355	CA21CH-0355-3-3	*C. jejuni*	9401	37	CAT	mCCDA
CH-0355	CA21CH-0355-5-1	*C. jejuni*	9401	42	Bolton	mCCDA
CH-0355	CA21CH-0355-5-2	*C. jejuni*	9401	42	Bolton	mCCDA
CH-0355	CA21CH-0355-5-3	*C. jejuni*	9401	42	Bolton	mCCDA
CH-0355	CA21CH-0355-5-4	*C. jejuni*	9401	42	Bolton	u-mCCDA
CH-0355	CA21CH-0355-5-5	*C. jejuni*	9401	42	Bolton	u-mCCDA
CH-0355	CA21CH-0355-5-6	*C. jejuni*	9401	42	Bolton	u-mCCDA
CH-0356	CA21CH-0356-6-1	*C. jejuni*	305	42	CAT	mCCDA
CH-0356	CA21CH-0356-6-2	*C. jejuni*	305	42	CAT	mCCDA
CH-0356	CA21CH-0356-6-3	*C. jejuni*	305	42	CAT	mCCDA
CH-0356	CA21CH-0356-6-4	*C. jejuni*	305	42	CAT	u-mCCDA
CH-0356	CA21CH-0356-6-5	*C. jejuni*	305	42	CAT	u-mCCDA
CH-0356	CA21CH-0356-6-6	*C. jejuni*	305	42	CAT	u-mCCDA
CH-0357	CA21CH-0357-2-1	*C. jejuni*	cj unknown9	37	Bolton	mCCDA
CH-0357	CA21CH-0357-2-2	*C. jejuni*	cj unknown9	37	Bolton	mCCDA
CH-0357	CA21CH-0357-2-3	*C. jejuni*	cj unknown9	37	Bolton	mCCDA
CH-0357	CA21CH-0357-2-4	*C. jejuni*	cj unknown9	37	Bolton	u-mCCDA
CH-0357	CA21CH-0357-2-5	*C. jejuni*	cj unknown9	37	Bolton	u-mCCDA
CH-0357	CA21CH-0357-2-6	*C. jejuni*	cj unknown9	37	Bolton	u-mCCDA
CH-0357	CA21CH-0357-3-1	*C. jejuni*	918	37	CAT	mCCDA
CH-0357	CA21CH-0357-3-2	*C. jejuni*	918	37	CAT	mCCDA
CH-0357	CA21CH-0357-3-3	*C. jejuni*	918	37	CAT	mCCDA
CH-0357	CA21CH-0357-5-1	*C. jejuni*	2036	42	Bolton	mCCDA
CH-0357	CA21CH-0357-5-2	*C. jejuni*	2036	42	Bolton	mCCDA
CH-0357	CA21CH-0357-5-3	*C. jejuni*	2036	42	Bolton	mCCDA
CH-0357	CA21CH-0357-5-4	*C. jejuni*	2036	42	Bolton	u-mCCDA
CH-0357	CA21CH-0357-5-5	*C. jejuni*	2036	42	Bolton	u-mCCDA

CH-0357	CA21CH-0357-5-6	*C. jejuni*	2036	42	Bolton	u-mCCDA
CH-0357	CA21CH-0357-6-1	*C. jejuni*	918	42	CAT	mCCDA
CH-0357	CA21CH-0357-6-2	*C. jejuni*	918	42	CAT	mCCDA
CH-0357	CA21CH-0357-6-3	*C. jejuni*	918	42	CAT	mCCDA
CH-0357	CA21CH-0357-6-4	*C. jejuni*	918	42	CAT	u-mCCDA
CH-0357	CA21CH-0357-6-5	*C. jejuni*	918	42	CAT	u-mCCDA
CH-0357	CA21CH-0357-6-6	*C. jejuni*	918	42	CAT	u-mCCDA
CH-0358	CA21CH-0358-5-1	*C. jejuni*	6175	42	Bolton	mCCDA
CH-0358	CA21CH-0358-5-2	*C. jejuni*	6175	42	Bolton	mCCDA
CH-0358	CA21CH-0358-5-3	*C. jejuni*	6175	42	Bolton	mCCDA
CH-0358	CA21CH-0358-5-4	*C. jejuni*	6175	42	Bolton	u-mCCDA
CH-0358	CA21CH-0358-5-5	*C. jejuni*	6175	42	Bolton	u-mCCDA
CH-0358	CA21CH-0358-5-6	*C. jejuni*	6175	42	Bolton	u-mCCDA
CH-0359	CA21CH-0359-2-1	*C. coli*	962	37	Bolton	mCCDA
CH-0359	CA21CH-0359-2-2	*C. jejuni*	6175	37	Bolton	mCCDA
CH-0359	CA21CH-0359-2-3	*C. coli*	962	37	Bolton	mCCDA
CH-0359	CA21CH-0359-2-4	*C. jejuni*	6175	37	Bolton	u-mCCDA
CH-0359	CA21CH-0359-2-5	*C. coli*	962	37	Bolton	u-mCCDA
CH-0359	CA21CH-0359-2-6	*C. coli*	962	37	Bolton	u-mCCDA
CH-0359	CA21CH-0359-3-1	*C. coli*	962	37	CAT	mCCDA
CH-0359	CA21CH-0359-3-2	*C. jejuni*	6175	37	CAT	mCCDA
CH-0359	CA21CH-0359-3-3	*C. coli*	962	37	CAT	mCCDA
CH-0359	CA21CH-0359-3-4	*C. coli*	962	37	CAT	u-mCCDA
CH-0359	CA21CH-0359-3-5	*C. coli*	962	37	CAT	u-mCCDA
CH-0359	CA21CH-0359-3-6	*C. coli*	962	37	CAT	u-mCCDA
CH-0359	CA21CH-0359-5-1	*C. coli*	962	42	Bolton	mCCDA
CH-0359	CA21CH-0359-5-2	*C. coli*	962	42	Bolton	mCCDA
CH-0359	CA21CH-0359-5-3	*C. coli*	962	42	Bolton	mCCDA
CH-0359	CA21CH-0359-5-4	*C. coli*	962	42	Bolton	u-mCCDA
CH-0359	CA21CH-0359-5-5	*C. coli*	962	42	Bolton	u-mCCDA
CH-0359	CA21CH-0359-5-6	*C. coli*	962	42	Bolton	u-mCCDA
CH-0359	CA21CH-0359-6-1	*C. coli*	962	42	CAT	mCCDA
CH-0359	CA21CH-0359-6-2	*C. coli*	962	42	CAT	mCCDA
CH-0359	CA21CH-0359-6-3	*C. coli*	962	42	CAT	u-mCCDA
CH-0359	CA21CH-0359-6-4	*C. coli*	962	42	CAT	u-mCCDA
CH-0359	CA21CH-0359-6-5	*C. coli*	962	42	CAT	u-mCCDA
CH-0361	CA21CH-0361-2-1	*C. jejuni*	cj unknown10	37	Bolton	mCCDA
CH-0361	CA21CH-0361-2-2	*C. jejuni*	cj unknown10	37	Bolton	mCCDA
CH-0361	CA21CH-0361-2-3	*C. jejuni*	cj unknown10	37	Bolton	mCCDA
CH-0361	CA21CH-0361-2-4	*C. jejuni*	cj unknown10	37	Bolton	u-mCCDA
CH-0361	CA21CH-0361-2-5	*C. jejuni*	cj unknown10	37	Bolton	u-mCCDA
CH-0361	CA21CH-0361-2-6	*C. jejuni*	cj unknown10	37	Bolton	u-mCCDA
CH-0361	CA21CH-0361-3-1	*C. coli*	1541	37	CAT	mCCDA
CH-0361	CA21CH-0361-3-2	*C. coli*	1541	37	CAT	mCCDA
CH-0361	CA21CH-0361-3-3	*C. coli*	1541	37	CAT	mCCDA
CH-0361	CA21CH-0361-3-4	*C. coli*	1541	37	CAT	u-mCCDA
CH-0361	CA21CH-0361-3-5	*C. coli*	1541	37	CAT	u-mCCDA
CH-0361	CA21CH-0361-3-6	*C. coli*	1541	37	CAT	u-mCCDA
CH-0361	CA21CH-0361-5-1	*C. jejuni*	574	42	Bolton	mCCDA

CH-0361	CA21CH-0361-5-2	*C. jejuni*	574	42	Bolton	mCCDA
CH-0361	CA21CH-0361-5-3	*C. jejuni*	574	42	Bolton	mCCDA
CH-0361	CA21CH-0361-5-4	*C. jejuni*	574	42	Bolton	u-mCCDA
CH-0361	CA21CH-0361-5-5	*C. jejuni*	574	42	Bolton	u-mCCDA
CH-0361	CA21CH-0361-5-6	*C. jejuni*	574	42	Bolton	u-mCCDA
CH-0361	CA21CH-0361-6-1	*C. jejuni*	574	42	CAT	mCCDA
CH-0361	CA21CH-0361-6-2	*C. jejuni*	574	42	CAT	mCCDA
CH-0361	CA21CH-0361-6-3	*C. jejuni*	574	42	CAT	mCCDA
CH-0361	CA21CH-0361-6-4	*C. jejuni*	574	42	CAT	u-mCCDA
CH-0361	CA21CH-0361-6-5	*C. jejuni*	574	42	CAT	u-mCCDA
CH-0361	CA21CH-0361-6-6	*C. jejuni*	51	42	CAT	u-mCCDA

CAT = cefoperazone, amphotericin B, teicoplanin; mCCDA = modified charcoal-ceofperazone-deoxycholate agar; u-mCCDA = unsupplemented modified charcoal-ceofperazone-deoxycholate agar

Table S4: Summary table of *Campylobacter* growth (1) or no growth (0) from each culture condition combination for each sample, used as the input for mixed effects logistic regression models

Sample	Temperature	Broth	Plate	Growth
CH-0312	37	None	mCCDA	0
CH-0312	37	None	u-mCCDA	0
CH-0312	42	None	mCCDA	0
CH-0312	42	None	u-mCCDA	0
CH-0312	37	Bolton	mCCDA	0
CH-0312	37	Bolton	u-mCCDA	0
CH-0312	42	Bolton	mCCDA	0
CH-0312	42	Bolton	u-mCCDA	0
CH-0312	37	CAT	mCCDA	0
CH-0312	37	CAT	u-mCCDA	0
CH-0312	42	CAT	mCCDA	1
CH-0312	42	CAT	u-mCCDA	0
CH-0313	37	None	mCCDA	0
CH-0313	37	None	u-mCCDA	0
CH-0313	42	None	mCCDA	0
CH-0313	42	None	u-mCCDA	0
CH-0313	37	Bolton	mCCDA	0
CH-0313	37	Bolton	u-mCCDA	0
CH-0313	42	Bolton	mCCDA	0
CH-0313	42	Bolton	u-mCCDA	0
CH-0313	37	CAT	mCCDA	0
CH-0313	37	CAT	u-mCCDA	1
CH-0313	42	CAT	mCCDA	1
CH-0313	42	CAT	u-mCCDA	1
CH-0314	37	None	mCCDA	0
CH-0314	37	None	u-mCCDA	0
CH-0314	42	None	mCCDA	0
CH-0314	42	None	u-mCCDA	0
CH-0314	37	Bolton	mCCDA	0
CH-0314	37	Bolton	u-mCCDA	0
CH-0314	42	Bolton	mCCDA	0
CH-0314	42	Bolton	u-mCCDA	0
CH-0314	37	CAT	mCCDA	1

CH-0314	37	CAT	u-mCCDA	0
CH-0314	42	CAT	mCCDA	0
CH-0314	42	CAT	u-mCCDA	1
CH-0315	37	None	mCCDA	1
CH-0315	37	None	u-mCCDA	0
CH-0315	42	None	mCCDA	0
CH-0315	42	None	u-mCCDA	0
CH-0315	37	Bolton	mCCDA	0
CH-0315	37	Bolton	u-mCCDA	0
CH-0315	42	Bolton	mCCDA	0
CH-0315	42	Bolton	u-mCCDA	0
CH-0315	37	CAT	mCCDA	0
CH-0315	37	CAT	u-mCCDA	0
CH-0315	42	CAT	mCCDA	0
CH-0315	42	CAT	u-mCCDA	0
CH-0316	37	None	mCCDA	0
CH-0316	37	None	u-mCCDA	0
CH-0316	42	None	mCCDA	0
CH-0316	42	None	u-mCCDA	0
CH-0316	37	Bolton	mCCDA	0
CH-0316	37	Bolton	u-mCCDA	0
CH-0316	42	Bolton	mCCDA	0
CH-0316	42	Bolton	u-mCCDA	0
CH-0316	37	CAT	mCCDA	0
CH-0316	37	CAT	u-mCCDA	0
CH-0316	42	CAT	mCCDA	0
CH-0316	42	CAT	u-mCCDA	0
CH-0317	37	None	mCCDA	1
CH-0317	37	None	u-mCCDA	1
CH-0317	42	None	mCCDA	1
CH-0317	42	None	u-mCCDA	1
CH-0317	37	Bolton	mCCDA	1
CH-0317	37	Bolton	u-mCCDA	1
CH-0317	42	Bolton	mCCDA	1
CH-0317	42	Bolton	u-mCCDA	1
CH-0317	37	CAT	mCCDA	1
CH-0317	37	CAT	u-mCCDA	1
CH-0317	42	CAT	mCCDA	1
CH-0317	42	CAT	u-mCCDA	1
CH-0318	37	None	mCCDA	0
CH-0318	37	None	u-mCCDA	0
CH-0318	42	None	mCCDA	1
CH-0318	42	None	u-mCCDA	0
CH-0318	37	Bolton	mCCDA	0
CH-0318	37	Bolton	u-mCCDA	0
CH-0318	42	Bolton	mCCDA	0
CH-0318	42	Bolton	u-mCCDA	0
CH-0318	37	CAT	mCCDA	1
CH-0318	37	CAT	u-mCCDA	0

CH-0318	42	CAT	mCCDA	0
CH-0318	42	CAT	u-mCCDA	0
CH-0319	37	None	mCCDA	0
CH-0319	37	None	u-mCCDA	0
CH-0319	42	None	mCCDA	0
CH-0319	42	None	u-mCCDA	0
CH-0319	37	Bolton	mCCDA	0
CH-0319	37	Bolton	u-mCCDA	0
CH-0319	42	Bolton	mCCDA	0
CH-0319	42	Bolton	u-mCCDA	0
CH-0319	37	CAT	mCCDA	1
CH-0319	37	CAT	u-mCCDA	1
CH-0319	42	CAT	mCCDA	1
CH-0319	42	CAT	u-mCCDA	1
CH-0320	37	None	mCCDA	0
CH-0320	37	None	u-mCCDA	0
CH-0320	42	None	mCCDA	0
CH-0320	42	None	u-mCCDA	0
CH-0320	37	Bolton	mCCDA	1
CH-0320	37	Bolton	u-mCCDA	1
CH-0320	42	Bolton	mCCDA	0
CH-0320	42	Bolton	u-mCCDA	0
CH-0320	37	CAT	mCCDA	1
CH-0320	37	CAT	u-mCCDA	1
CH-0320	42	CAT	mCCDA	1
CH-0320	42	CAT	u-mCCDA	1
CH-0321	37	None	mCCDA	0
CH-0321	37	None	u-mCCDA	0
CH-0321	42	None	mCCDA	1
CH-0321	42	None	u-mCCDA	0
CH-0321	37	Bolton	mCCDA	0
CH-0321	37	Bolton	u-mCCDA	0
CH-0321	42	Bolton	mCCDA	0
CH-0321	42	Bolton	u-mCCDA	0
CH-0321	37	CAT	mCCDA	1
CH-0321	37	CAT	u-mCCDA	0
CH-0321	42	CAT	mCCDA	1
CH-0321	42	CAT	u-mCCDA	1
CH-0322	37	None	mCCDA	0
CH-0322	37	None	u-mCCDA	0
CH-0322	42	None	mCCDA	0
CH-0322	42	None	u-mCCDA	0
CH-0322	37	Bolton	mCCDA	0
CH-0322	37	Bolton	u-mCCDA	0
CH-0322	42	Bolton	mCCDA	0
CH-0322	42	Bolton	u-mCCDA	0
CH-0322	37	CAT	mCCDA	0
CH-0322	37	CAT	u-mCCDA	0
CH-0322	42	CAT	mCCDA	0

CH-0322	42	CAT	u-mCCDA	0
CH-0323	37	None	mCCDA	0
CH-0323	37	None	u-mCCDA	0
CH-0323	42	None	mCCDA	0
CH-0323	42	None	u-mCCDA	0
CH-0323	37	Bolton	mCCDA	0
CH-0323	37	Bolton	u-mCCDA	0
CH-0323	42	Bolton	mCCDA	0
CH-0323	42	Bolton	u-mCCDA	0
CH-0323	37	CAT	mCCDA	0
CH-0323	37	CAT	u-mCCDA	0
CH-0323	42	CAT	mCCDA	1
CH-0323	42	CAT	u-mCCDA	1
CH-0324	37	None	mCCDA	0
CH-0324	37	None	u-mCCDA	0
CH-0324	42	None	mCCDA	0
CH-0324	42	None	u-mCCDA	0
CH-0324	37	Bolton	mCCDA	0
CH-0324	37	Bolton	u-mCCDA	0
CH-0324	42	Bolton	mCCDA	0
CH-0324	42	Bolton	u-mCCDA	0
CH-0324	37	CAT	mCCDA	0
CH-0324	37	CAT	u-mCCDA	0
CH-0324	42	CAT	mCCDA	0
CH-0324	42	CAT	u-mCCDA	0
CH-0325	37	None	mCCDA	0
CH-0325	37	None	u-mCCDA	0
CH-0325	42	None	mCCDA	0
CH-0325	42	None	u-mCCDA	0
CH-0325	37	Bolton	mCCDA	1
CH-0325	37	Bolton	u-mCCDA	0
CH-0325	42	Bolton	mCCDA	0
CH-0325	42	Bolton	u-mCCDA	0
CH-0325	37	CAT	mCCDA	1
CH-0325	37	CAT	u-mCCDA	1
CH-0325	42	CAT	mCCDA	1
CH-0325	42	CAT	u-mCCDA	1
CH-0326	37	None	mCCDA	0
CH-0326	37	None	u-mCCDA	0
CH-0326	42	None	mCCDA	0
CH-0326	42	None	u-mCCDA	0
CH-0326	37	Bolton	mCCDA	0
CH-0326	37	Bolton	u-mCCDA	0
CH-0326	42	Bolton	mCCDA	1
CH-0326	42	Bolton	u-mCCDA	1
CH-0326	37	CAT	mCCDA	1
CH-0326	37	CAT	u-mCCDA	0
CH-0326	42	CAT	mCCDA	1
CH-0326	42	CAT	u-mCCDA	0

CH-0327	37	None	mCCDA	0
CH-0327	37	None	u-mCCDA	0
CH-0327	42	None	mCCDA	0
CH-0327	42	None	u-mCCDA	0
CH-0327	37	Bolton	mCCDA	0
CH-0327	37	Bolton	u-mCCDA	0
CH-0327	42	Bolton	mCCDA	1
CH-0327	42	Bolton	u-mCCDA	1
CH-0327	37	CAT	mCCDA	0
CH-0327	37	CAT	u-mCCDA	0
CH-0327	42	CAT	mCCDA	1
CH-0327	42	CAT	u-mCCDA	1
CH-0328	37	None	mCCDA	0
CH-0328	37	None	u-mCCDA	0
CH-0328	42	None	mCCDA	0
CH-0328	42	None	u-mCCDA	0
CH-0328	37	Bolton	mCCDA	0
CH-0328	37	Bolton	u-mCCDA	0
CH-0328	42	Bolton	mCCDA	1
CH-0328	42	Bolton	u-mCCDA	1
CH-0328	37	CAT	mCCDA	1
CH-0328	37	CAT	u-mCCDA	0
CH-0328	42	CAT	mCCDA	1
CH-0328	42	CAT	u-mCCDA	0
CH-0329	37	None	mCCDA	0
CH-0329	37	None	u-mCCDA	0
CH-0329	42	None	mCCDA	0
CH-0329	42	None	u-mCCDA	0
CH-0329	37	Bolton	mCCDA	1
CH-0329	37	Bolton	u-mCCDA	1
CH-0329	42	Bolton	mCCDA	1
CH-0329	42	Bolton	u-mCCDA	1
CH-0329	37	CAT	mCCDA	1
CH-0329	37	CAT	u-mCCDA	1
CH-0329	42	CAT	mCCDA	1
CH-0329	42	CAT	u-mCCDA	1
CH-0330	37	None	mCCDA	0
CH-0330	37	None	u-mCCDA	0
CH-0330	42	None	mCCDA	0
CH-0330	42	None	u-mCCDA	0
CH-0330	37	Bolton	mCCDA	1
CH-0330	37	Bolton	u-mCCDA	1
CH-0330	42	Bolton	mCCDA	1
CH-0330	42	Bolton	u-mCCDA	1
CH-0330	37	CAT	mCCDA	0
CH-0330	37	CAT	u-mCCDA	0
CH-0330	42	CAT	mCCDA	1
CH-0330	42	CAT	u-mCCDA	1
CH-0331	37	None	mCCDA	0

CH-0331	37	None	u-mCCDA	0
CH-0331	42	None	mCCDA	0
CH-0331	42	None	u-mCCDA	0
CH-0331	37	Bolton	mCCDA	1
CH-0331	37	Bolton	u-mCCDA	1
CH-0331	42	Bolton	mCCDA	1
CH-0331	42	Bolton	u-mCCDA	1
CH-0331	37	CAT	mCCDA	0
CH-0331	37	CAT	u-mCCDA	0
CH-0331	42	CAT	mCCDA	1
CH-0331	42	CAT	u-mCCDA	1
CH-0332	37	None	mCCDA	0
CH-0332	37	None	u-mCCDA	0
CH-0332	42	None	mCCDA	0
CH-0332	42	None	u-mCCDA	0
CH-0332	37	Bolton	mCCDA	0
CH-0332	37	Bolton	u-mCCDA	0
CH-0332	42	Bolton	mCCDA	0
CH-0332	42	Bolton	u-mCCDA	0
CH-0332	37	CAT	mCCDA	1
CH-0332	37	CAT	u-mCCDA	1
CH-0332	42	CAT	mCCDA	1
CH-0332	42	CAT	u-mCCDA	1
CH-0333	37	None	mCCDA	1
CH-0333	37	None	u-mCCDA	1
CH-0333	42	None	mCCDA	1
CH-0333	42	None	u-mCCDA	0
CH-0333	37	Bolton	mCCDA	1
CH-0333	37	Bolton	u-mCCDA	1
CH-0333	42	Bolton	mCCDA	1
CH-0333	42	Bolton	u-mCCDA	1
CH-0333	37	CAT	mCCDA	0
CH-0333	37	CAT	u-mCCDA	0
CH-0333	42	CAT	mCCDA	1
CH-0333	42	CAT	u-mCCDA	1
CH-0334	37	None	mCCDA	0
CH-0334	37	None	u-mCCDA	0
CH-0334	42	None	mCCDA	1
CH-0334	42	None	u-mCCDA	0
CH-0334	37	Bolton	mCCDA	1
CH-0334	37	Bolton	u-mCCDA	1
CH-0334	42	Bolton	mCCDA	1
CH-0334	42	Bolton	u-mCCDA	1
CH-0334	37	CAT	mCCDA	0
CH-0334	37	CAT	u-mCCDA	0
CH-0334	42	CAT	mCCDA	1
CH-0334	42	CAT	u-mCCDA	1
CH-0335	37	None	mCCDA	1
CH-0335	37	None	u-mCCDA	0

CH-0335	42	None	mCCDA	1
CH-0335	42	None	u-mCCDA	0
CH-0335	37	Bolton	mCCDA	1
CH-0335	37	Bolton	u-mCCDA	1
CH-0335	42	Bolton	mCCDA	1
CH-0335	42	Bolton	u-mCCDA	1
CH-0335	37	CAT	mCCDA	1
CH-0335	37	CAT	u-mCCDA	1
CH-0335	42	CAT	mCCDA	1
CH-0335	42	CAT	u-mCCDA	1
CH-0336	37	None	mCCDA	0
CH-0336	37	None	u-mCCDA	0
CH-0336	42	None	mCCDA	0
CH-0336	42	None	u-mCCDA	0
CH-0336	37	Bolton	mCCDA	1
CH-0336	37	Bolton	u-mCCDA	1
CH-0336	42	Bolton	mCCDA	1
CH-0336	42	Bolton	u-mCCDA	1
CH-0336	37	CAT	mCCDA	1
CH-0336	37	CAT	u-mCCDA	1
CH-0336	42	CAT	mCCDA	1
CH-0336	42	CAT	u-mCCDA	1
CH-0337	37	None	mCCDA	0
CH-0337	37	None	u-mCCDA	0
CH-0337	42	None	mCCDA	0
CH-0337	42	None	u-mCCDA	1
CH-0337	37	Bolton	mCCDA	1
CH-0337	37	Bolton	u-mCCDA	1
CH-0337	42	Bolton	mCCDA	1
CH-0337	42	Bolton	u-mCCDA	1
CH-0337	37	CAT	mCCDA	0
CH-0337	37	CAT	u-mCCDA	0
CH-0337	42	CAT	mCCDA	1
CH-0337	42	CAT	u-mCCDA	1
CH-0338	37	None	mCCDA	0
CH-0338	37	None	u-mCCDA	0
CH-0338	42	None	mCCDA	0
CH-0338	42	None	u-mCCDA	0
CH-0338	37	Bolton	mCCDA	1
CH-0338	37	Bolton	u-mCCDA	1
CH-0338	42	Bolton	mCCDA	1
CH-0338	42	Bolton	u-mCCDA	1
CH-0338	37	CAT	mCCDA	1
CH-0338	37	CAT	u-mCCDA	1
CH-0338	42	CAT	mCCDA	1
CH-0338	42	CAT	u-mCCDA	1
CH-0339	37	None	mCCDA	0
CH-0339	37	None	u-mCCDA	0
CH-0339	42	None	mCCDA	0

CH-0339	42	None	u-mCCDA	0
CH-0339	37	Bolton	mCCDA	1
CH-0339	37	Bolton	u-mCCDA	1
CH-0339	42	Bolton	mCCDA	1
CH-0339	42	Bolton	u-mCCDA	1
CH-0339	37	CAT	mCCDA	1
CH-0339	37	CAT	u-mCCDA	1
CH-0339	42	CAT	mCCDA	1
CH-0339	42	CAT	u-mCCDA	1
CH-0340	37	None	mCCDA	0
CH-0340	37	None	u-mCCDA	1
CH-0340	42	None	mCCDA	0
CH-0340	42	None	u-mCCDA	0
CH-0340	37	Bolton	mCCDA	1
CH-0340	37	Bolton	u-mCCDA	1
CH-0340	42	Bolton	mCCDA	1
CH-0340	42	Bolton	u-mCCDA	1
CH-0340	37	CAT	mCCDA	1
CH-0340	37	CAT	u-mCCDA	0
CH-0340	42	CAT	mCCDA	1
CH-0340	42	CAT	u-mCCDA	1
CH-0341	37	None	mCCDA	0
CH-0341	37	None	u-mCCDA	0
CH-0341	42	None	mCCDA	0
CH-0341	42	None	u-mCCDA	0
CH-0341	37	Bolton	mCCDA	1
CH-0341	37	Bolton	u-mCCDA	1
CH-0341	42	Bolton	mCCDA	1
CH-0341	42	Bolton	u-mCCDA	1
CH-0341	37	CAT	mCCDA	1
CH-0341	37	CAT	u-mCCDA	1
CH-0341	42	CAT	mCCDA	1
CH-0341	42	CAT	u-mCCDA	1
CH-0347	37	None	mCCDA	0
CH-0347	37	None	u-mCCDA	0
CH-0347	42	None	mCCDA	0
CH-0347	42	None	u-mCCDA	0
CH-0347	37	Bolton	mCCDA	0
CH-0347	37	Bolton	u-mCCDA	0
CH-0347	42	Bolton	mCCDA	1
CH-0347	42	Bolton	u-mCCDA	1
CH-0347	37	CAT	mCCDA	1
CH-0347	37	CAT	u-mCCDA	0
CH-0347	42	CAT	mCCDA	1
CH-0347	42	CAT	u-mCCDA	1
CH-0348	37	None	mCCDA	0
CH-0348	37	None	u-mCCDA	0
CH-0348	42	None	mCCDA	0
CH-0348	42	None	u-mCCDA	0

CH-0348	37	Bolton	mCCDA	0
CH-0348	37	Bolton	u-mCCDA	0
CH-0348	42	Bolton	mCCDA	0
CH-0348	42	Bolton	u-mCCDA	0
CH-0348	37	CAT	mCCDA	0
CH-0348	37	CAT	u-mCCDA	0
CH-0348	42	CAT	mCCDA	1
CH-0348	42	CAT	u-mCCDA	1
CH-0349	37	None	mCCDA	0
CH-0349	37	None	u-mCCDA	0
CH-0349	42	None	mCCDA	0
CH-0349	42	None	u-mCCDA	0
CH-0349	37	Bolton	mCCDA	1
CH-0349	37	Bolton	u-mCCDA	1
CH-0349	42	Bolton	mCCDA	1
CH-0349	42	Bolton	u-mCCDA	1
CH-0349	37	CAT	mCCDA	0
CH-0349	37	CAT	u-mCCDA	0
CH-0349	42	CAT	mCCDA	1
CH-0349	42	CAT	u-mCCDA	1
CH-0350	37	None	mCCDA	0
CH-0350	37	None	u-mCCDA	0
CH-0350	42	None	mCCDA	0
CH-0350	42	None	u-mCCDA	0
CH-0350	37	Bolton	mCCDA	1
CH-0350	37	Bolton	u-mCCDA	1
CH-0350	42	Bolton	mCCDA	1
CH-0350	42	Bolton	u-mCCDA	1
CH-0350	37	CAT	mCCDA	1
CH-0350	37	CAT	u-mCCDA	0
CH-0350	42	CAT	mCCDA	1
CH-0350	42	CAT	u-mCCDA	1
CH-0351	37	None	mCCDA	1
CH-0351	37	None	u-mCCDA	0
CH-0351	42	None	mCCDA	0
CH-0351	42	None	u-mCCDA	0
CH-0351	37	Bolton	mCCDA	1
CH-0351	37	Bolton	u-mCCDA	1
CH-0351	42	Bolton	mCCDA	1
CH-0351	42	Bolton	u-mCCDA	1
CH-0351	37	CAT	mCCDA	1
CH-0351	37	CAT	u-mCCDA	0
CH-0351	42	CAT	mCCDA	1
CH-0351	42	CAT	u-mCCDA	0
CH-0352	37	None	mCCDA	0
CH-0352	37	None	u-mCCDA	0
CH-0352	42	None	mCCDA	0
CH-0352	42	None	u-mCCDA	0
CH-0352	37	Bolton	mCCDA	0

CH-0352	37	Bolton	u-mCCDA	0
CH-0352	42	Bolton	mCCDA	0
CH-0352	42	Bolton	u-mCCDA	0
CH-0352	37	CAT	mCCDA	0
CH-0352	37	CAT	u-mCCDA	0
CH-0352	42	CAT	mCCDA	0
CH-0352	42	CAT	u-mCCDA	0
CH-0353	37	None	mCCDA	0
CH-0353	37	None	u-mCCDA	0
CH-0353	42	None	mCCDA	0
CH-0353	42	None	u-mCCDA	0
CH-0353	37	Bolton	mCCDA	1
CH-0353	37	Bolton	u-mCCDA	1
CH-0353	42	Bolton	mCCDA	1
CH-0353	42	Bolton	u-mCCDA	1
CH-0353	37	CAT	mCCDA	1
CH-0353	37	CAT	u-mCCDA	1
CH-0353	42	CAT	mCCDA	1
CH-0353	42	CAT	u-mCCDA	1
CH-0354	37	None	mCCDA	0
CH-0354	37	None	u-mCCDA	0
CH-0354	42	None	mCCDA	0
CH-0354	42	None	u-mCCDA	0
CH-0354	37	Bolton	mCCDA	0
CH-0354	37	Bolton	u-mCCDA	0
CH-0354	42	Bolton	mCCDA	0
CH-0354	42	Bolton	u-mCCDA	0
CH-0354	37	CAT	mCCDA	0
CH-0354	37	CAT	u-mCCDA	0
CH-0354	42	CAT	mCCDA	0
CH-0354	42	CAT	u-mCCDA	0
CH-0355	37	None	mCCDA	0
CH-0355	37	None	u-mCCDA	0
CH-0355	42	None	mCCDA	0
CH-0355	42	None	u-mCCDA	0
CH-0355	37	Bolton	mCCDA	0
CH-0355	37	Bolton	u-mCCDA	0
CH-0355	42	Bolton	mCCDA	1
CH-0355	42	Bolton	u-mCCDA	1
CH-0355	37	CAT	mCCDA	1
CH-0355	37	CAT	u-mCCDA	0
CH-0355	42	CAT	mCCDA	0
CH-0355	42	CAT	u-mCCDA	0
CH-0356	37	None	mCCDA	0
CH-0356	37	None	u-mCCDA	0
CH-0356	42	None	mCCDA	0
CH-0356	42	None	u-mCCDA	0
CH-0356	37	Bolton	mCCDA	0
CH-0356	37	Bolton	u-mCCDA	0

CH-0356	42	Bolton	mCCDA	0
CH-0356	42	Bolton	u-mCCDA	0
CH-0356	37	CAT	mCCDA	0
CH-0356	37	CAT	u-mCCDA	0
CH-0356	42	CAT	mCCDA	1
CH-0356	42	CAT	u-mCCDA	1
CH-0357	37	None	mCCDA	0
CH-0357	37	None	u-mCCDA	0
CH-0357	42	None	mCCDA	0
CH-0357	42	None	u-mCCDA	0
CH-0357	37	Bolton	mCCDA	1
CH-0357	37	Bolton	u-mCCDA	1
CH-0357	42	Bolton	mCCDA	1
CH-0357	42	Bolton	u-mCCDA	1
CH-0357	37	CAT	mCCDA	1
CH-0357	37	CAT	u-mCCDA	0
CH-0357	42	CAT	mCCDA	1
CH-0357	42	CAT	u-mCCDA	1
CH-0358	37	None	mCCDA	0
CH-0358	37	None	u-mCCDA	0
CH-0358	42	None	mCCDA	0
CH-0358	42	None	u-mCCDA	0
CH-0358	37	Bolton	mCCDA	0
CH-0358	37	Bolton	u-mCCDA	0
CH-0358	42	Bolton	mCCDA	1
CH-0358	42	Bolton	u-mCCDA	1
CH-0358	37	CAT	mCCDA	0
CH-0358	37	CAT	u-mCCDA	0
CH-0358	42	CAT	mCCDA	0
CH-0358	42	CAT	u-mCCDA	0
CH-0359	37	None	mCCDA	0
CH-0359	37	None	u-mCCDA	0
CH-0359	42	None	mCCDA	0
CH-0359	42	None	u-mCCDA	0
CH-0359	37	Bolton	mCCDA	1
CH-0359	37	Bolton	u-mCCDA	1
CH-0359	42	Bolton	mCCDA	1
CH-0359	42	Bolton	u-mCCDA	1
CH-0359	37	CAT	mCCDA	1
CH-0359	37	CAT	u-mCCDA	1
CH-0359	42	CAT	mCCDA	1
CH-0359	42	CAT	u-mCCDA	1
CH-0360	37	None	mCCDA	0
CH-0360	37	None	u-mCCDA	0
CH-0360	42	None	mCCDA	0
CH-0360	42	None	u-mCCDA	0
CH-0360	37	Bolton	mCCDA	0
CH-0360	37	Bolton	u-mCCDA	0
CH-0360	42	Bolton	mCCDA	0

CH-0360	42	Bolton	u-mCCDA	0
CH-0360	37	CAT	mCCDA	0
CH-0360	37	CAT	u-mCCDA	0
CH-0360	42	CAT	mCCDA	0
CH-0360	42	CAT	u-mCCDA	0
CH-0361	37	None	mCCDA	0
CH-0361	37	None	u-mCCDA	0
CH-0361	42	None	mCCDA	0
CH-0361	42	None	u-mCCDA	0
CH-0361	37	Bolton	mCCDA	1
CH-0361	37	Bolton	u-mCCDA	1
CH-0361	42	Bolton	mCCDA	1
CH-0361	42	Bolton	u-mCCDA	1
CH-0361	37	CAT	mCCDA	1
CH-0361	37	CAT	u-mCCDA	1
CH-0361	42	CAT	mCCDA	1
CH-0361	42	CAT	u-mCCDA	1

CAT = cefoperazone, amphotericin B, teicoplanin; mCCDA = modified charcoal-ceofperazone-deoxycholate agar; u-mCCDA = unsupplemented modified charcoal-ceofperazone-deoxycholate agar

Table S5A: Comparison tables for the agar plate types used for *Campylobacter* recovery

		u-mCCDA	
		0	1
mCCDA	0	148	4
	1	24	94

mCCDA = modified charcoal-ceofperazone-deoxycholate agar; u-mCCDA = unsupplemented modified charcoal-ceofperazone-deoxycholate agar

Table S5B: Comparison of the main effects logistic regression model using mCCDA data only (mCCDA_main) and a logistic regression model with an interaction term between Broth and Temperature (mCCDA_interaction), with the chicken sample modelled as a random effect; the values in the table represent odds ratios, confidence intervals and p-values

	mCCDA_main	mCCDA_interaction
BrothCAT	2·253 [1·040, 4·880] p=0·039 *	1·531 [0·536, 4·369] p=0·43
BrothNone	0·042 [0·015, 0·123] p=<0·001 ***	0·050 [0·012, 0·203] p=<0·001 ***
Temperature42	2·264 [1·134, 4·520] p=0·020 *	1·768 [0·616, 5·071] p=0·29
BrothCAT x Temperature42		2·359 [0·501, 11·114] p=0·28
BrothNone x Temperature42		0·745 [0·124, 4·494] p=0·75
Num.Obs.	270	270
R2 Marg.	0·325	0·325
R2 Cond.	0·657	0·664
AIC	279·1	281·2
BIC	297·1	306·4
ICC	0·5	0·5
RMSE	0·32	0·31

CAT = cefoperazone, amphotericin B, teicoplanin; mCCDA = modified charcoal-ceofperazone-deoxycholate agar

Table S5C: Model comparisons using ANOVA

Model	Model Type	Comparison	Df	AIC	logLik	*P*
mCCDA_main	Main effects model	Comparing main effects model to model with interaction	5	279·08	-134.54	
mCCDA_interaction	Interaction model		7	281·22	-133·61	0·39

mCCDA = modified charcoal-ceofperazone-deoxycholate agar

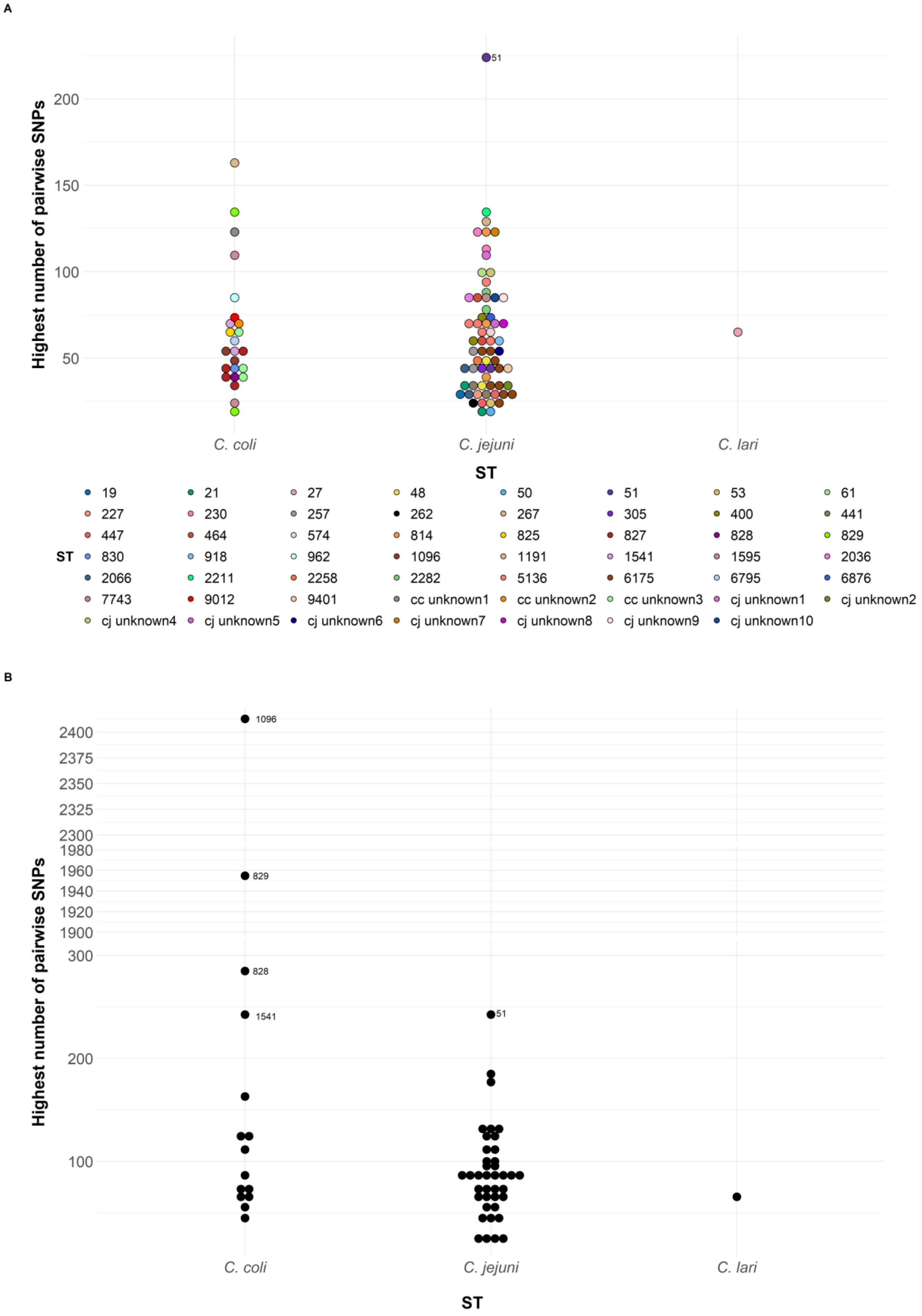

Figure S2: Highest non-recombinogenic pairwise SNP difference between isolates of individual STs* within individual chicken samples (A) and overall (B), with STs displaying differences of >200 SNPs annotated
* only ST groups consisting of more than one isolate displayed

Table S6: Pairwise non-recombinogenic SNP distances for *Campylobacter* STs identified in the study within samples and overall

ST	Species	Sample	Number of isolates	Pairwise SNPs	Median
		CH-0313	7	23-42	32
		CH-0334	1	N/A	N/A
		CH-0340	2	53	N/A
257	*C. jejuni*	**All**	10	23-92	53
		CH-0312	1	N/A	N/A
45	*C. jejuni*	**All**	1	N/A	N/A
		CH-0313	5	27-66	52
		CH-0318	8	37-94	65
		CH-0321	9	29-70	53
		CH-0331	9	24-70	45
		CH-0333	1	N/A	N/A
		CH-0336	3	52-62	58
5136	*C. jejuni*	**All**	35	24-115	66
		CH-0314	4	28-60	45
		CH-0320	8	26-84	49.5
464	*C. jejuni*	**All**	12	20-88	48.5
		CH-0314	2	34	N/A
		CH-0319	15	15-52	34
		CH-0321	5	23-50	32
		CH-0331	8	15-56	35
		CH-0335	1	N/A	N/A
		CH-0336	11	14-46	24
		CH-0338	2	22	N/A
		CH-0340	2	29	N/A
		CH-0341	1	N/A	N/A
		CH-0358	6	14-31	24
		CH-0359	3	28-34	33
6175	*C. jejuni*	**All**	56	9-72	34
		CH-0315	2	53	N/A
cj unknown6	*C. jejuni*	**All**	2	53	N/A
		CH-0317	45	13-74	41
		CH-0336	5	23-59	41.5
		CH-0337	1	N/A	N/A
400	*C. jejuni*	**All**	51	11-74	41
		CH-0319	1	N/A	N/A
122	*C. jejuni*	**All**	1	N/A	N/A
		CH-0320	16	14-65	39
27	*C. lari*	**All**	16	14-65	39
		CH-0323	7	63-123	89
cj unknown7	*C. jejuni*	**All**	7	63-123	89
		CH-0325	12	5-35	15
		CH-0335	1	N/A	N/A
		CH-0341	8	2-17	11
21	*C. jejuni*	**All**	21	2-89	33
		CH-0325	1	N/A	N/A
cc unknown4	*C. coli*	**All**	1	N/A	N/A
		CH-0325	3	42-48	44
		CH-0327	8	8-53	29.5
1096	*C. coli*	**All**	11	8-2413	44
		CH-0326	15	23-111	56
		CH-0341	2	24	N/A
1595	*C. coli*	**All**	17	23-128	72.5
		CH-0327	7	49-88	60
		CH-0331	7	27-78	54
2282	*C. jejuni*	**All**	14	19-90	59
		CH-0328	6	43-99	68
cj unknown4	*C. jejuni*	**All**	6	43-99	68
		CH-0328	1	N/A	N/A
		CH-0355	9	11-42	28
9401	*C. jejuni*	**All**	10	11-100	30
		CH-0328	10	43-124	71
		CH-0349	6	13-37	28
		CH-0353	18	17-68	33
814	*C. jejuni*	**All**	34	13-125	49

		CH-0329	31	19-135	67
		CH-0351	2	17	N/A
829	*C. coli*	**All**	33	17-1955	70
		CH-0329	1	N/A	N/A
		CH-0338	4	27-41	32.5
828	*C. coli*	**All**	5	27-285	38
		CH-0330	17	21-163	51.5
1191	*C. coli*	**All**	17	21-163	51.5
		CH-0330	4	21-31	27
		CH-0340	13	14-35	23
441	*C. jejuni*	**All**	17	14-54	28
		CH-0330	3	32-46	41
830	*C. coli*	**All**	3	32-46	41
		CH-0332	8	15-46	29
		CH-0337	1	N/A	N/A
		CH-0339	16	11-244	30
		CH-0361	1	N/A	N/A
51	*C. jejuni*	**All**	26	11-244	63
		CH-0332	8	25-100	46
61	*C. jejuni*	**All**	8	25-100	46
		CH-0333	2	108	N/A
cj unknown1	*C. jejuni*	**All**	2	108	N/A
		CH-0333	1	N/A	N/A
		CH-0350	3	16-33	31
		CH-0351	2	49	N/A
48	*C. jejuni*	**All**	6	16-132	42
		CH-0333	2	36	N/A
		CH-0334	1	N/A	N/A
cj unknown2	*C. jejuni*	**All**	3	36-64	53
		CH-0333	1	N/A	N/A
		CH-0334	17	0-22	10
53	*C. jejuni*	**All**	18	0-22	10
		CH-0333	15	38-129	78
267	*C. jejuni*	**All**	15	38-129	78
		CH-0333	1	N/A	N/A
cj unknown3	*C. jejuni*	**All**	1	N/A	N/A
		CH-0333	2	74	N/A
9012	*C. coli*	**All**	2	74	N/A
		CH-0334	4	32-113	90.5
		CH-0336	7	44-125	67
230	*C. jejuni*	**All**	11	32-185	103
		CH-0334	1	N/A	N/A
449	*C. jejuni*	**All**	1	N/A	N/A
		CH-0335	29	20-121	41
cc unknown1	*C. coli*	**All**	29	20-121	41
		CH-0335	3	22-28	24
227	*C. jejuni*	**All**	3	22-28	24
		CH-0335	1	N/A	N/A
2254	*C. jejuni*	**All**	1	N/A	N/A
		CH-0336	5	47-83	67
7743	*C. jejuni*	**All**	5	47-83	67
		CH-0337	16	9-43	28
		CH-0350	4	20-30	27.5
2066	*C. jejuni*	**All**	20	9-43	28
		CH-0337	3	62-134	120
2211	*C. jejuni*	**All**	3	62-134	120
		CH-0338	6	34-73	43
6876	*C. jejuni*	**All**	6	34-73	43
		CH-0338	1	N/A	N/A
		CH-0350	4	13-21	16.5
50	*C. jejuni*	**All**	5	13-177	20
		CH-0338	12	20-63	40
		CH-0340	7	19-45	36
		CH-0341	3	28-41	36
cc unknown3	*C. coli*	**All**	22	19-64	40
		CH-0338	7	34-71	60
		CH-0361	6	19-54	37
1541	*C. coli*	**All**	13	19-241	210.5

		CH-0339	8	31-69	48
cj unknown5	*C. jejuni*	**All**	8	31-69	48
		CH-0339	3	30-47	33
2258	*C. jejuni*	**All**	3	30-47	33
		CH-0339	4	32-58	48.5
6795	*C. coli*	**All**	4	32-58	48.5
		CH-0340	2	25	N/A
		CH-0341	2	29	N/A
447	*C. jejuni*	**All**	4	25-86	70
		CH-0340	2	32	N/A
		CH-0341	15	14-42	29
		CH-0348	6	18-40	36
		CH-0353	6	29-53	43
827	*C. coli*	**All**	29	14-110	77
		CH-0341	1	N/A	N/A
		CH-0351	3	46-72	62
cj unknown8	*C. jejuni*	**All**	4	46-76	59.5
		CH-0347	15	1-26	11
262	*C. jejuni*	**All**	15	1-26	11
		CH-0349	12	17-70	33
cc unknown2	*C. coli*	**All**	12	17-70	33
		CH-0350	9	5-27	16
19	*C. jejuni*	**All**	9	5-27	16
		CH-0350	1	N/A	N/A
8334	*C. jejuni*	**All**	1	N/A	N/A
		CH-0351	10	17-64	48
825	*C. coli*	**All**	10	17-64	48
		CH-0356	6	24-43	35
305	*C. jejuni*	**All**	6	24-43	35
		CH-0357	6	14-86	44
cj unknown9	*C. jejuni*	**All**	6	14-86	44
		CH-0357	9	19-62	43
918	*C. jejuni*	**All**	9	19-62	43
		CH-0357	6	38-85	53
2036	*C. jejuni*	**All**	6	38-85	53
		CH-0359	20	17-85	39
962	*C. coli*	**All**	20	17-85	39
		CH-0361	6	41-87	69
cj unknown10	*C. jejuni*	**All**	6	41-87	69
		CH-0361	11	23-67	41
574	*C. jejuni*	**All**	11	23-67	41

Table S7: Antimicrobial resistance determinants identified in the genomes of *Campylobacter jejuni*, *C. coli* and *C. lari* isolates recovered

Species	Number of isolates	Antimicrobial group	Number of isolates with at least one resistance determinant in the antimicrobial group	AMR determinant	Nonsynonymous mutations identified	Number (%) of isolates positive for determinant	% all isolates
C. jejuni	499	Beta-lactam	409				
				bla_{OXA-61}		6 (1·2)	0·8
				$bla_{OXA-184}$		65 (13·0)	8·7
				$bla_{OXA-185}$		18 (3·6)	2·4
				$bla_{OXA-193}$		310 (62·1)	41·7
				$bla_{OXA-447}$		1 (0·2)	0·1
				$bla_{OXA-465}$		9 (1·8)	1·2
		Tetracycline	313	*tet(O)*		204 (40·9)	27·5
				tet(O/32/O)		109 (21·8)	14·7
		Aminoglycoside	6	*ant(6)-Ia*		6 (1·2)	0·8
		Quinolone	338	*gyrA* mutation	T86I/T86I+P104S	338 (67·7)	45·5
C. coli	228	Beta-lactam	127				
				$bla_{OXA-193}$		36 (15·8)	4·8
				$bla_{OXA-452}$		12 (5·3)	1·6
				$bla_{OXA-453}$		22 (9·6)	3·0
				$bla_{OXA-489}$		57 (25·0)	7·7
		Tetracycline	79	*tet(O)*		76 (33·3)	10·2
				tet(O/32/O)		3 (1·3)	0·4
		Aminoglycoside	33	*aadE-Cc*		33 (14·5)	4·4
		Quinolone	44	*gyrA* mutation	T86I	44 (19·3)	5·9
C. lari	16	Beta-lactam	16	$bla_{OXA-493}$		16 (100)	2·2

Table S8: *Campylobacter* ST groups of isolates recovered from individual chicken samples and their AMR genotypes

Sample	Species	ST	Number of isolates	AMR determinants	% isolates positive for determinant
CH-0312	*C. jejuni*	45	1	$bla_{OXA-447}$	100
CH-0313	*C. jejuni*	257	7	$bla_{OXA-193}$	100
		5136	5	$bla_{OXA-193}$	100
				tet(O/32/O)	100
				gyrA mutation (T86I)	100
CH-0314	*C. jejuni*	464	4	$bla_{OXA-193}$	100
				tet(O/32/O)	100
				gyrA mutation (T86I)	100
		6175	2	$bla_{OXA-193}$	100
				tet(O)	100
				gyrA mutation (T86I)	100
CH-0315	*C. jejuni*	cj unknown6	2	$bla_{OXA-184}$	100
				tet(O)	100
				gyrA mutation (T86I)	100
CH-0317	*C. jejuni*	400	45	*tet(O/32/O)*	100
				gyrA mutation (T86I)	100
CH-0318	*C. jejuni*	5136	8	$bla_{OXA-193}$	100
				tet(O/32/O)	100
				gyrA mutation (T86I)	100
CH-0319	*C. jejuni*	122	1	$bla_{OXA-193}$	100
		6175	15	$bla_{OXA-193}$	100
				tet(O)	100
				gyrA mutation (T86I)	100
CH-0320	*C. lari*	27	16	$bla_{OXA-493}$	100
	C. jejuni	464	8	$bla_{OXA-193}$	100
				tet(O/32/O)	100
				gyrA mutation (T86I)	100
CH-0321	*C. jejuni*	5136	9	$bla_{OXA-193}$	100
				tet(O/32/O)	100
				gyrA mutation (T86I)	100
		6175	5	$bla_{OXA-193}$	100
				tet(O)	100
				gyrA mutation (T86I)	100
CH-0323	*C. jejuni*	cj unknown7	7	$bla_{OXA-193}$	100
CH-0325	*C. jejuni*	21	12	$bla_{OXA-193}$	100
				gyrA mutation (T86I)	100
	C. coli	1096	3	*aadE-Cc*	100
		cc unknown4	1	*aadE-Cc*	100
CH-0326	*C. coli*	1595	15	$bla_{OXA-193}$	100
				tet(O)	100
CH-0327	*C. jejuni*	2282	7	$bla_{OXA-193}$	85·7
	C. coli	1096	8	NA	NA
CH-0328	*C. jejuni*	814	10	$bla_{OXA-184}$	100
				tet(O)	100
				gyrA mutation (T86I)	100
		9401	1	*gyrA* mutation (T86I)	100
		cj unknown4	6	$bla_{OXA-184}$	100
				tet(O)	100
CH-0329	*C. coli*	828	1	*tet(O)*	100
				gyrA mutation (T86I)	100
		829	31	NA	NA
CH-0330	*C. jejuni*	441	4	$bla_{OXA-193}$	100
				gyrA mutation (T86I, P104S)	100
	C. coli	830	3	$bla_{OXA-193}$	100
				tet(O/32/O)	100
				gyrA mutation (T86I)	100
		1191	17	$bla_{OXA-193}$	94·1
CH-0331	*C. jejuni*	2282	7	$bla_{OXA-193}$	100
		5136	9	$bla_{OXA-193}$	100
				tet(O/32/O)	100
				gyrA mutation (T86I)	100

		6175	8	*bla*$_{OXA-193}$<br>*tet(O)*<br>*gyrA* mutation (T86I)	87·5<br>100<br>100
CH-0332	*C. jejuni*	51	8	*bla*$_{OXA-193}$<br>*tet(O)*	100<br>100
		61	8	*bla*$_{OXA-193}$<br>*gyrA* mutation (T86I)	100<br>100
CH-0333	*C. jejuni*	48	1	*bla*$_{OXA-61}$	100
		53	1	*bla*$_{OXA-193}$	100
		267	15	*bla*$_{OXA-193}$	93·3
		5136	1	*bla*$_{OXA-193}$<br>*tet(O/32/O)*<br>*gyrA* mutation (T86I)	100<br>100<br>100
		cj unknown1	2	*bla*$_{OXA-184}$<br>*tet(O)*	100<br>100
		cj unkown2	2	*bla*$_{OXA-465}$<br>*tet(O)*	100<br>100
		cj unknown3	1	*bla*$_{OXA-184}$<br>*tet(O)*	100<br>100
	C. coli	9012	2	NA	NA
CH-0334	*C. jejuni*	53	17	*bla*$_{OXA-193}$	94·1
		230	4	*bla*$_{OXA-193}$	100
		257	1	*bla*$_{OXA-193}$	100
		449	1	*bla*$_{OXA-184}$<br>*tet(O)*<br>*gyrA* mutation (T86I)	100<br>100<br>100
		cj unknown2	1	*bla*$_{OXA-465}$<br>*tet(O)*	100<br>100
CH-0335	*C. jejuni*	21	1	*bla*$_{OXA-193}$	100
		227	3	*bla*$_{OXA-193}$	100
		2254	1	*bla*$_{OXA-193}$<br>*tet(O)*<br>*gyrA* mutation (T86I)	100<br>100<br>100
		6175	1	*bla*$_{OXA-193}$<br>*tet(O)*<br>*gyrA* mutation (T86I)	100<br>100<br>100
	C. coli	cc unknown1	29	*aadE-Cc*<br>*bla*$_{OXA-489}$	100<br>96·6
CH-0336	*C. jejuni*	230	7	*bla*$_{OXA-193}$	100
		400	5	*tet(O/32/O)*<br>*gyrA* mutation (T86I)	100<br>100
		5136	3	*bla*$_{OXA-193}$<br>*tet(O/32/O)*<br>*gyrA* mutation (T86I)	100<br>100<br>100
		6175	11	*bla*$_{OXA-193}$<br>*tet(O)*<br>*gyrA* mutation (T86I)	100<br>100<br>100
		7743	5	*bla*$_{OXA-184}$<br>*bla*$_{OXA-185}$<br>*gyrA* mutation (T86I)	20·0<br>80·0<br>100
CH-0337	*C. jejuni*	51	1	*bla*$_{OXA-184}$	100
		400	1	*tet(O/32/O)*<br>*gyrA* mutation (T86I)	100<br>100
		2066	16	*tet(O)*<br>*gyrA* mutation (T86I)	100<br>100
		2211	3	*bla*$_{OXA-184}$<br>*tet(O)*	100<br>100
CH-0338	*C. jejuni*	50	1	*bla*$_{OXA-193}$<br>*tet(O/32/O)*<br>*gyrA* mutation (T86I)	100<br>100<br>100
		6175	2	*bla*$_{OXA-193}$<br>*tet(O)*<br>*gyrA* mutation (T86I)	100<br>100<br>100
		6876	6	*ant(6)-Ia*<br>*bla*$_{OXA-465}$<br>*gyrA* mutation (T86I)	100<br>100<br>100

				tet(O)	100
	C. coli	828	4	*tet(O)*<br>*gyrA* mutation (T86I)	100<br>100
		1541	7	NA	NA
		cc unknown3	12	*bla*$_{OXA-453}$<br>*tet(O)*<br>*gyrA* mutation (T86I)	100<br>100<br>100
CH-0339	*C. jejuni*	51	16	*bla*$_{OXA-193}$<br>*bla*$_{OXA-184}$<br>*tet(O)*	87·5<br>12·5<br>87·5
		2258	3	*bla*$_{OXA-193}$	100
		cj unknown5	8	*bla*$_{OXA-185}$<br>*gyrA* mutation (T86I)	100<br>100
	C. coli	6795	4	*gyrA* mutation (T86I)	100
CH-0340	*C. jejuni*	257	2	*bla*$_{OXA-193}$<br>*tet(O)*	100<br>100
		441	13	*bla*$_{OXA-193}$<br>*gyrA* mutation (T86I, P104S)	100<br>100
		447	2	*tet(O)*	100
		6175	2	*bla*$_{OXA-193}$<br>*tet(O)*<br>*gyrA* mutation (T86I)	100<br>100<br>100
	C. coli	827	2	*bla*$_{OXA-489}$	100
		cc unknown3	7	*bla*$_{OXA-453}$<br>*tet(O)*<br>*gyrA* mutation (T86I)	100<br>100<br>100
CH-0341	*C. jejuni*	21	8	*bla*$_{OXA-193}$<br>*gyrA* mutation (T86I)	100<br>100
		447	2	NA	NA
		6175	1	*bla*$_{OXA-193}$<br>*tet(O)*<br>*gyrA* mutation (T86I)	100<br>100<br>100
		cj unknown8	1	*bla*$_{OXA-193}$<br>*tet(O/32/O)*<br>*gyrA* mutation (T86I)	100<br>100<br>100
	C. coli	827	15	*bla*$_{OXA-489}$	100
		1595	2	*bla*$_{OXA-193}$<br>*tet(O)*	100<br>100
		cc unknown3	3	*bla*$_{OXA-453}$<br>*tet(O)*<br>*gyrA* mutation (T86I)	100<br>100<br>100
CH-0347	*C. jejuni*	262	15	*bla*$_{OXA-193}$<br>*tet(O)*	100<br>100
CH-0348	*C. coli*	827	6	*bla*$_{OXA-489}$	100
CH-0349	*C. jejuni*	814	6	*bla*$_{OXA-184}$<br>*tet(O)*<br>*gyrA* mutation (T86I)	100<br>100<br>100
	C. coli	cc unknown2	12	*bla*$_{OXA-452}$<br>*tet(O)*	100<br>100
CH-0350	*C. jejuni*	19	9	*bla*$_{OXA-193}$<br>*gyrA* mutation (T86I)	100<br>100
		48	3	*bla*$_{OXA-61}$<br>*tet(O)*	100<br>100
		50	4	*bla*$_{OXA-193}$<br>*tet(O)*<br>*gyrA* mutation (T86I)	100<br>100<br>100
		2066	4	*tet(O)*<br>*gyrA* mutation (T86I)	100<br>100
		8334	1	*tet(O)*<br>*gyrA* mutation (T86I)	100<br>100
CH-0351	*C. jejuni*	48	2	*bla*$_{OXA-61}$<br>*tet(O)*	100<br>100
		cj unknown8	3	*bla*$_{OXA-193}$<br>*gyrA* mutation (T86I)<br>*tet(O/32/O)*	100<br>100<br>100
	C. coli	825	10	*gyrA* mutation (T86I)	100
		829	2	NA	NA
CH-0353	*C. jejuni*	814	18	*bla*$_{OXA-184}$<br>*tet(O)*<br>*gyrA* mutation (T86I)	100<br>100<br>100

	C. coli	827	6	*bla*$_{OXA-489}$	100
CH-0355	*C. jejuni*	9401	9	*gyrA* mutation (T86I)	100
CH-0356	*C. jejuni*	305	6	*bla*$_{OXA-193}$	100
				gyrA mutation (T86I)	100
CH-0357	*C. jejuni*	918	9	*bla*$_{OXA-193}$	100
				gyrA mutation (T86I)	100
		2036	6	*bla*$_{OXA-193}$	100
				tet(O/32/O)	100
				gyrA mutation (T86I)	100
		cj unkown9	6	*bla*$_{OXA-184}$	100
				tet(O)	100
				gyrA mutation (T86I)	100
CH-0358	*C. jejuni*	6175	6	*bla*$_{OXA-184}$	100
				gyrA mutation (T86I)	100
				tet(O)	100
CH-0359	*C. jejuni*	6175	3	*bla*$_{OXA-193}$	100
				tet(O)	100
				gyrA mutation (T86I)	100
	C. coli	962	20	*tet(O)*	100
CH-0361	*C. jejuni*	51	1	*bla*$_{OXA-193}$	100
				tet(O)	100
		574	11	*bla*$_{OXA-193}$	100
				tet(O)	100
		cj unknown10	6	*bla*$_{OXA-185}$	100
				gyrA mutation (T86I)	100
	C. coli	1541	6	NA	NA

Table S9: Intra-ST differences in AMR genotype within samples

Sample	Species	ST	AMR determinants	% isolates positive for determinant	Details
CH-0327	*C. jejuni*	2282	*bla*$_{OXA-193}$	85·7	*bla*$_{OXA-193}$ below coverage threshold (90%) in one genome
			None	14·3	
CH-0330	*C. coli*	1191	*bla*$_{OXA-193}$	94·1	*bla*$_{OXA-193}$ below coverage threshold (90%) in one genome
			None	5·9	
CH-0331	*C. jejuni*	6175	*bla*$_{OXA-193}$ + *tet(O)* + *gyrA* mutation (T86I)	87·5	*bla*$_{OXA-193}$ below coverage threshold (90%) in one genome
			tet(O) + *gyrA* mutation (T86I)	12·5	
CH-0333	*C. jejuni*	267	*bla*$_{OXA-193}$	93·3	*bla*$_{OXA-193}$ below coverage threshold (90%) in one genome
			None	6·7	
CH-0334	*C. jejuni*	53	*bla*$_{OXA-193}$	94·1	*bla*$_{OXA-193}$ below coverage threshold (90%) in one genome
			None	5·9	
CH-0335	*C. coli*	cc unknown1	*aadE-Cc* + *bla*$_{OXA-489}$	100	*bla*$_{OXA-489}$ below coverage threshold (90%) in one genome
			aadE-Cc	3·4	
CH-0336	*C. jejuni*	7743	*bla*$_{OXA-184}$	20·0	Different *bla*$_{OXA}$ gene identified in one genome
			bla$_{OXA-185}$	80·0	
CH-0339	*C. jejuni*	51	*bla*$_{OXA-193}$ + *tet(O)*	87·5	*bla*$_{OXA-193}$ and *tet(O)* genes were identified in 14 isolates, and *bla*$_{OXA-184}$ in two isolates without *tet(O)*
			bla$_{OXA-184}$	12·5	

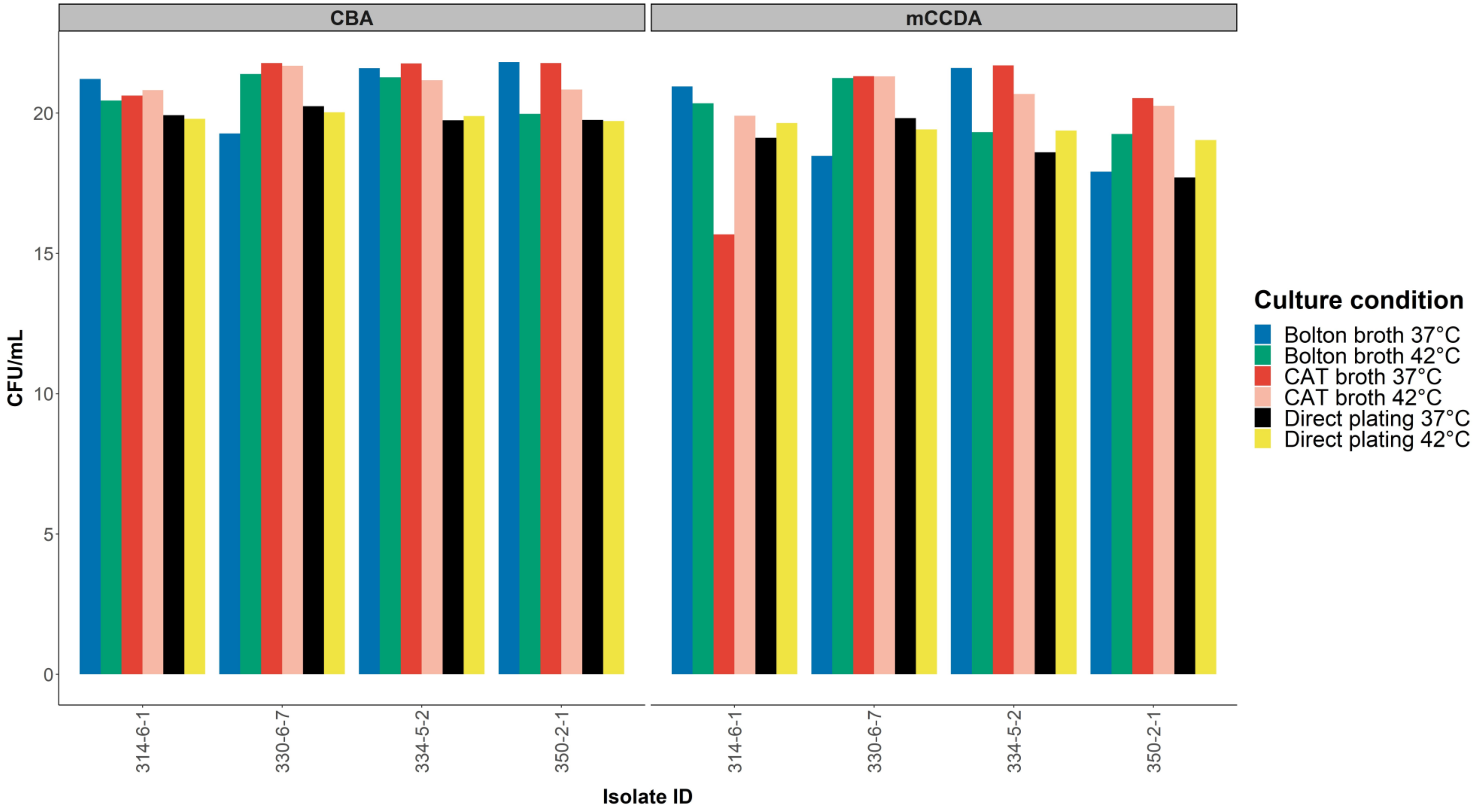

Figure S3: Comparison of growth (log CFU/mL) in different conditions tested in the study based on plate counts on CBA and mCCDA of four isolates representing four different STs previously identified through one condition (Bolton broth/CAT broth) only
CAT = cefoperazone, amphotericin B, teicoplanin, CBA = Columbia blood agar; mCCDA = modified charcoal-cefoperazone-deoxycholate agar

Table S10: Average plate counts and CFU/mL values for comparisons of growth of four isolates representing four STs previously identified through one condition only (Bolton broth/CAT broth)

Isolate	Condition	plate	Plate counts	Dilution	CFU/mL	Notes
314-6-1	Direct plating 37°C	mCCDA	26, 14	10^{-6}	2·00E+08	
314-6-1	Direct plating 42°C	mCCDA	31, 37	10^{-6}	3·40E+08	some colony swarming was observed, though enumeration was possible as the colonies were isolated
314-6-1	Bolton broth 37°C	mCCDA	133, 118	10^{-6}	1·26E+09	
314-6-1	Bolton broth 42°C	mCCDA	60, 77	10^{-6}	6·85E+08	
314-6-1	CAT broth 37°C	mCCDA	54, 74	10^{-4}	6·40E+06	
314-6-1	CAT broth 42°C	mCCDA	55, 33	10^{-6}	4·40E+08	
314-6-1	Direct plating 37°C	CBA	50, 40	10^{-6}	4·50E+08	
314-6-1	Direct plating 42°C	CBA	41, 38	10^{-6}	3·95E+08	some colony swarming was observed, though enumeration was possible as the colonies were isolated
314-6-1	Bolton broth 37°C	CBA	159, 167	10^{-6}	1·63E+09	
314-6-1	Bolton broth 42°C	CBA	75, 76	10^{-6}	7·55E+08	
314-6-1	CAT broth 37°C	CBA	72, 109	10^{-6}	9·05E+08	
314-6-1	CAT broth 42°C	CBA	98, 122	10^{-6}	1·10E+09	
330-6-7	Direct plating 37°C	mCCDA	43, 38	10^{-6}	4·05E+08	
330-6-7	Direct plating 42°C	mCCDA	30, 24	10^{-6}	2·70E+08	some colony swarming was observed, though enumeration was possible as the colonies were isolated
330-6-7	Bolton broth 37°C	mCCDA	7, 14	10^{-6}	1·05E+08	
330-6-7	Bolton broth 42°C	mCCDA	166, 172	10^{-6}	1·69E+09	
330-6-7	CAT broth 37°C	mCCDA	171, 190	10^{-6}	1·81E+09	
330-6-7	CAT broth 42°C	mCCDA	178, 180	10^{-6}	1·79E+09	
330-6-7	Direct plating 37°C	CBA	63, 61	10^{-6}	6·20E+08	
330-6-7	Direct plating 42°C	CBA	48, 52	10^{-6}	5·00E+08	some colony swarming was observed, though enumeration was possible as the colonies were isolated
330-6-7	Bolton broth 37°C	CBA	24, 23	10^{-6}	2·35E+08	
330-6-7	Bolton broth 42°C	CBA	215, 175	10^{-6}	1·95E+09	
330-6-7	CAT broth 37°C	CBA	271, 307	10^{-6}	2·89E+09	
330-6-7	CAT broth 42°C	CBA	272, 249	10^{-6}	2·61E+09	
334-5-2	Direct plating 37°C	mCCDA	8, 16	10^{-6}	1·20E+08	
334-5-2	Direct plating 42°C	mCCDA	26	10^{-6}	2·60E+08	only one plate count was available for calculation of CFU/mL due to swarming on the other plate, thus the results should be treated with caution
334-5-2	Bolton broth 37°C	mCCDA	247, 236	10^{-6}	2·42E+09	
334-5-2	Bolton broth 42°C	mCCDA	24, 25	10^{-6}	2·45E+08	
334-5-2	CAT broth 37°C	mCCDA	268, 260	10^{-6}	2·64E+09	
334-5-2	CAT broth 42°C	mCCDA	95, 97	10^{-6}	9·60E+08	
334-5-2	Direct plating 37°C	CBA	36, 39	10^{-6}	3·75E+08	
334-5-2	Direct plating 42°C	CBA	42, 45	10^{-6}	4·35E+08	
334-5-2	Bolton broth 37°C	CBA	245, 234	10^{-6}	2·40E+09	
334-5-2	Bolton broth 42°C	CBA	177, 169	10^{-6}	1·73E+09	
334-5-2	CAT broth 37°C	CBA	311, 258	10^{-6}	2·85E+09	
334-5-2	CAT broth 42°C	CBA	156, 156	10^{-6}	1·56E+09	
350-2-1	Direct plating 37°C	mCCDA	142, 101*	10^{-4}	4·86E+07	*too numerous to count - one quarter of plate counted and count multiplied to obtain CFU/mL

350-2-1	Direct plating 42°C	mCCDA	15, 22	10^{-6}	1·85E+08	
350-2-1	Bolton broth 37°C	mCCDA	147, 154*	10^{-4}	6·02E+07	*too numerous to count - one quarter of plate counted and count multiplied to obtain CFU/mL
350-2-1	Bolton broth 42°C	mCCDA	29, 17	10^{-6}	2·30E+08	
350-2-1	CAT broth 37°C	mCCDA	75, 90	10^{-6}	8·25E+08	
350-2-1	CAT broth 42°C	mCCDA	65, 60	10^{-6}	6·25E+08	
350-2-1	Direct plating 37°C	CBA	42, 34	10^{-6}	3·80E+08	
350-2-1	Direct plating 42°C	CBA	38, 35	10^{-6}	3·65E+08	
350-2-1	Bolton broth 37°C	CBA	74, 75*	10^{-6}	2·98E+09	*too numerous to count - one quarter of plate counted and count multiplied to obtain CFU/mL
350-2-1	Bolton broth 42°C	CBA	50, 44	10^{-6}	4·70E+08	
350-2-1	CAT broth 37°C	CBA	292, 283	10^{-6}	2·88E+09	
350-2-1	CAT broth 42°C	CBA	106, 118	10^{-6}	1·12E+09	

CAT = cefoperazone, amphotericin B, teicoplanin; CBA = Columbia blood agar; mCCDA = modified charcoal-ceofperazone-deoxycholate agar

References

1. **Chen S, *et al.*** fastp: an ultra-fast all-in-one FASTQ preprocessor. *Bioinformatics* 2018; **34**: i884–i890.

2. **Gurevich A, *et al.*** QUAST: quality assessment tool for genome assemblies. *Bioinformatics* 2013; **29**: 1072–1075.

3. **Parks DH, *et al.*** CheckM: assessing the quality of microbial genomes recovered from isolates, single cells, and metagenomes. *Genome Research* 2015; **25**: 1043–1055.

4. **Li H**. Aligning sequence reads, clone sequences and assembly contigs with BWA-MEM. *arXiv:1303.3997 [q-bio]* 2013; Published online: 26 May 2013. doi:https://doi.org/10.48550/arXiv.1303.3997.

5. **Bharat A, *et al.*** Correlation between phenotypic and in silico detection of antimicrobial resistance in *Salmonella enterica* in Canada using Staramr. *Microorganisms* 2022; **10**: 292.

6. **Zankari E, *et al.*** PointFinder: a novel web tool for WGS-based detection of antimicrobial resistance associated with chromosomal point mutations in bacterial pathogens. *Journal of Antimicrobial Chemotherapy* 2017; **72**: 2764–2768.

7. **Payot S, *et al.*** Mechanisms of fluoroquinolone and macrolide resistance in *Campylobacter* spp. *Microbes and Infection* 2006; **8**: 1967–1971.

8. **Dahl LG, *et al.*** Prediction of antimicrobial resistance in clinical *Campylobacter jejuni* isolates from whole-genome sequencing data. *European Journal of Clinical Microbiology & Infectious Diseases* 2021; **40**: 673–682.

9. **Schwengers O, *et al.*** Bakta: rapid and standardized annotation of bacterial genomes via alignment-free sequence identification. *Microbial Genomics*, 2021; **7**: 000685.

10. **Edgar RC**. MUSCLE: multiple sequence alignment with high accuracy and high throughput. *Nucleic Acids Research* 2004; **32**: 1792–1797.

11. **Gouy M, Guindon S, Gascuel O**. SeaView Version 4: A multiplatform graphical user interface for sequence alignment and phylogenetic tree building. *Molecular Biology and Evolution* 2010; **27**: 221–224.

12. **Gouy M, *et al.*** *Seaview Version 5: A Multiplatform Software for Multiple Sequence Alignment, Molecular Phylogenetic Analyses, and Tree Reconciliation*. In: Katoh K, ed. *Multiple Sequence Alignment: Methods and Protocols*. New York, NY: Springer US, 2021, p. 241–260.
